# Effect of Supplementation with Zinc and Other Micronutrients on Malaria in Tanzanian Children: A Randomised Trial

**DOI:** 10.1371/journal.pmed.1001125

**Published:** 2011-11-22

**Authors:** Jacobien Veenemans, Paul Milligan, Andrew M. Prentice, Laura R. A. Schouten, Nienke Inja, Aafke C. van der Heijden, Linsey C. C. de Boer, Esther J. S. Jansen, Anna E. Koopmans, Wendy T. M. Enthoven, Rob J. Kraaijenhagen, Ayse Y. Demir, Donald R. A. Uges, Erasto V. Mbugi, Huub F. J. Savelkoul, Hans Verhoef

**Affiliations:** 1 Wageningen University, Cell Biology and Immunology Group, Wageningen, The Netherlands; 2 Laboratory for Microbiology and Infection Control, Amphia Hospital, Breda, The Netherlands; 3 Department of Infectious Disease Epidemiology, London School of Hygiene and Tropical Medicine, London, United Kingdom; 4 MRC International Nutrition Group, London School of Hygiene and Tropical Medicine, London, United Kingdom; 5 MRC Keneba, The Gambia; 6 Meander Medical Centre, Laboratory for Clinical Chemistry and Haematology, Amersfoort, The Netherlands; 7 University of Groningen, University Medical Center, Department of Pharmacy, Laboratory for Clinical and Forensic Toxicology and Drug Analysis, Groningen, The Netherlands; 8 Muhimbili University of Health and Allied Sciences, Dar es Salaam, Tanzania; Menzies School of Health Research, Australia

## Abstract

**Background:**

It is uncertain to what extent oral supplementation with zinc can reduce episodes of malaria in endemic areas. Protection may depend on other nutrients. We measured the effect of supplementation with zinc and other nutrients on malaria rates.

**Methods and Findings:**

In a 2×2 factorial trial, 612 rural Tanzanian children aged 6–60 months in an area with intense malaria transmission and with height-for-age z-score≤−1.5 SD were randomized to receive daily oral supplementation with either zinc alone (10 mg), multi-nutrients without zinc, multi-nutrients with zinc, or placebo. Intervention group was indicated by colour code, but neither participants, researchers, nor field staff knew who received what intervention. Those with *Plasmodium* infection at baseline were treated with artemether-lumefantrine. The primary outcome, an episode of malaria, was assessed among children reported sick at a primary care clinic, and pre-defined as current *Plasmodium* infection with an inflammatory response, shown by axillary temperature ≥37.5°C or whole blood C-reactive protein concentration ≥8 mg/L. Nutritional indicators were assessed at baseline and at 251 days (median; 95% reference range: 191–296 days). In the primary intention-to-treat analysis, we adjusted for pre-specified baseline factors, using Cox regression models that accounted for multiple episodes per child. 592 children completed the study. The primary analysis included 1,572 malaria episodes during 526 child-years of observation (median follow-up: 331 days). Malaria incidence in groups receiving zinc, multi-nutrients without zinc, multi-nutrients with zinc and placebo was 2.89/child-year, 2.95/child-year, 3.26/child-year, and 2.87/child-year, respectively. There was no evidence that multi-nutrients influenced the effect of zinc (or vice versa). Neither zinc nor multi-nutrients influenced malaria rates (marginal analysis; adjusted HR, 95% CI: 1.04, 0.93–1.18 and 1.10, 0.97–1.24 respectively). The prevalence of zinc deficiency (plasma zinc concentration <9.9 µmol/L) was high at baseline (67% overall; 60% in those without inflammation) and strongly reduced by zinc supplementation.

**Conclusions:**

We found no evidence from this trial that zinc supplementation protected against malaria.

**Trial Registration:**

ClinicalTrials.gov NCT00623857

*Please see later in the article for the Editors' Summary.*

## Abstract 2

Hans Verhoef and colleagues report findings from a randomized trial conducted among Tanzanian children at high risk for malaria. Children in the trial received either daily oral supplementation with either zinc alone, multi-nutrients without zinc, multi-nutrients with zinc, or placebo. The investigators did not find evidence from this study that zinc or multi-nutrients protected against malaria episodes.

## Editors' Summary

**Background:**

Malaria is a serious global public-health problem. Half of the world's population is at risk of this parasitic disease, which kills a million people (mainly children living in sub-Saharan Africa) every year. Malaria is transmitted to people through the bites of infected night-flying mosquitoes. Soon after entering the human body, the parasite begins to replicate in red blood cells, bursting out every 2–3 days and infecting more red blood cells. The presence of the parasite in the blood stream (parasitemia) causes malaria's characteristic recurring fever and can cause life-threatening organ damage and anemia (insufficient quantity of red blood cells). Malaria transmission can be reduced by using insecticide sprays to control the mosquitoes that spread the parasite and by avoiding mosquito bites by sleeping under insecticide-treated bed nets. Effective treatment with antimalarial drugs can also reduce malaria transmission.

**Why Was This Study Done?:**

One reason why malaria kills so many children in Africa is poverty. Many children in Africa are malnourished, and malnutrition—in particular, insufficient micronutrients in the diet—impairs the immune system, which increases the frequency and severity of many childhood diseases. Micronutrients are vitamins and minerals that everyone needs in small quantities for good health. Zinc is one of the micronutrients that helps to maintain a healthy immune system, but zinc deficiency is very common among African children. Zinc supplementation has been shown to reduce the burden of diarrhea in developing countries, so might it also reduce the burden of malaria? Unfortunately, the existing evidence is confusing—some trials show that zinc supplementation protects against malaria but others show no evidence of protection. One possibility for these conflicting results could be that zinc supplementation alone is not sufficient—supplementation with other micronutrients might be needed for zinc to have an effect. In this randomized trial (a study that compares the effects of different interventions in groups that initially are similar in all characteristics except for intervention), the researchers investigate the effect of supplementation with zinc alone and in combination with other micronutrients on the rate of uncomplicated (mild) malaria among children living in Tanzania.

**What Did the Researchers Do and Find?:**

The researchers enrolled 612 children aged 6–60 months who were living in a rural area of Tanzania with intense malaria transmission and randomly assigned them to receive daily oral supplements containing zinc alone, multi-nutrients (including iron) without zinc, multi-nutrients with zinc, or a placebo (no micronutrients). Nutritional indicators (including zinc concentrations in blood plasma) were assessed at baseline and 6–10 months after starting the intervention. During the study period, there were 1,572 malaria episodes. The incidence of malaria in all four intervention groups was very similar (about three episodes per child-year), and there was no evidence that multi-nutrients influenced the effect of zinc (or vice versa). Moreover, none of the supplements had any effect on malaria rates when compared to the placebo, even though the occurrence of zinc deficiency was strongly reduced by zinc supplementation. In a secondary analysis in which they analyzed their data by iron status at baseline, the researchers found that multi-nutrient supplementation increased the overall number of malaria episodes in children with iron deficiency by 41%, whereas multi-nutrient supplementation had no effect on the number of malaria episodes among children who were iron-replete at baseline.

**What Do These Findings Mean?:**

In this study, the researchers found no evidence that zinc supplementation protected against malaria among young children living in Tanzania when given alone or in combination with other multi-nutrients. However, the researchers did find some evidence that multi-nutrient supplementation may increase the risk of malaria in children with iron deficiency. Because this finding came out of a secondary analysis of the data, it needs to be confirmed in a trial specifically designed to assess the effect of multi-nutrient supplements on malaria risk in iron-deficient children. Nevertheless, it is a potentially worrying result because, on the basis of evidence from a single study, the World Health Organization currently recommends that regular iron supplements be given to iron-deficient children in settings where there is adequate access to anti-malarial treatment. This recommendation should be reconsidered, suggest the researchers, and the safety of multi-nutrient mixes that contain iron and that are dispensed in countries affected by malaria should also be carefully evaluated.

**Additional Information:**

Please access these Web sites via the online version of this summary at http://dx.doi.org/10.1371/journal.pmed.1001125.

- Information is available from the World Health Organization on malaria (in several languages), on micronutrients, and on zinc deficiency; the 2010 World Malaria Report provides details of the current global malaria situation
- The US Centers for Disease Control and Prevention provide information on malaria (in English and Spanish), including a selection of personal stories about malaria
- Information is available from the Roll Back Malaria Partnership on the global control of malaria and on malaria in Africa
- The Malaria Centre at the UK London School of Hygiene & Tropical Medicine develops tools, techniques, and knowledge about malaria, and has a strong emphasis on teaching, training, and translating research outcomes into practice
- The Micronutrient Initiative, the Global Alliance for Improved Nutrition, and the Flour Fortification Initiative are not-for-profit organizations dedicated to ensuring that people in developing countries get the minerals and vitamins they need to survive and thrive
- The International Zinc Nutrition Consultative Group (iZiNCG) is a non-profit organization that aims to promote and assist efforts to reduce zinc deficiency worldwide, through advocacy efforts, education, and technical assistance
- MedlinePlus provides links to additional information on malaria (in English and Spanish)

## Introduction

Preventive zinc supplementation among children in low-income countries can reduce the burden of diarrhoea and respiratory tract infections [1]. If effective against malaria, it would be an important advance in public health, particularly in Africa, where 90% of malarial deaths occur. Anti-malarial efficacy of zinc supplementation has thus far been investigated in four trials of which we are aware, with discordant results. In one trial in Gambian children, twice-weekly zinc seemed to reduce the mean number of clinic visits for malaria by 32% [2], but this trial was not designed to evaluate protection from malaria, and the statistical evidence was weak. In another trial in Papua New Guinean children, daily zinc reduced rates of malaria due to *Plasmodium falciparum* by 38% [3]. By contrast, in trials in Burkina Faso and Peru [4],[5], there was no evidence of protection against malaria in the children enrolled, despite zinc deficiency being highly prevalent at baseline and reversed by supplementation.

In these trials, malaria was detected among children self-reporting at clinics in Papua New Guinea and The Gambia, whereas cases were detected through regular home visits in Burkina Faso and Peru. This could indicate that zinc supplementation is more efficacious in preventing severe malaria than mild episodes, for which mothers do not immediately seek treatment. Alternatively, the response to zinc may be suppressed when children are also deficient in other nutrients [6],[7]. Simultaneous supplementation with other nutrients may be required to overcome a lack of effect of zinc supplements when given alone.

There are concerns, however, about the safety of supplementation with micronutrients in malaria-endemic areas. In a large trial among children aged 1–35 months in Pemba [8], supplementation with iron and folic acid increased the incidence of serious adverse events by 12%, which was assumed to be due to malaria. Results from a sub-study within this trial suggested that the overall effect tended to be beneficial in settings with improved basic care facilities. Also, the increase in adverse events appeared restricted to those who were iron-replete, whereas supplementation seemed beneficial in those who were iron-deficient [8],[9]. Based on these findings, the World Health Organization now advocates a policy of restricting iron supplementation to children with iron deficiency [10]. To strengthen the basis of this recommendation, the interaction with iron status and the safety of iron supplements in iron deficient children should be confirmed in other studies.

Although randomised trials in malaria-endemic areas have consistently shown that preventive iron supplementation (with or without other micronutrients) reduces the prevalence of anaemia [11]–[15], we have not found reports on its effect on haemoglobin concentrations during febrile episodes of malaria. By increasing parasite propagation during such episodes, supplementation may potentially exacerbate haemolysis. Thus, gains in haemoglobin concentrations due to supplementation before febrile malaria attacks may be more than compensated for by greater haemoglobin losses during such episodes. In a study in Papua New Guinean infants (aged 6–12 months), the decrease in haemoglobin concentration associated with asymptomatic *Plasmodium* infection at two surveys seemed more pronounced among those receiving iron [16]. By contrast, while supplementation with iron and folic acid increased rates of cerebral malaria in Pemba, it seemed to reduce rates of severe anaemia, although the statistical evidence for this reduction was not strong [8].

The primary aim of our study was to assess the effect of supplementation with zinc, alone or in combination with other nutrients, on rates of uncomplicated malaria. Secondary aims were to compare intervention effects in subgroups pre-defined by age, presence of parasitaemia, and zinc and iron status at baseline. We also assessed the effect of multi-nutrient supplementation on haemoglobin concentrations during febrile malaria episodes.

## Methods

### Study Population

We conducted the study between February 2008 and March 2009, in a rural area in Handeni District, Tanzania [17],[18] where malaria transmission is intense [19]. The trial protocol (Text S1) and the CONSORT checklist (Text S2) are available online as supporting information. The study (ClinicalTrials.gov: NCT00623857) was approved by the Ethical Review Committee of Wageningen University, The Netherlands and the National Health Research Ethics Review sub-Committee, Dar es Salaam, Tanzania. We sought written individual informed consent; parents or primary caretakers were invited to sign (or thumbprint if illiterate) the informed consent form witnessed by a member of the community (who countersigned the form). There were no important changes to methods during the trial.

### Study Design

In this trial with a parallel design, children were randomized to receive daily supplements with: (a) zinc; (b) both zinc and multi-nutrients; (c) multi-nutrients without zinc; or (d) placebo. Such a 2×2 factorial design allows the investigation of interaction between interventions; in the absence of interaction (i.e., the interventions act independently), data can be analysed ‘at the margins’ [20], and the design can ‘achieve two trials for the price of one’: every participant contributes information to each of the randomized factors simultaneously [21]. The multi-nutrient supplement included iron (18 mg as ferrous fumarate), folic acid (93.75 µg), vitamin A (450 µg, as retinyl palmitate), vitamin B_12_ (1.17 µg), vitamin B_1_ (0.625 mg as thiamine nitrate), vitamin B_2_ (0.55 mg riboflavin), copper (340 µg), and vitamin E (6.6 mg as RRR-α-tocopherol), all of which may have haematinic effects. For practical reasons, children participating in the study daily received one standard dose of supplements, regardless of age class. The amount of iron was set to supply on average 2 mg/kg body weight/day, as recommended to prevent iron deficiency anaemia [22]. For the youngest children (6–12 months), this amount was very close to the RNI under conditions of low bioavailability (18.6 mg). The average intakes of iron actually achieved were 2.3 mg/kg, 1.7 mg/kg, and 1.3 mg/kg bodyweight, for age classes 6–17, 18–35, and 36–59 months, respectively. Table S1 provides further details of the composition of the supplements.

### Sample Size Calculations

Our aim was to measure a malaria rate reduction by 30% unambiguously [2], and to exclude the null value from the 95%CI with a minimum probability of 80%. With an anticipated malaria incidence in the reference group of 1.05 episodes per child-year, this required an effective sample size of 142 child-years per group. It should be noted, however, that the relevance of sample size calculations to the interpretation of study findings is controversial [23],[24]; specifically, in our study, the incidence actually found in the placebo group was much higher than originally foreseen. No interim analyses were foreseen or done.

### Recruitment

In four villages, resident children aged 6–60 months were listed, screened, and enrolled in daily batches until attaining the target number (n = 600) in August 2008 (Figure S1). Anthropometric indices were computed as the average of two measurements taken on consecutive days. Following a physical examination, venous blood was collected in EDTA-tubes suitable for trace element analyses (Becton-Dickinson, Franklin Lakes, NJ, USA). One aliquot was centrifuged immediately after collection and plasma stored in liquid nitrogen; a second aliquot was examined by haematology analyser (Sysmex KX21, Kobe, Japan).

Malaria dipstick tests (CareStart, G0121, Access Bio, Monmouth Jct, NJ) were used to detect lactate dehydrogenase produced by live *P. falciparum* or other human *Plasmodium* species. This test has a sensitivity of 96% for samples with >50 *P. falciparum* parasites/mL, and detects only current parasitaemia [25]. Blood films were prepared for all children. All children with current *Plasmodium* infection, as indicated by a positive result for a dipstick test, received a therapeutic course of artemether-lumefantrine upon enrolment so that they were at risk of malaria after a post-treatment prophylactic period [26] in which lumefantrine levels exceeded the minimum concentration inhibiting parasite multiplication.

Children with height-for-age z-scores>−1.5 SD (who are at lower risk of zinc deficiency) [27], weight-for-height z-score<−3 SD, haemoglobin concentration <70 g/L, those unlikely to comply with interventions, whose parents/guardians refused consent, or with signs of severe or chronic disease, were excluded from participation.

### Randomisation and Masking

We used stratified block randomisation to allocate interventions. A colleague not otherwise involved in the trial used tables with random numbers to generate the allocation sequence consisting of randomly permuted blocks with random size (4 or 8) within each of six strata defined by *Plasmodium* infection (yes/no infected) and age class (6–17 months, 18–35 months, and 36–60 months). Interventions were indicated by colour code on paper slips in opaque, consecutively numbered envelopes that were prepared in advance, in excess of the expected number required. A simple colour code (one colour to distinguish each of the four supplement types) was used to minimize the possibility of children receiving the wrong supplements. This code was not revealed to researchers, field staff, or participants, who therefore did not know who received what intervention. At the end of each screening day, the names of eligible children were listed by screening number and each name was randomly allocated to an intervention by drawing the next envelope from a box that corresponded to the age- and malaria-specific stratum for that child.

Supplements, as powder in colour-coded capsules, were contained in blister packs, and administered orally after suspending capsule contents in clean water or breast milk. All types of powder had similar appearance, smell, and taste. At the end of each screening day, when eligibility had been fully established, children were individually allocated in order of their screening number to intervention groups by drawing successive envelopes from a box corresponding to the infection- and age-specific stratum for that child. The number of the envelope was then recorded on a list before the envelope was opened. The randomisation code was not revealed to researchers, field workers, or participants until data collection was completed and the database had been finalised and sent to the Trial Oversight Committee. The colour of the supplements received by each child was known to participants and field workers but not by the clinical outcome assessors.

### Follow-up and Case Detection

**Figure 1:**
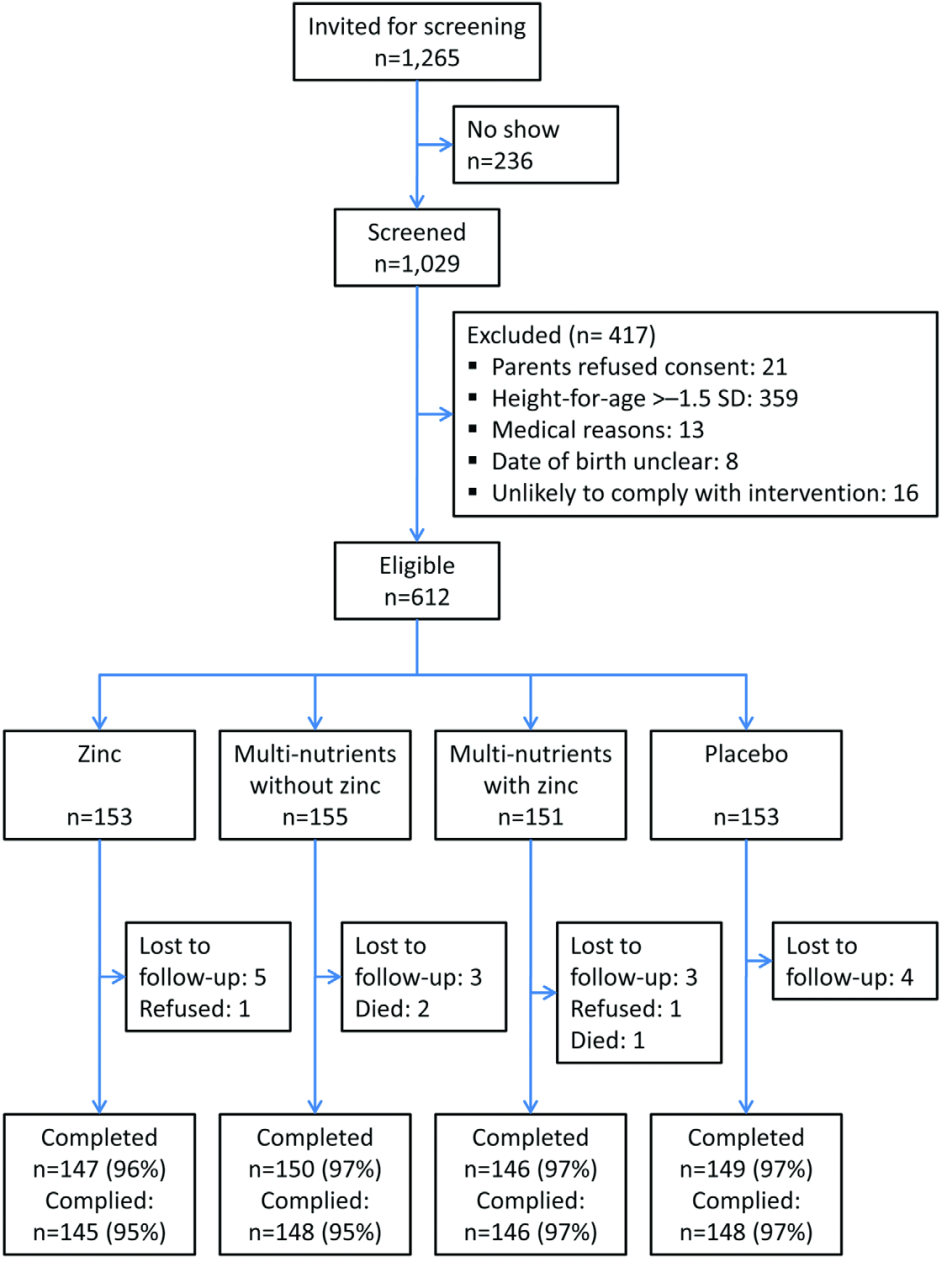
Flow chart of study recruitment and follow-up. Compliance was measured as the proportion of children who consumed >95% of scheduled supplements.

Community volunteers administered supplements 7 days per week close to the homes of participating children and reported daily to field staff, who followed up the same day in cases of non-compliance. Field staff made regular, unannounced spot checks to ensure adherence to procedures. Supplementation and follow-up continued for all children until 12 March 2009, when the trial was stopped (Figure 1). Because we could not start the study on the date originally foreseen, we had to stop the trial when running out of resources, before the planned number of person-years were accrued, but after the desired number of events had been accrued.

Parents were requested to bring study children to the clinic if their child developed a fever or became unwell. A clinical officer was on 24-hour duty and collected medical information on standardised forms. Axillary temperature was measured using an electronic thermometer and dipstick tests administered for children with guardian-reported fever; for those with positive test results, we prepared two blood films and measured whole-blood C-reactive protein concentrations using a point-of-care test (QuikRead, Orion Diagnostica, Espoo, Finland).

In accordance with national guidelines, we treated uncomplicated malaria with artemether-lumefantrine (Novartis Pharma, Basel, Switzerland). This drug combination is highly efficacious [28],[29], and was available free of charge at government health-facilities but not in local shops. Participating children received free medical care for common illnesses. Because of the strategic location of the research clinic, and based on interviews with local informants, we believe that very few sick participants were brought to other health facilities or were treated at home.

A second survey, at 251 days (median; 95% reference range: 191–296 days) after enrolment, followed similar procedures.

### Laboratory Analyses

Peripheral blood parasite density was determined by microscopy; slides with results that were inconsistent with those from the dipstick test were read twice. Asexual *Plasmodium* parasites were counted against at least 200 leukocytes, and density, expressed per µL of blood, was estimated using an assumed leukocyte density of 8,000/µL. For children with very high densities, parasites were counted per 2,000 erythrocytes, in which case we used the estimated erythrocyte count at the time of the episode to determine the number of parasites per µL. The erythrocyte density was estimated based on haemoglobin concentration measured by HemoCue meter, using a linear model describing the relationship between haemoglobin concentrations and erythrocyte counts as assessed during surveys. Plasma concentrations of C-reactive protein and ferritin were measured (Meander Medical Centre, Amersfoort, The Netherlands) on a Beckman Coulter Unicel DxC880i system according to the manufacturer's instructions. Plasma zinc concentrations were determined by inductively-coupled plasma-mass spectrometry (Varian 820-MS; CV: 9% at 26.8 µM; 13% at 21.25 µM and 13% at 15 µM; n = 32, V = 10 µL).

### Statistical Analysis

Data were analysed following a pre-specified plan, by intention-to-treat, using SPSS (v15·0 for Windows, SPSS, Chicago, IL, USA), CIA (v2.1.2) [30] and STATA (v11; College Station, TX, USA). Compliance was measured as the proportion of children who consumed >95% of scheduled supplements. Nutritional status was defined by the presence of iron deficiency (plasma ferritin concentration <12 µg/L) [22], zinc deficiency (plasma zinc concentration <9.9 µmol/mL) [31] or being stunted (height-for-age z-score<−2 SD).

The primary outcome, an episode of malaria, was pre-defined as a positive result for the malaria dipstick test in children with guardian-reported fever in the previous 24 hours and either: (a) confirmed fever (axillary temperature ≥37.5°C), or (b) unconfirmed fever with inflammation (whole blood C-reactive protein concentrations ≥8 mg/L), separated by at least 14 days from a previous malaria episode. It has been recommended that only measured fever should be used to identify malaria cases, and to exclude cases of unconfirmed fever from the analysis [32]. We considered this approach would miss many malaria episodes because temperature can fluctuate strongly over the day and many fever cases would remain undetected during the relatively short visit to the health facility. Thus we included inflammation as additional criterion in the case definition for cases of unconfirmed fever. In the primary analysis we did not use a parasite density threshold in the malaria case definition [33],[34], because this can lead to biased estimates of intervention effects when the interventions affect parasite density [35],[36]; in addition, density estimates can vary greatly within short time spans, and ideally require leukocyte counts to be determined simultaneously [35],[37],[38]. To increase the specificity of malaria case definitions, *Plasmodium*-infected participants were treated at baseline to clear parasitaemia before the start of surveillance. Episodes with pre-defined parasitaemia thresholds (1,000, 3,000, and 5,000 asexual parasites/µL) were considered as secondary outcomes. We also assessed the effect of the intervention on relatively severe episodes (with parasite densities exceeding 10,000 or 100,000 parasites/µL).

Because we considered *a priori* a reduction in overall malaria disease burden of primary public health importance [39],[40], our primary analysis included all malaria events. We used Cox models with robust estimates of the standard error to account for correlation between episodes within children and interpreted the hazard ratio as a proxy for the incidence ratio. We calculated the percentage reduction (or increase) due to the intervention as 100×(1−hazard ratio). Following the analysis plan, we adjusted for prognostic factors at baseline (age class [6–18 months, 18–35 months, and 36–59 months], *Plasmodium* infection, mosquito net use, distance between homestead and clinic, height-for-age z-score). We evaluated possible interaction between zinc and multi-nutrients by including an interaction term in the Cox regression model. We also conducted a pre-specified secondary analysis to assess the influence on effect estimates of excluding observations in a 14-day post-treatment prophylactic period. To assess changes in intervention effect over time, we explored effects on all malaria episodes within the first 100 days of supplementation versus the subsequent period. We arbitrarily defined a cut-point of 100 days because this period covered almost half of all episodes, and adjusted for baseline factors as described above. We similarly explored intervention effects within the first 50 days.

In a secondary analysis we assessed intervention effects on time-to-first malaria episode using Kaplan-Meier analysis, and compared hazard rates of first episodes using Cox regression.

After we concluded that there was no evidence for interaction between zinc and multi-nutrients on malaria rates, we conducted pre-specified subgroup analyses (all events; unadjusted) to explore to what extent the magnitude of marginal intervention effects on malaria frequency depended on age class, presence of parasitaemia, and zinc and iron status at baseline, by including (for each factor in turn) interaction terms in the Cox regression models. Lastly, we explored whether differences in intervention effects between subgroups were consistent when using higher parasite density cut-offs.

## Results

Of 1,029 screened children, 662 had height-for-age z-scores≤−1.5 SD; of these, 612 were eligible and randomised. Twenty children (3%) did not complete the trial: three died, two were withdrawn by parents, and 15 emigrated from the area (Figure 1). Another two children discontinued the intervention but were available for follow-up. Compliance was high (96%) and similar in all four groups.

Groups were similar in baseline characteristics except that there were slightly more boys and zinc-deficient children in the multi-nutrient group (Table 1). The prevalence of zinc deficiency was 67% overall, and 60% in those without inflammation; the prevalence of zinc deficiency was dramatically reduced by zinc supplementation, whether given alone or with other micronutrients (Table 2).

**Table 1:** Baseline characteristics of study participants, by intervention group.

	Zinc	Multi-nutrients without Zinc	Multi-nutrients with Zinc	Placebo
**n**	153	155	151	153
**Sex M/F [n/n]**	46%/54% [70/83]	56%/44% [87/68]	44%/56% [66/85]	50%/50% [76/77]
**Age class**				
6–17 months	24% [36]	23% [36]	24% [36]	24% [36]
18–35 months	36% [55]	36% [55]	34% [51]	35% [54]
36–59 months	41% [62]	41% [64]	42% [64]	41% [63]
***Plasmodium*** ** infection** a	43% [66]	41% [64]	44% [67]	44% [68]
**Height-for-age, z-score**	−2·36±0·69	−2·50±0·69	−2·39±0·71	−2·45±0·69
**Inflammation** b	34% [52]	33% [51]	34% [51]	31% [47]
**Zinc deficiency** c				
All children	63% [97]	71% [110]	70% [105]	65% [100]
Without inflammation^d^	58% [59]	65% [68]	59% [59]	60% [64]
**Haemoglobin concentration, g/L**	101·8±12·6	102·7±12·8	103·8±12·7	102.8±12·7
**Anaemia** e	75% [114]	65% [100]	68% [103]	65% [100]
**Iron deficiency** f				
All children	16% [25]	18% [28]	20% [30]	19% [28]
Without inflammation^d^	23% [23]	24% [25]	24% [24]	24% [25]
**Iron deficiency anaemia**				
All children	12% [18]	12% [19]	15% [23]	14% [21]
Without inflammation^d^	16% [16]	16% [17]	18% [18]	18% [19]
**Distance from homestead to dispensary, km** g	3·66±2·31	3·52±2·06	3·54±2·07	3·60±3·38
**Mosquito net use** h	32% [48]	36% [55]	30% [45]	31% [46]

Mean ± SD, % [n] or median (25- and 75-percentiles) unless indicated otherwise.

a As indicated by a positive result for pLDH-based dipstick test (see text).

b Plasma C-reactive protein concentration ≥8 mg/L.

c Plasma zinc concentration <9.9 µmol/L.

d n = 101, 104, 100 and 106, respectively (five missing values for plasma ferritin concentration).

e Haemoglobin concentration <110 g/L.

f Plasma ferritin concentration <12 µg/L (six missing values).

g Measured as the crow flies, based on global positioning data.

h Data missing for 11 children.

**Table 2:** Intervention effects on indicators of nutritional status, inflammation and malaria at the second survey.

	Zinc				Micronutrients without Zinc				Micronutrients with Zinc				Placebo	
	Estimate		Effect^a^		Estimate		Effect^a^		Estimate		Effect^a^		Estimate	
**n** b	149				151				148				150	
***Plasmodium*** ** infection** c	33%	[50]	5%	(−5% to 15%)	34%	[52]	6%	(−4% to 16%)	33%	[49]	5%	(−5% to 15%)	28%	[42]
**Inflammation** d	35%	[54]	2%	(−9% to 12%)	25%	[38]	−10%	(−20% to 1%)	33%	[50]	−1%	(−12% to 10%)	34%	[52]
**Plasma zinc concentration, µmol/L**	16.0	[6.2]	6.4	(5·4 to 7·3)	9.6	[2.6]	0.0	(−0.9 to 0.9)	13.5	[4.2]	3.9	(2.9 to 4.8)	9.6	[2.7]
**Zinc deficiency** e														
All children	11%	[16]	−52%	(−60% to −42%)	57%	[89]	−5%	(−15% to 6%)	22%	[33]	−40%	(−50% to −30%)	62%	[95]
Without inflammation	4%	[4]	−58%	(−67% to −46%)	47%	[53]	−15%	(−27% to −1%)	20%	[19]	−43%	(−54% to −29%)	62%	[60]
**Haemoglobin concentration, g/L**	103.7	[11.8]	−0.4	(−3·0 to 2·3)	106.6	[10.7]	2.6	(0·0 to 5·2)	107.5	[11.4]	3.5	(0·8 to 6·1)	104.0	[11.9]
**Anaemia** f	65%	[100]	1%	(−10% to 11%)	50%	[77]	−15%	(−26 to −4%)	52%	[79]	−12%	(−23% to −1%)	65%	[99]
**Plasma ferritin concentration** g														
All children	31.1	[0.03]	−1.6	(−6·8 to 4·8)	57.1	[0.03]	24.5	(14·8 to 36·2)	57.2	[0.03]	24.6	(14·8 to 36·3)	32.6	[0.03]
Without inflammation	23.2	[0.04]	−1.2	(−5·5 to 4·2)	43.9	[0.03]	19.5	(11·3 to 28·6)	51.1	[0.03]	26.7	(17·5 to 38·1)	24.4	[0.04]
**Iron deficiency** h														
All children	11%	[17]	−2%	(−9% to 6%)	1%	[1]	−12%	(−19% to −7%)		[0]	−13%	(−19% to −8%)	13%	[20]
Without inflammation	17%	[16/95]	−3%	(−14% to 8%)	1%	[1/113]	−20%	(−29% to −12%)		[0/98]	−20%	(−29% to −12%)	20%	[19/97]

Effects in the three groups receiving zinc alone, multi-nutrients without zinc and zinc plus multi-nutrients, as compared to placebo. Estimates indicate prevalence values [n], arithmetic mean [SD], or geometric mean [SE].

a Difference relative to placebo (95%CI), for prevalences computed using Newcombe's method [30].

b Differences between numbers reported and numbers randomised are due to drop-outs; percentages are computed with the number of randomised children in the denominator.

c As indicated by a positive result for pLDH-based dipstick test (see text).

d Plasma C-reactive protein concentration ≥8 mg/L.

e Plasma zinc concentration <9.9 µmol/L.

f Haemoglobin concentration <110 g/L.

g Geometric mean.

h Plasma ferritin concentration <12 µg/L [22].

There were 3,268 clinic visits during the study period, of which 2,462 (75%) were accompanied by guardian-reported fever. Of 2,462 episodes of reported fever, 1,618 (66%) were accompanied by *Plasmodium* infection as detected by dipstick test; for 46 children parasitaemia was not accompanied by confirmed fever or inflammation. Hence, 1,572 episodes classified as malaria: 1,499 (95%) due to *P. falciparum* and 73 (5%) due to other *Plasmodium* species. The incidence of malaria was 3.0 events/child-year. There were 1,408, 1,314, 1,248, 1,119, and 263 episodes with densities exceeding 1,000, 3,000, 5,000, 10,000, and 100,000 parasites/µL, respectively. Of all hospital admissions (68), almost half (30 cases occurring in 27 children) were due to malaria: five were admitted for life-threatening disease (respiratory distress or prostration; none had neurological manifestations); 25 lived far from the dispensary, and the clinician referred them as a precaution (18 for haemoglobin concentrations <60 g/L without respiratory distress or dehydration and seven for other reasons). There were no episodes of cerebral malaria.

### Primary Analysis: Effects on All Episodes of Malaria

Compared to placebo, there was no evidence of a meaningful effect of either zinc or multi-nutrients alone on the incidence of malaria. The hazard ratio for all episodes of malaria by zinc was 0.99; 95% CI 0.82–1.18 (adjusted model, as compared to placebo). Thus for zinc, the lower limit of the confidence interval is compatible with a reduction in incidence of all episodes of malaria of 18% only. The hazard ratio for all episodes of malaria by multi-nutrients (as compared to placebo) was 1.04, 95% CI 0.87–1.23 (adjusted model; see Table 3). Incidence of all episodes of malaria was slightly higher in the group that received both zinc and multi-nutrients, as compared to placebo (HR 1.14, 95% CI 0.96–1.35), but there was no evidence of an interaction between zinc and multi-nutrients in their effects on malaria incidence (interaction ratio 1.10, 95% CI 0.84–1.43). In the remainder of this paper, we will assume that the interventions acted independently, and report marginal analyses accordingly [20]. Thus analysed, there was no evidence that zinc supplementation affected malaria rates (adjusted HR: 1.04, 95% CI 0.93–1.18), whereas supplementation with multi-nutrients seemed to slightly increase malaria rates (adjusted HR 1.10, 95% CI 0.97–1.24). Excluding observations for 14 days post-treatment or restricting the analysis to *P. falciparum* cases did not change our conclusions (unpublished data).

**Table 3:** Incidence rates by intervention group and intervention effects on malaria rates, relative to placebo.

Event	Zinc		Multi-nutrients without Zinc		Multi-nutrients with Zinc		Placebo	
**All episodes of malaria** (1,572 cases)								
Incidence	2.89	[378/130.8]	2.95	[393/133.2]	3.26	[423/129.7]	2.87	[378/131.9]
HR, crude	1.01	(0.87 to 1.16)	1.03	(0.89 to 1.19)	1.14	(0.99 to 1.31)	1.0	(Reference)
HR, adjusted^a^	0.99	[0.82 to 1.18]	1.04	[0.87 to 1.23]	1.14	[0.96 to 1.35]	1.0	(Reference)
**First episode of malaria** (507 cases)								
Incidence	2.71	[124/45.8]	3.27	[133/40.7]	3.37	[129/38.3]	2.52	[121/47.9]
Incidence ratio	1.08	(0.83 to 1.38)	1.29	(1.01 to 1.65)	1.33	(1.04 to 1.71)	1.0	(Reference)
HR, adjusted^a^	1.12	(0.86 to 1.44)	1.35	(1.05 to 1.73)	1.38	(1.07 to 1.77)	1.0	(Reference)

Numbers in brackets indicate [no. events/no. person-year] or (95% CIs).

a Hazard ratio (HR), adjusted for age class (18–35 months and 36–59 months), presence of *Plasmodium* infection, mosquito net use (binary variable), distance between homestead and clinic (continuous variable), and height-for-age z-score (continuous variable) at baseline.

### Secondary Analyses: Effects of Zinc on Malaria Rates

**Figure 2:**
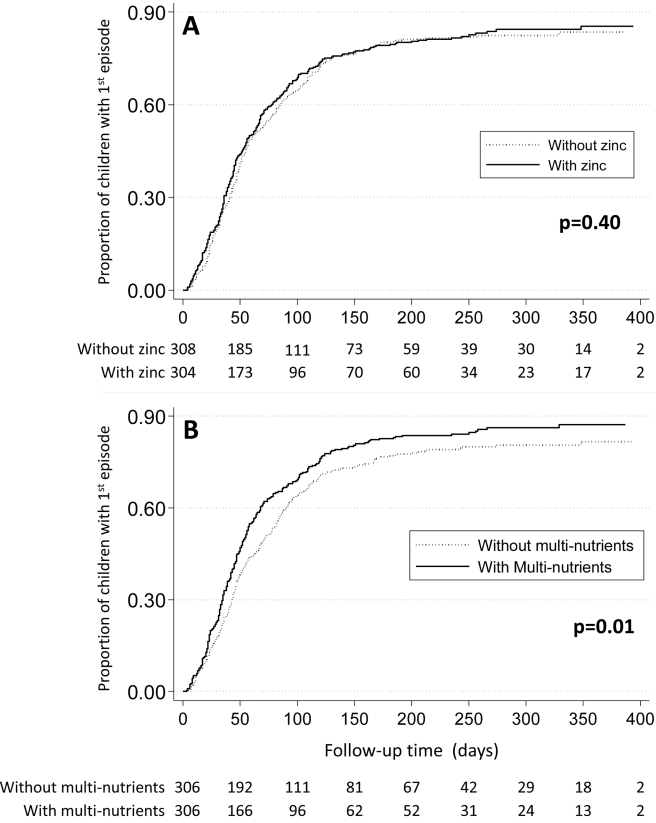
Effect of zinc (panel A) or multi-nutrients (panel B) on time to first malaria episode. Marginal group comparisons (Kaplan-Meier analysis), with p-values obtained by Tarone-Ware test. Values below each panel indicates the number of children at risk.

Likewise, when using different case definitions, we found no evidence that zinc protected against malaria when episodes were defined using density thresholds of 1,000 parasites/µL (HR 1.02, 95% CI 0.90–1.16), 3,000 parasites/µL (HR 1.01, 95% CI 0.88–1.17) or 5,000 parasites/µL (HR 1.00, 95% CI 0.87–1.16), or that zinc influenced the incidence or time-to-first episode of malaria (Table 3; Figure 2). Nor was there evidence that rates of episodes with parasite densities exceeding 10,000 parasites/µL and 100,000 parasites/µL were reduced by zinc (HR 1.00, 95% CI 0.87–1.16 and HR 1.19, 95% CI 0.89–1.58, respectively).

Lastly, we found no evidence that zinc protected against malaria after 100 days of supplementation (HR 1.01, 95% CI 0.86–1.18), or that the intervention effect depended on the duration of supplementation (100 days or less versus more than 100 days, p = 0.29), or by any of the pre-specified baseline factors (age class, *Plasmodium* infection, height-for-age z-score or zinc status) or by sex (unpublished data).

### Secondary Analyses: Effects of Multi-nutrients on Malaria Rates

There was some indication that the effect of multi-nutrients on malaria rates decreased over time: when analysis was limited to the first 100 days of supplementation, the hazard ratio was 1.17 (95% CI 1.02–1.34), and in the first 50 days 1.23 (95% CI 1.02–1.50), but the statistical evidence for a change in the effect of multi-nutrients over time was weak (p-value for the change in effect 0.10 and 0.17 with time entered as continuous or categorical [< or ≥100 days] variable). When analysis was limited to the first or only episode, malaria rates were greater in those who received multi-nutrients (HR 1.29, 95% CI 1.08–1.54), and the median time to the first episode of malaria was 54 days for children receiving multi-nutrients, compared to 72 days for those without (p = 0.01; Tarone-Ware test, Figure 2).

**Figure 3:**
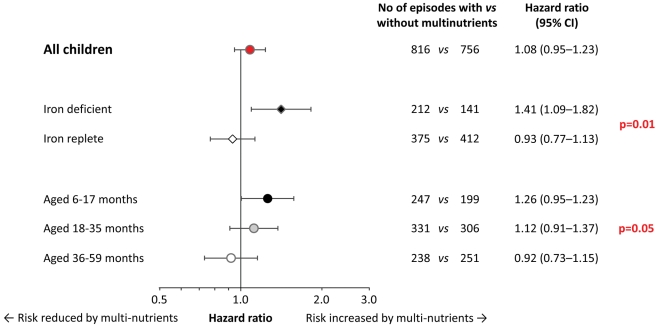
Effect of multi-nutrient supplementation on malaria rates, by iron status and age class. Malaria with case definition as pre-defined in the analysis plan. Values on the right indicate crude hazard ratios (95% CIs); p-values for differences in intervention effects between subgroups (with age class entered on an ordinal scale). Initial iron status was defined as iron-deficient (black diamond; plasma ferritin concentration <12 µg/L, n = 111) or iron replete (white diamond; plasma ferritin concentration ≥12 µg/L, without inflammation; n = 312). In the analysis, we excluded children in whom iron status was uncertain (plasma ferritin concentration ≥12 µg/L, with inflammation); hence analyses are restricted to 423 subjects. Adjustment for distance between homestead and dispensary, height-for-age z-scores, mosquito net use, and *Plasmodium* infection at baseline led to similar estimates (unpublished data).

When analysing the data by iron status at baseline, multi-nutrient supplementation appeared to increase the overall number of malaria episodes by 41% (95% CI 9%–82%) in children with iron deficiency, whereas there was no evident effect in their iron-replete peers (Figure 3; p-value for difference in effect 0.01). For episodes with densities >10,000 and >100,000 parasites/µL, similar effect modification was observed, whereby the increase in rates remained consistently and, with similar magnitude, restricted to children with iron deficiency (Figure S2).

The prevalence of iron deficiency at baseline decreased rapidly with age (38%, 20% and 5% in children aged 6–17 months, 18–35 months, and 36–60 months, respectively; p<0.001; corresponding values in those without inflammation: 45%, 28%, and 7%; p<0.001). As with initial iron status, age strongly modified the effect of multi-nutrients on malaria incidence (Figure 3). Contrasts between age-specific effects on malaria rates increased when defining episodes with higher parasite density cut-offs (Figure S3). For episodes with hyper-parasitaemia (>100,000 parasites/µL), multi-nutrients increased rates by 54% (95% CI 1%–137%) in the youngest children, but reduced rates by 58% (95% CI −3% to −82%) in the eldest (p-value for interaction: 0.006), with an overall neutral effect.

### Secondary Analyses: Effect of Multi-nutrients on Haemoglobin Concentrations

**Figure 4:**
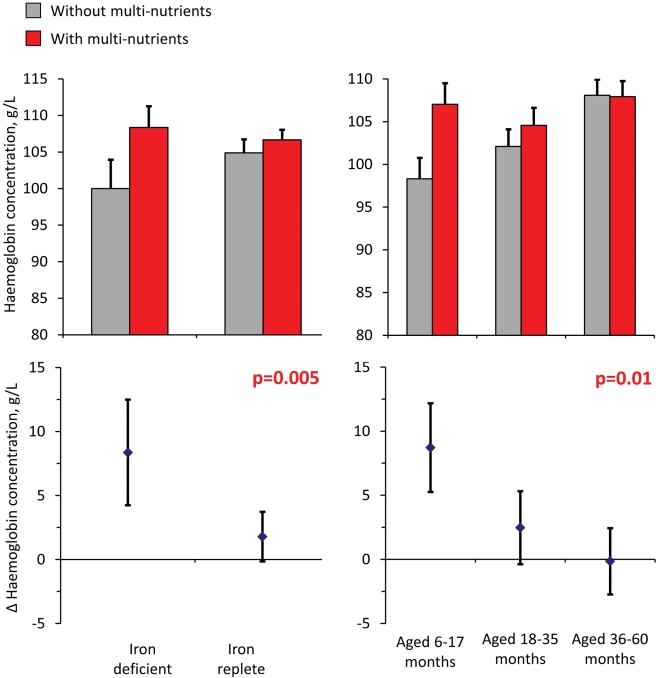
Effect of multi-nutrients on haemoglobin concentration at the second survey, by iron status and age class. Top: haemoglobin concentrations, by supplementation group. Bottom: effects of multi-nutrients on haemoglobin concentrations. Left: By initial iron status. Right: By age class. Line bars indicate 95% CIs (only upper half of the interval indicated in top panel). Analysis based on 598 children. All estimates are adjusted for standardized haemoglobin concentrations at baseline. p-Values indicate interaction between age class or iron status and intervention effects on haemoglobin concentration.

**Figure 5:**
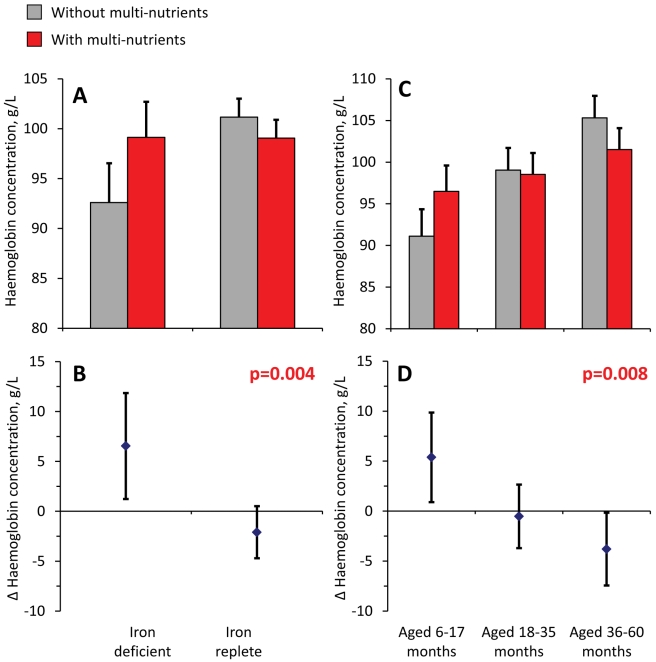
Effect of multi-nutrients on haemoglobin concentration during the first malaria episode, by iron status and age class. Top: haemoglobin concentrations, by supplementation group. Bottom: effects of multi-nutrients on haemoglobin concentrations. (A and B) By initial iron status. C and D) By age class. Line bars indicate 95% CIs (only upper half of the interval indicated in top panel). Analysis is based on 507 episodes. All estimates are adjusted for standardized haemoglobin concentrations at baseline. Further adjustment for time between start of intervention and episode led to virtually identical estimates. p-Values indicate interaction between age class or iron status and intervention effects on haemoglobin concentration.

Analysis of haemoglobin concentrations at the second survey (when virtually all children were symptom-free) showed that the youngest children and those with iron deficiency at baseline responded most to multi-nutrient supplementation (8.4 g/L, 95% CI 4.2 −12.5 g/L and 8.7 g/L, 95% CI 5.3–12.2 g/L, respectively; Figure 4). Similar beneficial effects were found at time of the first malaria episode: multi-nutrients increased haemoglobin concentrations by 6.5 g/L (95% CI 1.2–11.9 g/L) in children with iron deficiency, while no marked effect was apparent in those who were iron replete (−2.1 g/L, 95% CI −4.7 to 0.5 g/L; difference in effect: 8.6 g/L, 95% CI 2.7–14.6 g/L; p-value 0.004); (Figure 5). Similar patterns were observed for the intervention effects on haemoglobin concentrations during the second malaria episode (unpublished data).

## Discussion

Despite a high prevalence of zinc deficiency, excellent compliance, and few drop-outs, we found no evidence from this trial that preventive zinc supplementation, alone or with multi-nutrients, reduced rates of febrile attacks of malaria. In the primary analysis, there was no evidence that multi-nutrients influenced malaria rates, but results from a secondary analysis suggest that multi-nutrient supplementation may have increased the incidence of first malaria episodes by approximately 30%. The effect of supplementation on overall malaria rates seemed to vary with iron status at baseline: among children with iron deficiency, multi-nutrients increased rates of malaria episodes by 41%, while they had no effect in those who were iron replete. This difference in effect was unlikely to have been observed by chance (p = 0.01).

One limitation of our study is that supplements were colour-coded and not in packs labelled with individual identity numbers. During the field work, however, we received no indication that participants or field staff discovered who received what intervention, or that participants favoured a particular type of supplement. It should be noted also that the clinical outcome assessors were blinded to what intervention had been assigned to individual children. HIV infection was not assessed in our study but is unlikely to have confounded its results: the prevalence of HIV infection in this paediatric population is probably below 5% and, due to the randomisation process, marked imbalances in HIV infection at baseline are unlikely.

By excluding children with height-for-age z-score>−1.5 SD, the children enrolled in our trial probably were more zinc deficient than the general population from which they were sampled. Thus the effect on malaria rates that could potentially be attained in the general population, without this exclusion criterion, is probably smaller than that reported here. Two previous trials indicated that zinc can protect against malaria. The Gambian trial [2] was not designed to assess this association, and episodes were recorded through routine clinical procedures. In Papua New Guinea, zinc reduced the incidence of febrile episodes with parasitaemia ≥9,200/µL and ≥100,000/µL by 38% (95% CI 3%–60%) and 69% (95% CI 36%–87%), respectively [3]. Both studies were smaller than our study (91 cases of *P. falciparum* malaria in Papua New Guinea, as compared to 1,499 in our study). It seems unlikely that discrepancies with our findings are explained by differences in access to health care and a differential effect on rates of severe malaria: even with episodes with parasitaemia ≥100,000/µL, our confidence intervals (Table 3) are incompatible with the magnitude of the protective effect reported from Papua New Guinea. In addition, the overall and malaria-specific mortality reduction found in Pemba (7% and 10%, respectively) [41], seems inconsistent with the 69% reduction in episodes with parasitaemia ≥100,000/µL that was reported from Papua New Guinea. The intensity of malaria transmission in Papua New Guinea was much lower than in Burkina Faso, Pemba, or our study, raising the question of whether zinc affords protection only in populations with low acquired immunity. The absence of an age-dependent decrease in efficacy of zinc supplementation in our study makes this unlikely. Thus the divergent results from Papua New Guinea may be due to unknown differences in host, parasite, or environmental factors that predict the response to zinc.

Because malaria parasites are transmitted through mosquito bites, they bypass epithelial barriers that form the first line of defence against pathogens that cause diarrhoea and respiratory infections. Zinc is known to play a critical role in maintaining epithelial barrier function [42]–[46]. In vitro studies using Caco-2 cells suggest that tight junctions between intestinal epithelial cells have impaired function in zinc deficiency [46], whilst morphological studies and findings from a zinc supplementation trial in Bangladeshi children with diarrhoea suggest that zinc deficiency adversely affects intestinal permeability [47],[48]. The protective effect of zinc against diarrhoea and respiratory infections, as compared to the absence of such an effect against malaria, suggest that this nutrient primarily acts by strengthening barrier function rather than through immunity.

Our results also suggest that an increase in overall malaria rates may have occurred in children with iron deficiency. Subgroup analyses should generally be interpreted with caution [49]. On one hand, we did not specify interactions between multi-nutrient supplementation and iron status *a priori*, and the direction of the subgroup effect is in contrast with the findings previously obtained in the Pemba sub-study with iron and folic acid [8]. On the other hand, the difference in effects of multi-nutrients between children with and without iron deficiency was large, the statistical evidence for effect modification was strong and consistent for other malaria-related outcomes (haemoglobin concentration and malaria episodes with higher parasite densities).

Because the effect of multi-nutrient supplementation on malaria in our trial depended on initial iron status, it would seem most likely that this was caused by iron, and not by other nutrients in the supplement. Diverse lines of evidence suggest that, of all the micronutrients, iron is the most critical mediator of host–pathogen interactions [50], and our findings build on existing evidence suggesting that supplementation with iron can increase malaria risk [8],[51]–[54].

In their report of the findings from the Pemba sub-study [8], the authors were cautious in their interpretation of the subgroup effect, stating that their results suggested that supplementation with iron and folic acid is beneficial in children with iron deficiency, but unsafe in those who are iron-replete. Based on these findings, an expert group convened by the World Health Organization recommended that iron supplements should be administered routinely to iron-deficient infants in settings with adequate access to anti-malarial treatment [10]. Unfortunately, our findings contradict those from the Pemba study, and thereby cast doubt on the assumption that iron is safe in iron-deficient children. In the Pemba study, iron deficiency (found in 75% of children) was defined by elevated molar ratios of zinc protoporphyrin and haem. Although to their credit, ZPP∶H ratios react more slowly to inflammation than plasma ferritin concentrations, elevated ratios may occur due to (longstanding) inflammation, (asymptomatic) *Plasmodium* infection, and thalassaemia (Veenemans, unpublished data), and hence falsely indicate iron deficiency. Plasma ferritin concentration below 12 µg/L as indicator of iron deficiency may fail to detect deficient children in the presence of inflammation, but it is a highly specific marker of iron deficiency, probably more so than the ZPP∶H ratio.

We speculate that iron may have enhanced parasite proliferation specifically in children with iron deficiency, because iron absorption in this subgroup is more efficient and thus may lead to transient production of non-transferrin bound iron [55]–[57], which may act as a nutritional source and favour the proliferation of *Plasmodium* parasites [10]. Evidence in support of this hypothesised mechanism is, however, lacking. In addition, children with a low plasma ferritin concentration are probably free of inflammation and likely to absorb iron rapidly. Because children with iron deficiency were substantially younger, the difference in effect between subgroups of iron-replete and -deficient children may also (partly) have been mediated by the lack of protective immunity in these younger children, and the higher dose of supplemental iron per kilogram of bodyweight. It seems plausible that the magnitude of potential adverse effects of iron is determined by a combination of the host's immune status, dose of iron per kg bodyweight, and the amount of iron absorbed. The qualitative interaction with age and iron status as observed in our study emphasizes the need for trials to take these into account in the analysis, as overall estimates may tend towards a neutral effect [58].

We found no support for our initial hypothesis that, at the time of malaria episodes, haemoglobin concentrations would be lower in children receiving multi-nutrients. In fact, young children with iron deficiency (who were most vulnerable to declines in haemoglobin) were better able to maintain their haemoglobin concentration when they received multi-nutrients. The critical issue remains whether the benefits of higher haemoglobin concentrations during acute malaria and thus the expected reduced risk of severe malarial anaemia, on one hand, outweigh the potential risk of severe disease manifestations that can be associated with an increased frequency of malaria, on the other hand. Access to primary care facilities may be a critical factor determining this balance, and in the absence of early treatment, the youngest children with iron deficiency may be a vulnerable subgroup.

The estimated amount of iron absorbed from our supplement is close to that absorbed from multi-nutrient mixes used for home fortification [59]–[61]. Our data question the safety of supplying these mixes to vulnerable populations, particularly in settings with poor malaria prevention, but even in conditions where access to diagnosis and therapy for malaria is excellent, as in our study.

In conclusion, when results from all trials are considered together, there is no evidence that zinc interventions can reduce the burden of malaria in African children. We have presented evidence that multi-nutrient supplementation may increase the risk of malaria in children with iron deficiency, strengthening earlier concerns about the safety of multi-nutrient supplementation in malaria-endemic areas, even in settings with good access to health care and appropriate treatment. Similar risks may apply to home fortification in such settings. Although safety may be improved by modifying the formulation of supplements (in particular by reducing the iron dose) implementing multi-nutrient supplementation or home fortification as a public health measure should be carefully monitored until this has been demonstrated. The World Health Organization recommendation that iron should be supplemented to iron-deficient children needs reconsideration, not because we claim that our study provides conclusive evidence that targeting this group is unsafe, but because the current guideline is based on weak evidence from a single study that is contradicted by our findings using highly specific markers of iron deficiency.

## Supporting Information

Figure S1 **Participant flow over time.** The black line indicates the cumulative number of children in the trial. Recruitment started in February 2008 (day 0) and was completed on 1 August 2008 (day 178) upon recruitment of the 612^th^ participant. The dashed, red line indicates the date (12 March 2009; day 401) that the trial was stopped for all participants. The blue line indicates the cumulative children who had been included in the second survey. This survey took place between 9 October 2008 (day 247) and 12 March 2009.
(TIF)

Figure S2 **Effect of multi-nutrient supplementation on malaria rates with various case definitions, by initial iron status.** For explanation, see figure 3. When all children were included in the analysis, p-values for differences in effect between iron-deficient and iron-replete children were 0.02, 0.01 and 0.12, for episodes as predefined, with density >10,000 and with density >100,000 parasites/µL respectively. Slide results were not available for 32 malaria cases; these were imputed as having densities below 10,000 parasites/µL. Adjustment for distance between homestead and dispensary, height-for-age z-scores, mosquito net use and *Plasmodium* infection at baseline led to similar estimates (not shown).
(TIF)

Figure S3 **Effect of multi-nutrient supplementation on malaria rates with various case definitions, by age class.** For explanation, see Figure 3.
(TIF)

Table S1 **Target dose and form of multi-nutrient supplement (including zinc).**
(DOCX)

Text S1 **Study protocol.**
(DOC)

Text S2 **CONSORT checklist.**
(DOC)
