# Supplementary figures and images for: Effect of Supplementation with Zinc and Other Micronutrients on Malaria in Tanzanian Children: A Randomised Trial
Source: PLoS Med. 2011 Nov 22;8(11):e1001125. doi: 10.1371/journal.pmed.1001125 (PMC3222646; doi:10.1371/journal.pmed.1001125)

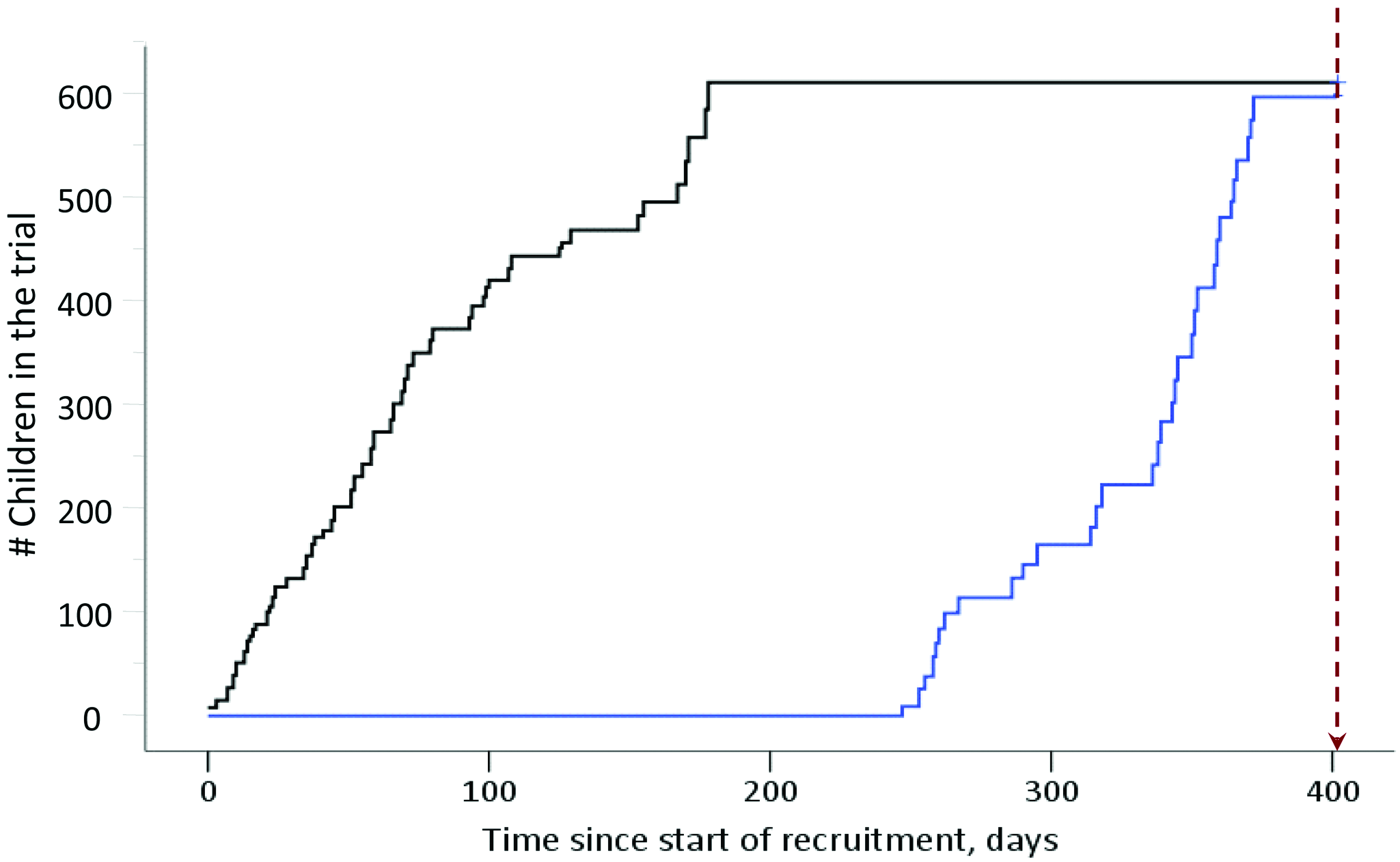

Supplement: Figure S1 — Participant flow over time. The black line indicates the cumulative number of children in the trial. Recruitment started in February 2008 (day 0) and was completed on 1 August 2008 (day 178) upon recruitment of the 612th participant. The dashed, red line indicates the date (12 March 2009; day 401) that the trial was stopped for all participants. The blue line indicates the cumulative children who had been included in the second survey. This survey took place between 9 October 2008 (day 247) and 12 March 2009. (TIF) [file pmed.1001125.s001.tif]

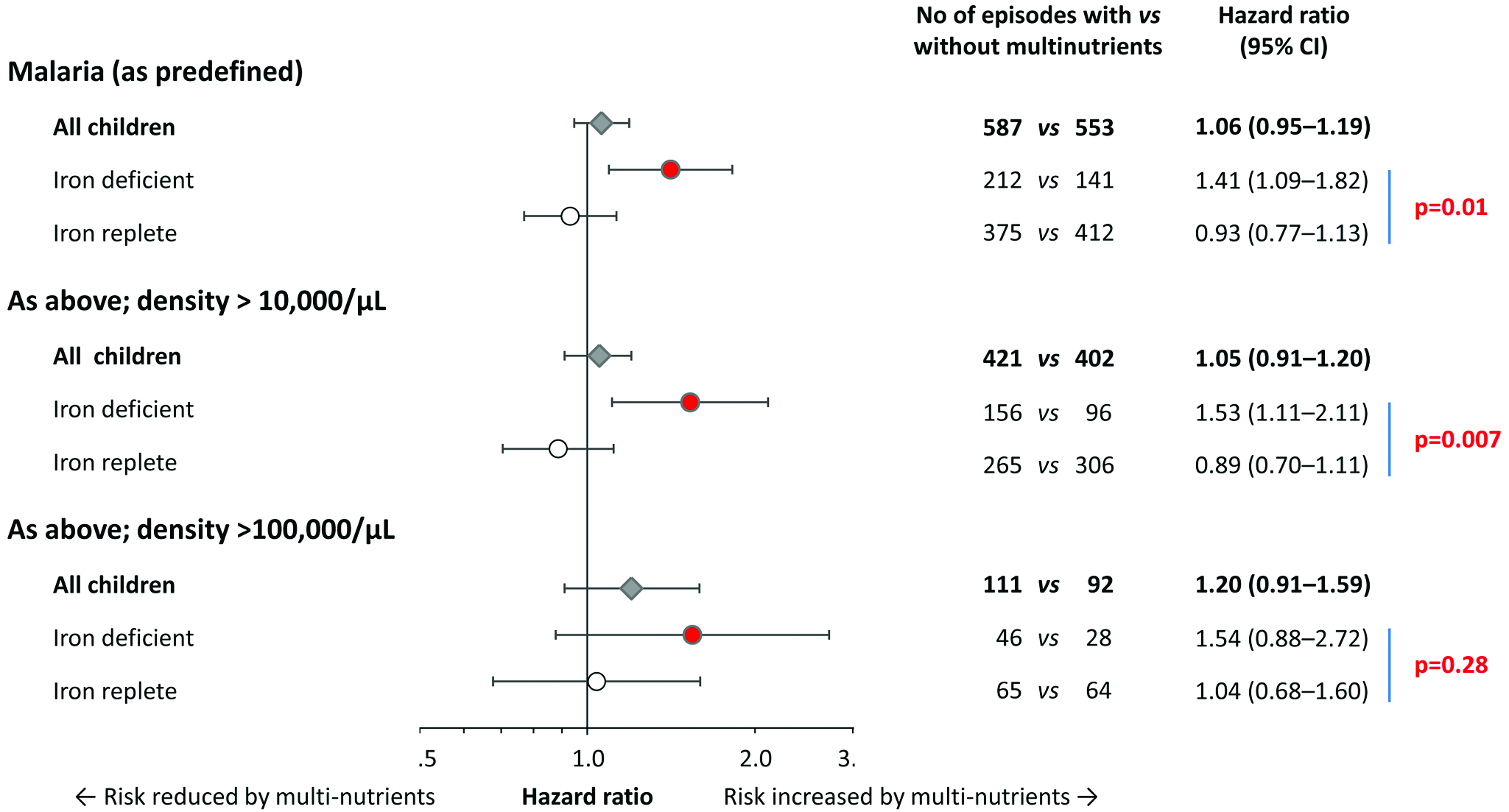

Supplement: Figure S2 — Effect of multi-nutrient supplementation on malaria rates with various case definitions, by initial iron status. For explanation, see figure 3. When all children were included in the analysis, p-values for differences in effect between iron-deficient and iron-replete children were 0.02, 0.01 and 0.12, for episodes as predefined, with density >10,000 and with density >100,000 parasites/µL respectively. Slide results were not available for 32 malaria cases; these were imputed as having densities below 10,000 parasites/µL. Adjustment for distance between homestead and dispensary, height-for-age z-scores, mosquito net use and Plasmodium infection at baseline led to similar estimates (not shown). (TIF) [file pmed.1001125.s002.tif]

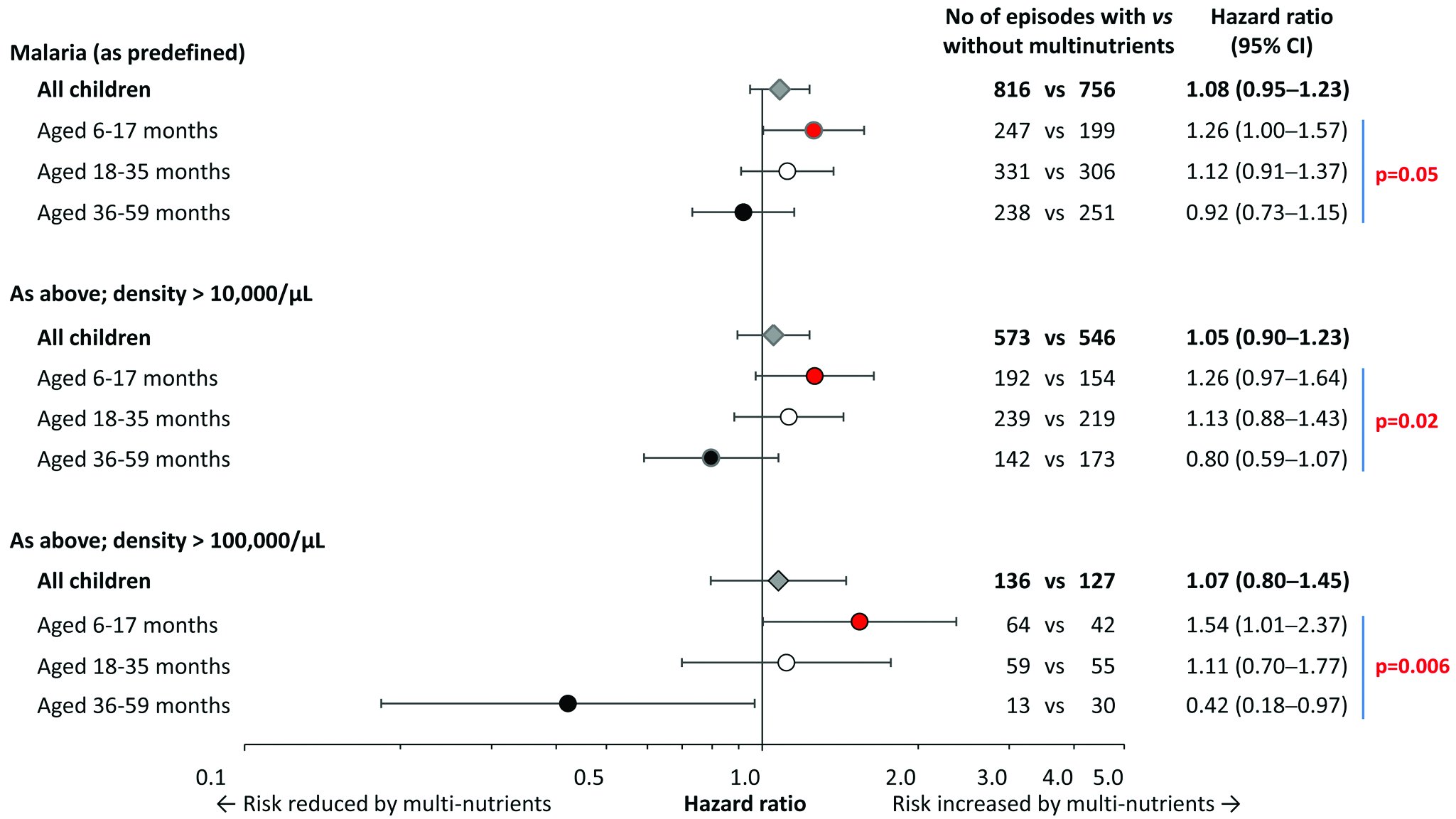

Supplement: Figure S3 — Effect of multi-nutrient supplementation on malaria rates with various case definitions, by age class. For explanation, see Figure 3. (TIF) [file pmed.1001125.s003.tif]
