# Supplementary material for: Effect of Supplementation with Zinc and Other Micronutrients on Malaria in Tanzanian Children: A Randomised Trial
Source: PLoS Med. 2011 Nov 22;8(11):e1001125. doi: 10.1371/journal.pmed.1001125 (PMC3222646; doi:10.1371/journal.pmed.1001125)
Supplement: Table S1 — Target dose and form of multi-nutrient supplement (including zinc). (DOCX) [file pmed.1001125.s004.docx]

**table S1. Target dose and form of multi-nutrient supplement (including zinc) ***

| Active substance | Target level | | | Form | Infants 6‒12 months | | Children 1‒3 years | | | Children 4‒5 years | |
| --- | --- | --- | --- | --- | --- | --- | --- | --- | --- | --- | --- |
|  | Declared | Overage † | Formulated |  | RNI | UL | RNI | UL | RNI | | UL |
| Vitamin A | 300 µg RAE | 50% | 450 µg RAE ‡ | All-*trans* retinyl acetate (powder) | 400 µg | 600 µg | 400 µg | 600 µg | 450 µg | | 900 µg |
| Vitamin B_1_ | 0.5 mg | 25% | 0.625 mg | Thiamin mononitrate | 0.3 mg | ND | 0.5 mg | ND | 0.6 mg | | ND |
| Vitamin B_2_ | 0.5 mg | 10% | 0.55 mg | Riboflavin | 0.4 mg | ND | 0.5 mg | ND | 0.6 mg | | ND |
| Niacin | 6 mg NE | 10% | 6.6 mg | Niacine | 4 NE | ND | 6 NE | 10 mg | 8 NE | | 15 mg |
| Vitamin B_6_ | 0.5 mg | 15% | 0.575 mg | Pyridoxine | 0.3 mg | ND | 0.5 mg | 30 mg | 0.6 mg | | 40 mg |
| Folate | 150 µg DFE | 25% | 93.75 µg § | Folic acid | 80 µg | ND | 160 µg | 300 µg DFE | 200 µg | | 400 µg DFE |
| Vitamin B_12_ | 0.9 µg | 30% | 1.17 µg | Cyanocobalamid in mannitol | 0.5 µg | ND | 0.9 µg | ND | 1.2 µg | | ND |
| Vitamin C | 50 mg | 50% | 75 mg | Purified L-ascorbic acid | 30 mg | ND | 30 mg | 400 mg | 30 mg | | 650 mg |
| Vitamin D | 5 µg | 35% | 6.75 µg ¶ | Vitamin D_3_ (cholecalciferol) | 5 µg | 25 µg | 5 µg | 50 µg | 5 µg | | 50 µg |
| Vitamin E | 6 mg TE | 10% | 6.6 mg | RRR-α-tocopherol acetate | 0.6 mg/kg bw | ND | 6 mg ¶ | 200 mg | 7 mg ¶ | | 300 mg |
| Vitamin K | 30 µg | 50% | 45 µg | Phylloquinone (vitamin K_1_) 5% | 10 µg | ND | 15 µg | ND | 20 µg | | ND |
| Zinc | 10 mg | 0% | 10 mg | Zinc as gluconate | 8.4 mg | 5 mg | 8.3 mg | 7 mg | 9.6 mg | | 12 mg |
| Iron | 18 mg | 0% | 18 mg | Ferrous fumarate | 18.6 mg | 40 mg | 11.6 mg | 40 mg | 12.6 mg | | 40 mg |
| Iodine | 90 µg | 0% | 90 µg | Potassium iodate | 90 µg | ND | 90 µg | 200 µg | 90 µg | | 300 µg |
| Copper | 340 µg | 0% | 340 µg | Cupric gluconate | 220 µg ║ | ND | 340 µg ║ | 1 mg | 440 µg ║ | | 3 mg |
| Selenium | 20 µg | 0% | 20 µg | Sodium selenate | 10 µg | 60 µg | 17 µg | 90 µg | 22 µg | | 150 µg |
| Magnesium | 65 mg | 0% | 65 mg | Trimagnesium dicitrate anhydrous | 54 mg | ND | 60 mg | 65 mg ** | 76 mg | | 110 mg ** |

RNI: Recommended Nutrient Intake as established by WHO/FAO [1];UL: Tolerable Upper Intake Level as established by FNB/IOM [2]; RAE: retinol activity equivalents; NE: niacin equivalents; DFE: dietary folate equivalents; TE: α-tocopherol equivalents; ND: Not derived

* The taste in each type of supplement was concealed by the addition of a strong liquorice favour

^†^ Overage is calculated from declared amount (D) and formulated amount (F) as O=(F‒D)*100/D

‡ Equivalent to 1,500 IU

§ Based on IOM estimates that 0.5 µg folic acid taken on an empty stomach corresponds to 1 µg DFE

¶ Equivalent to 270 IU

║ Values obtained from FNB/IOM [2]

**References**

1. WHO/FAO (2004) Vitamin and mineral requirements in human nutrition, 2^nd^ ed. Geneva, Switzerland: World Health Organization.
2. FNB/IOM (2004) Dietary Reference Intakes: UL for vitamins and elements. Food and Nutrition Board, Institute of Medicine, National Academy of Sciences. Available at:

<http://iom.edu/Activities/Nutrition/SummaryDRIs/~/media/Files/Activity%20Files/Nutrition/DRIs/ULs%20for%20Vitamins%20and%20Elements.pdf> (accessed 21 June 2011).
