# Supplementary material for: Effect of Supplementation with Zinc and Other Micronutrients on Malaria in Tanzanian Children: A Randomised Trial
Source: PLoS Med. 2011 Nov 22;8(11):e1001125. doi: 10.1371/journal.pmed.1001125 (PMC3222646; doi:10.1371/journal.pmed.1001125)
Supplement: Text S1 — Study protocol. (DOC) [file pmed.1001125.s005.doc]

Effects of supplementation with zinc and other micronutrients on the health, development and well being of African children

**
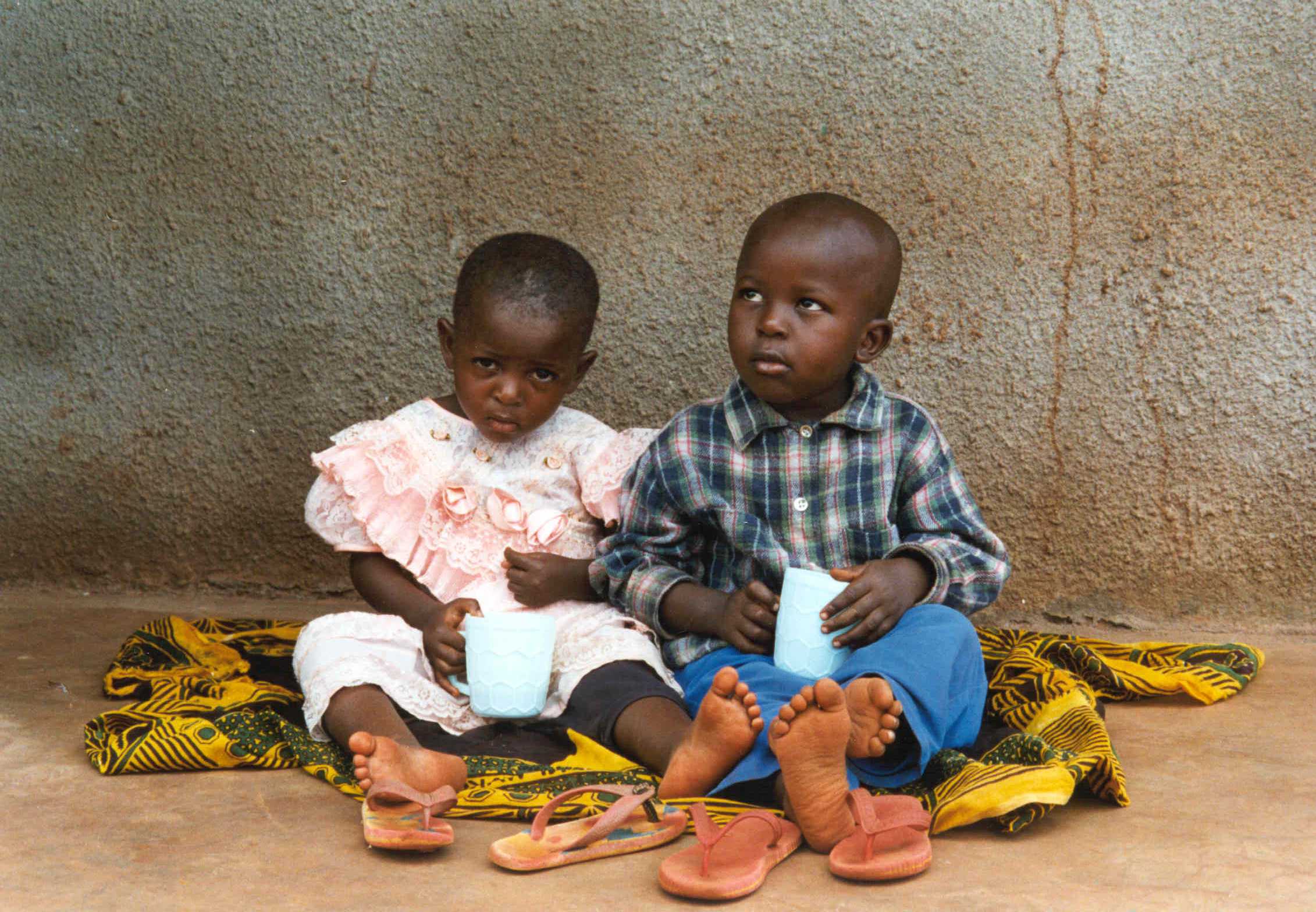
**

proposal submitted for consideration by National Health Research Ethics Review sub-Committee in Tanzania – resubmitted version, February 2007

Applicant:

# Professor Huub Savelkoul

Wageningen University, Cell Biology and Immunology Group

P.O. Box 338, 6700 AH Wageningen, The Netherlands

Tel. 0317 483925/83509; fax 0317 483955; e-mail: huub.savelkoul@wur.nl

**Participating researchers and research groups:**

| Professor Huub FJ Savelkoul (immunologist) Cell Biology and Immunology Group, Wageningen University  P.O. Box 338, 6700 AH Wageningen, The Netherlands  Tel + 31 317 483925; fax + 31 317 483955  E-mail: [huub.savelkoul@wur.nl](mailto:huub.savelkoul@wur.nl) | **Professor John F Shao (medical microbiologist)**  Kilimanjaro Christian Medical Centre  PO Box 3010, Moshi, Tanzania  Tel + 255 27 2754377-2754383 Fax + 255 27 2754381/2752038 E-mail: [jshao@kcmc.ac.tz](mailto:jshao@kcmc.ac.tz) |
| --- | --- |
| Dr Hans Verhoef (epidemiologist/nutritionist) Cell Biology and Immunology Group, Wageningen University  P.O. Box 338, 6700 AH Wageningen, The Netherlands  Tel + 31 317 484421; fax + 31 317 483342  Tel + 255 748 328369 (Tanzania; mobile)  E-mail: [hans.verhoef@wur.nl](mailto:hans.verhoef@wur.nl) | **Dr Raimos Olomi (paediatrician)**  Director, Medical Services  Kilimanjaro Christian Medical Centre  PO Box 3010, Moshi, Tanzania  Tel + 255 27 2754377 Ext 352  Fax + 255 27 2754381  E-mail: [olomirms@kcmc.ac.tz](mailto:olomirms@kcmc.ac.tz) |
| Professor Fons JR van de Vijver (psychologist) Department of Cross-cultural Psychology  Tilburg University  P.O. Box 90135, 5000 LA Tilburg, The Netherlands  Tel + 31 13 4662528/175; fax + 31 13 4662370  E-mail: [fons.vandevijver@kub.nl](mailto:fons.vandevijver@kub.nl) | Dr Chris Drakeley (immunologist)Joint Malaria Programme, Box 2228,Moshi, TanzaniaLecturer, London School of Hygiene and Tropical Medicine, United KingdomTel + 255 27 2753714; Mobile + 254 744 316896E-mail: chris.drakeley@lshtm.ac.uk |
| Dr Dirk J Heslenfeld (psychometrist) Department of Work and Organisation Psychology, Free University, De Boelenlaan 1105, 1081 HV Amsterdam, The Netherlands  Tel + 31 20 6432048/4448700; fax + 31 20 4448702; E-mail: [dirk@psy.vu.nl](mailto:dirk@psy.vu.nl) | **Dr Penny Holding (child development psychologist)**  Wellcome Trust Research Laboratories  PO Box 230, Bofa Road, Kilifi, Kenya  Tel + 254 42 22063 Fax + 254 42 22390  E-mail: [pholding@kilifi.mimcom.net](mailto:pholding@kilifi.mimcom.net) |
| Professor Bernard J Brabin (paediatrician)  Liverpool School of Tropical Medicine  Tropical Child Health Group  Pembroke Place, Liverpool, L3 5QA  United Kingdom  Tel + 44 151 7089393 or 7053207  Fax + 44 151 7088733 E-mail: [**l.j.taylor@liverpool.ac.uk**](mailto:l.j.taylor@liverpool.ac.uk) | **Erasto Vitus Mbugi (molecular biologist)**  Muhimbili University College of Health Sciences (MUCHS), Faculty of Medicine, Dept of Biochemistry, Dar es Salaam, Tanzania/Cell Biology and Immunology Group, Wageningen University  P.O. Box 338, 6700 AH Wageningen, The Netherlands  Tel + 31 317 483925; fax + 31 317 483955 E-mail: [**erasto.mbugi@wur.nl**](mailto:erasto.mbugi@wur.nl) |
| **Professor Jos WM van der Meer (internist)**  Dept General Internal Medicine  University Medical Centre St Radboud  Dept of General Internal Medicine 541  P.O. Box 9101, 6500 HB Nijmegen, The Netherlands  Tel + 31 24 3614763; fax + 31 24 3541734  E-mail: [j.vandermeer@aig.umcn.nl](mailto:j.vandermeer@aig.umcn.nl) | **Jacobien Veenemans (physician)**  Cell Biology and Immunology Group, Wageningen University  P.O. Box 338, 6700 AH Wageningen, The Netherlands  Tel + 31 317 483925; fax + 31 317 483955  E-mail: [jacobien.veenemans@wur.nl](mailto:jacobien.veenemans@wur.nl) |
| **Professor Robert W Sauerwein (internist)**  Section Medical Parasitology, Department of Medical Microbiology (internal postcode 440)  St Radboud University Nijmegen/  University Medical Centre Nijmegen  P.O. Box 9101, 6500 HB Nijmegen, The Netherlands  Tel + 31 24 3614306; Fax + 31 80 540216 E-mail: [**r.sauerwein@mmb.umcn.nl**](mailto:r.sauerwein@mmb.umcn.nl) | **Mary Waka Atieno (educational psychologist)**  Kenya Institute of Education, National Centre for Early Childhood Education, P.O. Box 3703-00200, Nairobi, Kenya/Cell Biology and Immunology Group, Department of Animal Sciences, Wageningen University, P.O. Box 338, 6700 AH Wageningen, The Netherlands. Tel + 254 733 820829  E-mail: [mary.waka@wur.nl](../../../../../Tanzania/Proposals/Ethical%20clearance/Ethical%20clearance%20in%20Tanzania/Submission%20January%202007/mary.waka@wur.nl) |

**Executive summary**

**Background:** Several innovative and complementary approaches are underway to deliver micronutrients to populations in need. First, the International Research on Infant Supplementation (IRIS) initiative – a coalition led by the UN Children’s Fund (Unicef) – has grown in response to expectations that supplementation strategies that simultaneously address multiple micronutrient deficiencies may produce sustainable and effective long-term solutions, as opposed to the use of single-nutrient interventions. Preschool children could benefit most from supplementation, but one of the main operational problems is that infants and toddlers have problems swallowing tablets or capsules that are conventionally used to supplement minerals and vitamins. The IRIS initiative is evaluating newly created supplementation vehicles in the form of chewable tablet-cookies and nutrient-dense spreads suitable for use in tropical environments. The efficacy, safety and acceptability of such new approaches must be evaluated in populations of children with different environmental health challenges.

Second, a global consortium led by some of the world’s leading agricultural research institutes has recently embarked on a programme to breed and disseminate new staple crop varieties with high concentrations of micronutrients. This has the potential to create an entirely new, safe, low-cost and self-sustaining approach to deliver micronutrients to poor farmer families that are difficult to reach through alternative approaches. Because of technical constraints, and contrary to other methods of delivery such as supplementation or fortification, the biofortification approach must focus on single nutrients or at most a few nutrients. This imposes a need to identify micronutrients that are critical to health. Efforts to develop typically ‘African’ crops rich in zinc have so far been limited, and require increased financial and technical support that is probably best obtained when showing compelling evidence of the health benefits of increasing zinc intake.

Zinc is essential for the functioning of the immune and nervous systems. Supplementation trials have shown that increasing zinc intake has great potential to control common infections in children. These exiting findings have been difficult to translate into health policies for African children. First, the evidence has so far been provided by trials that have been conducted almost exclusively in Asia, Latin America, the Pacific, and developed countries. By contrast, the response to supplementation may be different in Africa, where children’s health is challenged by different environmental factors. Malaria is the prime example, with >90% of global deaths occurring in African children, and up to 90% of African toddlers in many areas being infected but symptom-free. Few studies have been conducted to assess the protective effect of zinc against malaria, and their results are contradictory. Simultaneous supplementation with other potentially limiting nutrients may be required to overcome a lack of response when zinc is given alone. Study of the immune responses involved could help clarify this issue, provide clues about mechanisms of acquired immunity against malaria and other common infections, and may ultimately assist in vaccine development. Lastly, there are strong suspicions that zinc deficiency also impairs mental and psychomotor development, so that millions of children never fulfil their human potential.

In 2006, we conducted a pilot survey (n=304) to assess the prevalence of micronutrient deficiencies, and to measure their independent relationships with infection status, indicators of immunity and of mental and psychomotor development. This study showed that our plan to conduct a randomised trial is feasible because both zinc deficiency and malaria were highly prevalent in the study population (63% and 49%, respectively).

**Objectives:** The project proposed aims at measuring effects of supplementation with zinc and other micronutrients, given either alone or in combination, on malaria incidence and nutritional status, on cellular and serological indicators of immunity in Tanzanian children, and on a battery of indicators of mental development, attention, information processing, activity, psychomotor development and neurophysiological performance. Conditional to sufficient additional funds being obtained, we will also assess the effects of supplementation with zinc and other micronutrients on the prevalence of *H. pylori* infection and on small bowel permeability.

**Innovative aspects:** We expect this study to provide new, better and conclusive evidence on the efficacy of zinc supplementation on malaria, cellular or soluble mediators of immunity, and cognitive and psychomotor development. By measuring effects on status for other micronutrients, our study may provide new evidence of deficiencies that have been little studied in children but that are often considered as possible public health problems. This will also contribute to international efforts to develop and evaluate innovative supplementation vehicles suitable for young children in tropical environments. We seek to apply new insights from modern immunology, in combination with novel technical advances and techniques to analyse the immune capacity without the need to wait for actual challenging of individuals with infectious organisms.

The trial proposed provides a unique opportunity to minimise research costs that would have occurred if individual projects were implemented independently. The diverging techniques used by scientists with different fields of expertise in this programme will allow us to integrate work at cell, individual and population level. By linking researchers working in the fields of clinical medicine and other health sciences with those working on the interface with agriculture, the project provides a unique opportunity to seek international solutions for nutritional health problems in agriculture- and food-based approaches, rather than being exclusively restricted to a conventional medicinal approach. The project will provide a platform for international collaboration between African and Dutch scientists, and foresees training of a Tanzanian PhD fellow.

**Methods:** In a randomised controlled trial with a 2×2 factorial design, children aged 6-60 months (n=600) will receive for 52 w daily oral supplements with either zinc or its placebo, and additionally either multiple micronutrients other than zinc or their placebo. Effects will be measured as group differences in the incidence of malaria attacks, biochemical and anthropometric indicators of nutritional status, in cognitive development as assessed at the end of the intervention period by neurophysiological indicators. Serological and cellular mediators of immunity will be measured in blood collected at 6 months, and from children presenting in the course of the trial with symptomatic malaria.

**Organisation:** The studies will be completed in four years. The work will be divided into two PhD projects focused on outcomes in the field of clinical medicine (infectious diseases and nutritional status, neurophysiological indicators) and immunology (cellular and soluble mediators). The PhD projects will use one design as the basis for multidisciplinary collaboration, and for data collection and analysis. Both PhD projects will contribute to the implementation of the field work, with academic support by a broad alliance of researchers from different fields of research (nutrition, epidemiology, clinical medicine, immunology, parasitology, psychology, physiology and biochemistry). The PhD fellowships will be extended to one Tanzanian fellow and one Dutch fellow. Each PhD fellow will receive technical support from a committee of academic advisers. A post-doctoral field coordinator will be the first-line supervisor to the PhD fellows, and will provide theoretical and on-the-job training in design and analysis of health studies. Administrative and financial responsibility for the project will be with the applicant in The Netherlands.

**Introduction**

There is increasing recognition that the health, development and well being of children in developing countries depends to a large degree on a few critical micronutrients. For example, scientific evidence accumulated in the 1980s has shown that vitamin A supplementation can greatly reduce child morbidity and mortality. This has led to the subsequent development and application of interventions that now saves hundreds of thousands of lives annually in developing countries. However, zinc deficiency may be even more widespread in developing countries than vitamin A deficiency. Zinc is essential for the immune and nervous systems; evidence is also mounting that in children, zinc deficiency can dramatically increase the burden of diarrhoea and other deadly infectious diseases. In addition, there are strong suspicions that zinc deficiency irreversibly impairs mental development, so that millions of children never fulfil their human potential.

It has been difficult to translate these exiting findings into health policies for African children. First, the evidence has so far been provided by supplementation trials that have been conducted almost exclusively in Asia, Latin America, the Pacific, and developed countries. By contrast, the response to supplementation may be different in Africa, where children’s health is challenged by different environmental factors. Malaria is the prime example, with >90% of global deaths occurring in African children, and up to 90% of African toddlers in many areas being infected but symptom-free. Few studies have been conducted on the protective effect of zinc against malaria, and their results are contradictory and conflicting.

Second, it has been difficult to deliver zinc to populations in need. Several innovative approaches are now underway that may redress this problem (see also below, under Section ‘Relevance to policies and development’). First, a global consortium led by some of the world’s leading agricultural research institutes has recently embarked on a programme to breed and disseminate new staple crop varieties with high concentrations of micronutrients. This has the potential to create an entirely new, safe, low-cost and self-sustaining approach to deliver zinc to poor farmer families. Development and dissemination of such ‘biofortified’ crops requires large initial research investments and sustained support by national and international policy makers; by contrast to other interventions, however, the long-term recurrent costs are low. Because of technical constraints, and contrary to other methods of delivery such as supplementation or fortification, the biofortification approach must focus on single nutrients or at most a few nutrients. This imposes a need to identify micronutrients that are critical to health. Efforts to develop typically ‘African’ crops rich in zinc have so far been limited, and require increased support that is probably best obtained when showing compelling evidence of the health benefits of increasing zinc intake.

Another but complementary approach is taken by a coalition led by the UN Children’s Fund (UNICEF) (Gross 2001, 2003). This International Research on Infant Supplementation (IRIS) initiative has grown in response to expectations that supplementation strategies that simultaneously address multiple micronutrient deficiencies may produce sustainable and effective long-term solutions, as opposed to the use of single-nutrient interventions. It also considers that issues regarding efficacy, safety and acceptability must be resolved before supplementation of micronutrients at the programmatic level can be justified. Although preschool children could benefit most from supplementation, there are no large-scale programmes to supplement children in this age group. One of the main difficulties is the lack of suitable supplementation vehicles. Infants and toddlers have problems swallowing tablets or capsules that are conventionally used to supplement minerals and vitamins, respectively. The IRIS initiative is evaluating newly created low-cost supplementation vehicles in the form of chewable tablet-cookies and a nutrient-dense spread suitable for use in tropical environments (Briend 2002, Briend and Solomons 2003, Nestel et al. 2003, Lopriore et al. 2004). The risks and benefits of such new approaches must be evaluated in populations of children with different environmental health challenges. For example, routine iron supplementation of children living in communities with a high prevalence of anaemia has been advocated, but under certain, undefined, conditions could lead to increased risk of malaria and other infections (Verhoef et al. 2002b, Oppenheimer 2001). Another concern relates to possible interactions between nutrients. For example, zinc has the potential to competitively interact with the absorption of iron and copper (O’Brien et al. 2000, Lind et al. 2003, Yuzbasiyan-Gurkan 1992), although a recent review concluded that zinc supplementation alone does not appear to have a medically important negative effect on iron status (Fischer Walker et al. 2005). Lastly, the acceptability and storage life of supplements may depend on the cultural backgrounds of their parents and ambient climatic conditions.

# Literature review/Statement of the problem/Rationale

Exclusive breastfeeding during the first 6 months of life is considered sufficient to provide adequate nutrition for infants. The risk of nutrient deficiencies increases dramatically with the introduction of complementary foods in the diets of weaning children, because these foods have lower nutrient densities than breast milk. In addition, these diets are usually based on cereals, which contain high concentrations of antinutrients such as phytates and polyphenols that inhibit the absorption of zinc and iron (Verhoef and West, submitted). Thus, in a recent review, it was concluded that two-thirds of sub-Saharan Africans are at risk of low zinc intake (Brown and Wuehler 2000), whilst in a recent survey in Kenya, it was found that >50% of children living in coastal areas have zinc deficiency as assessed by serum zinc concentration (Mwaniki et al. 2002). Biochemical indicators of zinc status such as serum or hair concentrations of zinc are useful at a population level but poorly predict zinc status or functional impairment in individuals. For this reason, randomised placebo-controlled trials have been used to establish the potential health gains of increasing zinc intake. The vast majority of these trials have been conducted in Asia, Latin America and the Pacific region. They have shown that daily oral supplementation with zinc can dramatically improve growth (Brown et al. 2002), and reduce the morbidity of common infectious diseases (Black 2003b) such as diarrhoea (Roy et al. 1999, Bhutta et al. 1999, 2000), acute respiratory diseases (Rahman et al. 2001) and cutaneous leishmaniasis (Sharquie et al. 2001). There have been no studies to assess the influence of zinc supplementation on the efficacy of routine immunizations, but evidence that it might improve the efficacy of oral cholera vaccine in children (Albert et al. 2003) is consistent with the putative role of zinc in the immune system.

Six community-based randomised trials have been conducted so far to evaluate the effects of zinc supplementation in African children. These were specifically designed to measure effects on *Schistosoma* reinfection (Friis et al. 1997a,b), growth (Bates et al. 1993, Umeta et al. 2000, Kikafunda et al. 1998, Zlotkin et al. 2003) and malaria (Müller et al. 2001); their main results are listed in Table 1. None of these trials were conducted to evaluate possible effects on diarrhoea or acute respiratory infections.

Table 1. **Main results of previous trials that have assessed efficacy of zinc supplementation in Africa**

| **Population** | **Reference** | **Main findings** |
| --- | --- | --- |
|  |  |  |
| Children,  The Gambia | Bates et al. 1993 | No evidence of improved growth |
| Children, Zimbabwe | Friis et al. 1997a,b | Intensity of *S. mansoni* reinfections reduced; lean body mass and weight marginally increased |
| Nursery school children, Uganda | Kikafunda et al. 1998 | Mid-upper arm circumference increased but no or only limited evidence of marginal gains in height or weight |
| Rural children, Ethiopia | Umeta et al. 2000 | Growth rate dramatically increased in stunted children, and less so in non-stunted infants |
| Rural children, Burkina Faso | Müller et al. 2001, 2003 | No evidence of an effect on febrile episodes of malaria (incidence ratio: 0.98, 95% CI: 0.86-1.11); or on anthropometric indices |
| Rural children, Ghana | Zlotkin et al. 2003 | No evidence of improved zinc status or catch-up growth |

###### Zinc in relation to malaria

Only two randomised trials have been conducted to assess the protective efficacy of zinc supplementation on malaria. In Papua New Guinea, zinc supplementation led to a reduction by one-third in malaria-attributable fevers (Shankar et al. 2000). This appears to corroborate findings from The Gambia, where zinc supplementation led to a similarly-sized but statistically non-significant reduction in the mean number of clinic visits for malaria (Bates et al. 1993). However, because the latter trial was not specifically designed for this purpose, ascertainment of malarial episodes was poor, and precision of effect estimates was limited by sample size.

By contrast, in a recent trial in Burkina Faso, zinc provided no or only little protection against malaria (Müller et al. 2001), despite that >50% of children studied had mild to severe zinc deficiency as assessed by serum zinc concentrations, and that they received a millet-based diet providing little protein and absorbable zinc. However, serum zinc concentration is greatly dependent on daily fluctuations, and additionally reacts as a negative acute phase protein (Brown 1998). Thus asymptomatic malaria, which occurred in >70% of children at baseline in the Burkina Faso study, and other infections may give the false impression of zinc deficiency as assessed by serum zinc concentration. The occurrence and degree of zinc deficiency in a population can therefore only be established with certainty by demonstrating health benefits of supplementation in randomised placebo-controlled trials (Gibson and Ferguson 1998, Hotz and Brown 2001).

The findings from these trials may have been discordant because surveillance for the detection of malaria episodes was clinic-based in the studies in Papua New Guinea and The Gambia, whereas it was community-based in the Burkina Faso study. This may indicate that zinc supplementation is more efficacious in preventing severe episodes of malaria than mild episodes. An alternative explanation is that the response to zinc supplementation is marginal in zinc-deficient children who are also deficient in other nutrients known to be essential for immune, or in nutrients that impair zinc absorption or utilisation. This idea seems supported by the results from two trials in Iran. In the first of these trials, zinc supplementation alone failed to improve growth in zinc-deficient children; however, this could be overcome in a second trial in which the same population received supplements with both zinc and other potentially limiting nutrients (Ronaghy et al. 1969, 1974). Children who are deficient in zinc are also often deficient in iron and copper, which are essential for the immune system. In addition to this epidemiological evidence, it is known that retinol may affect zinc absorption by regulating synthesis of zinc-binding protein in the ileum (Christian and West 1998), and that zinc absorption may be counteracted by competitive interactions with other trace elements.

Hence, the critical issue is whether zinc is the first-limiting nutrient in improving health (Sandstead 1999, Solomons et al. 1999, Gibson 2000). If not, then zinc and other micronutrients may act synergistically, and simultaneous supplementation with other nutrients might be required to overcome the lack of effect of zinc supplements when given alone. Although this argument has been used extensively in the literature to explain the lack of response to zinc supplementation, it has never been shown in a trial with appropriate design and analysis. It has also been used to advocate the use of multi-micronutrient supplements in health programmes, as opposed to strategies focusing on single nutrients (Gross 2001).

Lastly, the discrepancies found between the results of the zinc supplementation trials may be because the populations studied may have differed in other factors that determine the absorption and utilisation of zinc. We hypothesize that intestinal infections such as *Giardia intestinalis* and *Helicobacter pylori* may play such roles (see below). *H. pylori* infection appears common in Burkina Faso from an early age onwards (Ilboudo et al. 1998, Cataldo et al. 2004). It seems to occur with a higher prevalence than in Papua New Guinea (Ilboudo et al. 1998, Mitchell et al. 1988), although the interpretation of this difference is difficult because these studies used highly selected patients with different age ranges.

# *Zinc and immunity*

The postulated mechanism for the deleterious effects of zinc deficiency on infections is a reduction in immuno-competence (Fraker and King 2004), particularly in cellular immunity (Fraker et al. 1987), which is reversible by supplementation (Castillo-Duran et al. 1987, Sazawal et al. 1997). Zinc is essential for tissues with rapid cellular differentiation and turnover – such as occur in the immune system and the gastrointestinal tract (Shankar and Prasad 1998) – and for various lymphocyte functions implicated in resistance to malaria (Prasad 2000, Shankar 2000), including production of IgG, interferon-γ and tumour necrosis factor-α, and microbicidal activity of macrophages.

An important drawback in malaria vaccine development is the absence of immune markers that represent clinical immunity. Although individuals in endemic areas show immune responses to a wide variety of *Plasmodium* antigens, none of these predict protective immunity. Further study of specific and non-specific immune responses to zinc supplementation could help clarify the morbidity response to supplementation, provide clues about mechanisms of acquired immunity against malaria and other common infections, and may ultimately assist in vaccine development.

Helper T-lymphocytes (Th cells) play a critical role in the immune response to many human infections, including malaria. Based on new insights on the possible role of cytokines in the immunopathogenesis of malaria, we hypothesise that zinc supplementation increases or activates interleukin (IL)-10-producing regulatory T-cell (Tr) subsets, which in turn would mediate active suppression of various immune responses, and thereby protect against immune-mediated diseases such as malaria. Additionally, we postulate that the chronic cycles of parasite-induced inflammation and the counteracting immune response will educate the immune system over time to develop a strong immunoregulatory network, dependent upon the generation of IL-10 secreting Tr1 cells. The development of these cells will be further boosted by zinc supplementation.

Under the influence of the polarising capacity of several proinflammatory cytokines, increased activity by cytotoxic T-cells might also be expected to result in an antigen-specific Th1 and cytolytic activity (Wellinghausen et al. 1997). These early responses will be subsequently dampened by a later anti-inflammatory response based on the release of IL-4, IL-10, and transforming growth factor (TGF)-ß. This indicates that inhibition of this rapid pro-inflammatory response and Th1 outgrowth, such as may occur in zinc deficiency, can weaken the protective immune response to malaria.

Recent evidence has shown that immunity against falciparum malaria is associated with protective antibodies of different classes and subclasses (Fraker et al. 2000). Depending on the dynamics of disease transmission and on the immune status of the individual, IgG1 and IgG3 antibodies were shown to be protective. IgG3 is the prevailing isotype associated with protection in humans, but has a relatively short half-life in circulation. Thus induction of specific IgG1 antibodies might potentially provide long-term efficient protection against the pathogen. Cytokines such as IL-10, IFN-γ, IL-2 and TGF-ß may be of profound importance, by virtue of their potential to induce isotype switching in B-cells to the induction of antigen-specific IgG1 antibodies. Importantly, the production of precisely these cytokines was shown to be affected by zinc deficiency, leading to decreased levels of innate immunity, but also decreased activity of NK cells and a decreased ability to mount a protective Th1-type immune response. These insights lead us to believe that zinc supplementation will boost the capacity of the immune system to wield a protective response to malaria.

### Zinc and cognitive and psychomotor development

Zinc is essential for brain maturation and functioning, and its deficiency in children may lead to impaired motor development (Bentley et al. 1997, Sazawal et al. 1996, Friel et al. 1993) and perhaps also cognitive development (Kirksey et al. 1994, Sandstead et al. 1998, Hamadani et al. 2001, 2002). The mechanisms involved are poorly understood. In the central nervous system, zinc is present in metalloproteins in neurons and glial cells, and it may play a role in synaptic events of glutaminergic neurons, which are found primarily in the forebrain and connect with other cerebral cortices and limbic structures (Takeda 2001, Black 2003a). Evidence from human and animal studies suggests that these deleterious effects can be reversed or prevented at least in part by supplementation. At least six randomised controlled trials have been examined such effects in preschool children (Black 2003a). Their results are inconsistent and inconclusive, but suggest a model in which neuropsychological functioning (e.g. attention), activity, and motor development mediate the relation between zinc deficiency and cognitive development (Black 1998, 2003a). The instruments used in these trials to assess cognitive functioning have limited capability, which could be overcome in future trials by including neurophysiological tests and a broader range of psychomotor tests. In a recent review (Black 2003a), it was stressed that future trials should address the effects of zinc supplementation in relation to co-existing deficiencies of other nutrients such as iron and vitamin B_12_ (cobalamin) (see also discussion in preceding paragraphs). Mild deficiencies of iodine, vitamin B_1_ (thiamine), B_12_ and perhaps B_3_ (niacin) are widespread in developing countries, and all of these nutrients have been associated with adverse neurological or cognitive outcomes (Grantham-McGregor and Ani 1999).

### Zinc and other micronutrients, Helicobacter infection and intestinal functioning

Morphological studies and findings from a zinc supplementation trial in Bangladeshi children with diarrhoea suggest that zinc deficiency adversely affects intestinal permeability (Moran and Lewis 1985, Roy et al. 1992). This is particularly relevant in view of findings that African children may have a persistent mucosal enteropathy of the small intestine, as shown by sugar permeability tests and confirmed by biopsy studies in rural Gambian infants (Behrens et al. 1987; Lunn et al. 1991a; Sullivan et al. 1991). This enteropathy is strongly associated with stunting and improves with age but persists through adulthood, which raises the possibility that it may continue to limit growth throughout childhood and puberty (Campbell et al. 2002). These findings are consistent with reports from intestinal biopsies collected from symptom-free adult inhabitants of other tropical climates that also show histological evidence of the so-called tropical enteropathy (Lindenbaum et al. 1966, Lindenbaum 1973) that is associated with abnormal gastrointestinal function tests (Baker and Mathan, 1972). Because small intestinal mucosal enteropathy is at its most severe during infancy (Lunn et al. 1991b), it is important to know whether the intestinal changes that occur at this very early stage are persistent in the same individuals and restrain growth throughout the growth period (Campbell et al. 2002). Thus we hypothesize that zinc supplementation results in improved small bowel permeability in symptom-free children.

The etiology of the enteropathy is unclear, but it may be due to infection or inflammation (Campbell et al. 2002), and its causal effects on stunting may be mediated by malabsorption or maldigestion (Lunn 2000). The evidence available suggests that it could be due to *H. pylori* infection (Lunn 2000). *H. pylori* is a Gram-negative bacillus that occurs in the mucus layer of the stomach (Logan and Walker 2001). Humans appear to be the only reservoir, and transmission is probably person-to-person and mostly via the gastro-oral route (Mitchell and Mégraud 2002). Infection is most commonly acquired during childhood, with up to 70% of children in developing countries infected by the age of 15 years (figure 1). The highest seroconversion rates occur in children aged <5 years (Rothenbacher et al. 2000, Malaty et al. 2002). The immunological response to infection is generally not sufficient to clear infection, which often persists for life (Logan and Walker 2001). Findings from a study in Peru, however, suggest that spontaneous elimination of infection may be quite common in preschool children (Klein et al. 1994). Primary acquisition in adults, or reinfection in children or adults after successful eradication, does occur but is less common. Thus it has been recommended that treatment and preventive measures should be aimed at children aged <10 years (Malaty et al. 2002).


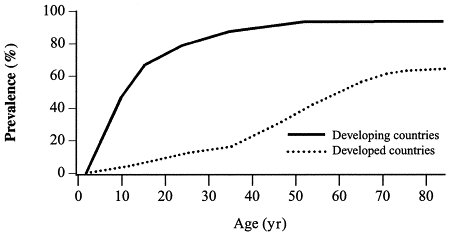
Figure 1. **Prevalence of *H. pylori* infection by age in developing and developed countries** (adapted from Logan and Walker 2001)

*H. pylori* infection invariably leads to chronic gastritis. It is the principal cause of peptic ulcer disease, and the main risk factor for the development of gastric cancer (Logan and Walker 2001). Additionally, it may cause exocrine pancreas cancer (Stolzenberg-Solomon et al. 2001) and perhaps recurrent abdominal pain in children (Roma-Giannikou and Shcherbakov 2002). There is increasing evidence that it may cause iron deficiency anaemia that is refractory to iron supplementation or fortification. For example, in a recent randomised controlled trial in Indian children aged 1-10 years, supplementation with ferrous fumarate resulted in increased haemoglobin concentrations in children without *H. pylori* infection, but produced no or only a marginal haemoglobin response in their peers with *H. pylori* infection (Mahalanabis et al., personal communication, 2004).

Although the mechanisms whereby *H. pylori* infection causes iron deficiency anaemia are not clear, it has been speculated that *H. pylori* can capture transferrin-bound iron, and that this iron becomes lost in stools together with dead bacteria (Barabino 2002). However, it would appear more likely that *H. pylori* infection causes iron deficiency by impairing iron absorption. *H. pylori* is able to survive the acid conditions around the gastric epithelium by generating a neutral microenvironment within and surrounding the bacterium. This is achieved by producing abundant urease, which converts urea to carbon dioxide and pH-neutralising ammonia. Thus iron deficiency anemia associated with *H. pylori* is associated with an increased gastric pH. A low gastric pH favours solubility of non-haem iron and is critical for the subsequent absorption of iron and zinc in the duodenum. Additionally, *H. pylori* infection leads to decreased intragastric concentrations of ascorbic acid. Ascorbic acid is probably the most effective absorption enhancer of non-haem iron when consumed with foods containing phytate, iron-binding phenols or calcium (Hallberg et al. 1986, Lynch and Stoltzfus 2003). Ascorbic acid and other soluble, low-molecular weight organic acids bind zinc and facilitate its absorption (Krebs 2000, Lönnerdal 2000). Thus it would appear possible that *H. pylori* infection may also impair the absorption of zinc.

Diminished acid secretion and decrease in intragastric concentrations of ascorbic acid in *H. pylori*-induced gastritis may also contribute to vitamin B_12_ malabsorption and deficiency (Yacoob et a. 2003). Additionally, it has been speculated that *H. pylori* infection can cause deficiencies of other micronutrients, including vitamin A, copper, vitamin E and vitamin C (Yacoob et a. 2003). Conversely, it is possible that improved nutrition could boost the immune system, which could lead to delayed acquisition and suppression of infection.

**Objectives**

**General objective**

The studies proposed aim to determine the effects of supplementation with zinc and other multi-micronutrients on the health, development and well being of Tanzanian children.

**Specific objectives**

*Primary objectives:*

To measure, in children 6-60 months receiving for a period of 26 w a daily oral supplement with either zinc or its placebo, and additionally a daily oral supplement with either multiple micronutrients other than zinc or their placebo, group differences in:

1. The incidence of malarial episodes as detected by clinic-based surveillance;
2. Mean height-for-age z-score and median plasma zinc concentration as indicators of zinc status;
3. Status for micronutrients other than zinc, including iron status (median plasma concentrations of ferritin and transferrin receptor) and vitamin A status (median plasma retinol concentration);
4. Cellular and soluble mediators of immunity against malaria and other infectious diseases, determined both in children who are asymptomatic and with febrile episodes;
5. Neurophysiological indicators of cognitive development.

Conditional to sufficient funds being obtained, we will also assess:

1. The prevalence of *H. pylori* infection, and its association with micronutrient status and small bowel permeability;
2. The influence of *H. pylori* infection on the effects of supplementation with zinc and other micronutrients on status for these nutrients;
3. The effects of supplementation with zinc and other micronutrients on the prevalence of *H. pylori* infection and on small bowel permeability;
4. The prevalence of *G. intestinalis* and its association with micronutrient status.

**Findings from a pilot study**

In May-July 2006, we conducted the field work for a pilot survey around Segera, in the north-eastern part of Handeni District, Tanzania. This study had the general aim to assist in the planning of the randomised trial, and specifically: a) to identify communities with a high prevalence of malaria infection, using Lot Quality Assurance Sampling procedures (Lanata and Black 1991, Lemeshow and Taber 1991); b) to foster community cooperation; and d) to test field and laboratory procedures. We obtained informed consent from local government officials, health officials and village leaders, and conducted a census among resident children with their assistance. These data were used to define 19 lots. From each lot, we invited mothers from 16 randomly selected children aged 6-60 months to bring their child for examination in a mobile clinic. After explaining the aims and procedures for the study, informed consent was also obtained from parents, and witnessed by other resident villagers. Children participating and their siblings were treated free of charge and as needed in accordance with the guidelines of the Tanzanian government; when needed, advice was sought from the local co-investigator (Professor Raimos Olomi). Children and adults with emergent conditions but who were not participating in the study were examined, treated or referred as needed. Children and adults without emergent conditions were examined and treated insofar as resources were available, and referred if necessary. All these services were provided free of charge.

A questionnaire was administered to the parents; children were medically examined, anthropometric measurements were taken, electroencephalograms were recorded for children aged 2-3 years, and samples were collected of venous blood (maximally 5.9 mL), urine and stool. Aliquots of the blood were processed in the field to produce blood smears, to detect malaria infection using dipstick tests, to measure plasma C-reactive protein, and for haematological analyses; the remainder of the blood samples were carried daily to Moshi for isolation and storage of peripheral blood mononuclear cells. Stool samples were tested for the presence of *H. pylori* antigen, and of intestinal parasites. Blood, plasma and urine samples were transported to The Netherlands. Plasma concentrations of trace elements were measured by inductively coupled plasma atomic emission spectrometry.

Community cooperation was excellent, with very few refusals. The results obtained so far strongly indicate that it is feasible to measure all analytes described for the randomised trial (see below) with one tube of blood collected (maximally 5.9 mL). Preliminary results obtained from 304 children were as follows (prevalence estimates between brackets): zinc deficiency as indicated by plasma zinc concentration <9.9 mmol/L: 63%; current malaria infection by positive test result for dipstick test (46%); being stunted (39%) and wasted (2%) as indicated by z-scores height-for-age and weight-for height <-2 SD, respectively; *H. pylori* infection by stool antigen test (31%), *G. intestinalis* as detected by microscopical examination of stools (30%). We are currently developing and testing protocols to determine the ex vivo leucocyte response to antigenic stimulation with an extract of malaria parasites (in collaboration with the Department of Medical Microbiology, Radboud University Nijmegen), and to measure biochemical indicators of vitamin status and indicators of immunity.

Methodology

*Study area and population:* The research team will be based at Kilimanjaro Christian Medical Centre (KCMC), Moshi town, northern Tanzania. The study will be carried out in a lowland area is highly endemic for falciparum malaria (around Segera, Handeni District). This area has been selected in close consultation with other research groups working in the area, and is the same as where we conducted our pilot survey (see above). Communities will be eligible for the intervention study when ≥8 of 16 children tested in the pilot survey had falciparum malaria infection as assessed by immunochromatic antigen test for pLDH (see below). With communities of 100 children, this strategy would provide a 90% probability of including a community with a prevalence of infection of >67%, and a 90% probability of excluding a community with a prevalence of infection of < 39%. In smaller communities, this strategy would lead to even better performance to distinguish between communities with high and low prevalence, whereas this performance would be only marginally worse in larger communities.

In neighbouring Muheza District, children aged 6-71 mo are bitten by infectious mosquitoes at frequencies ranging between 35-400 per year, with malaria parasitaemia ranging between 33%-84% (Ellmann et al. 1998). Malaria transmission is perennial but presumably peaks during and following the rainy seasons, which occur between March and May and between October and November. *Plasmodium falciparum* accounts for >90% of all malaria infections. Near Muheza, infants experience on average 3-3.5 symptomatic episodes of malaria per year (Mutabingwa et al. 1992), and resistance to both chloroquine and sulfadoxine-pyrimethamine are widespread (Rønn et al. 1996, Mutabingwa et al. 2001).

The dietary intake of absorbable zinc in the target population is probably low because the dietary intake from animal products is low, and local staple crops (predominantly maize and beans) have high concentrations of phytates and polyphenols that impair the absorption of zinc. The prevalence of anaemia at altitudes ≥1200m is high (Table 2), probably due mostly to iron deficiency. Because absorption of iron is impaired by phytates and polyphenols, similarly to zinc, this would suggest that zinc deficiency is indeed widespread.

*Study type:* This study concerns a double-blind randomised placebo-controlled efficacy trial with a parallel 2 × 2 factorial design.

*Study entry, assessment of eligibility:* All children aged 6-60 months will be invited into the study. Children who are not brought will be recorded (if possible with reason), and parents of children who are brought will be informed of the study aims and procedures (see Annex 1). Children will be medically examined and a questionnaire will be administered to their mothers or caregivers. A venous blood sample (one tube, corresponding to maximally 5.9 mL) will be collected from children meeting the following criteria: aged 6-60 months; permanently residing in the study area; without a reported history of current or recent (<14 d) diarrhoea; being stunted as determined by height-for-age z-score <-2 SD; without signs and symptoms at randomisation suggesting malaria, hepatitis, HIV/AIDS or tuberculosis; parents or caretakers will have given informed consent, and haemoglobin concentration is ≥70 g/L at randomisation. By including stunted children, we expect to select a population that is more zinc deficient than the general population, and that may be expected to benefit more from supplementation, whilst this criterion poorly predicts zinc deficiency in individual children (see preceding sections). Non-stunted children will be excluded because they are likely to be not or less responsive to zinc supplementation.

*Randomisation and experimental treatments:* Children will be randomly allocated to four experimental groups to receive daily oral supplements containing: a) zinc; b) multi-micronutrients without zinc; c) both zinc and other multi-micronutrients; or d) placebos for both zinc and other micronutrients. The allocation code will be generated blindly based on a list of random numbers and immediately after eligibility has been fully established. Supplements will be provided 7d/wk for an average of 61 weeks (6d/wk under supervision by community health workers). A system will be developed to monitor compliance to experimental treatments. Parents, community health workers, field staff and laboratory staff will be blinded to group assignment. Supplements provided to the four intervention groups will be similar in appearance and organoleptic characteristics, but they will be packed in colour-coded packs to facilitate the field work. As foreseen, the supplements will be provided in the form of chewable tablets (see Annex 2 for details on composition) with micronutrient levels based on recommended dietary allowances for these micronutrients (FAO/WHO 2001, IOM 1997, 2000a, 2000b, 2002).

*Recruitment schedule:* Assuming a recruitment rate of 67 children per week, and 600 participating children (see section ‘Study size calculation’ below), we expect all study children to be enrolled within 9 weeks after randomisation of the first child. All children remain in the study until a fixed date so that a total of 704 child-years of observation has been accrued. Children with episodes of common illnesses will remain in the study until the scheduled end of the study period. Thus with a recruitment rate of 67 children per week, we expect the intervention period to be 63 weeks and 57 weeks for the first and last recruited child, respectively.

*Follow-up procedures:* Contact will be established by scheduled daily visits to community health workers who will supervise and provide continued counselling about supplementation. Mothers will be asked to bring or refer children for scheduled monthly examinations, or immediately when having fever, diarrhoea, cough with chest indrawing, cough with elevated respiratory rates, or any time they feel that the child needs medical attention. Mothers and community health workers will be instructed to determine respiratory rates by counting twice for one full minute each ≥45/min (age <12 months) and ≥35/min (age ≥12 months). By setting the cut-off values five breaths/min below reference values determined by the World Health Organisation for fast breathing (WHO 1998, 2000a), we expect to increase the sensitivity of detecting acute respiratory infections and pneumonia. Children will be examined in a central research clinic, and those with symptoms will undergo medical and laboratory examinations as needed or scheduled.

*Measurements in scheduled visits:* Overviews of indicators to be measured at scheduled meetings are provided in Figure 2 and Tables 2 and 3. Additional funds have been requested to collect capillary blood samples (<0.5 mL) at intervals of 10 weeks to determine haemoglobin concentration, and the presence and severity of malarial infection (by immunocapture pLDH activity assay and by microscopic examination of blood smears). Venous blood samples (one tube; maximally 5.9 mL) will be collected at randomisation and at 20 w, and will be shielded from day light. Blood will be centrifuged (2000g for 10 min) within 1 h of collection. Whole blood, plasma and erythrocyte samples will be stored frozen (at or below −80 °C) until subsequent laboratory analysis. Recumbent length (<2 y) or standing height (≥2 y) and body weight (Seca scale, Hamburg, Germany) will be recorded in duplicate. Field staff will be trained in anthropometric techniques, and measurements will be standardised before data collection (UN 1986, Lohman et al. 1988). Stool samples will be collected at randomisation and at 20 w, and will be stored frozen or in sodium acetate acetic acid formalin for subsequent determination of the presence and type of *Giardia* parasites, and *H.pylori* infection. Urine samples will be collected at randomisation and at 20 w for subsequent measurement of iodine concentrations.

*Measurements in sick children:* Capillary blood samples will be collected in children with febrile episodes to assess the presence and severity of malarial infection by microscopic examination of blood smears and by qualitative pLDH test. In case of malaria infection, as shown by either microscopy or dipstick test, one tube of venous blood (maximally 5.9 mL) will be collected to measure concentrations of cytokines and to isolate peripheral blood mononuclear cells for subsequent stimulation experiments (see below). Children will be treated and referred as appropriate and according to guidelines from the Tanzanian Ministry of Health. Treatment for common childhood diseases occurring during the intervention period will be provided free of charge. Those with malaria will be treated with antimalarial drugs that provide clearance of blood stages of parasites, but with a relatively short elimination half-life (tentative selection: artesunate and lumefantrine).

Figure 2. **Overview of scheduled measurements**

Gastrointestinal infections: *G. intestinalis* and *H.pylori*

Table 2. **Nutritional outcomes to be measured at baseline and at the end of intervention**

| **Target** | **Indicator** |
| --- | --- |
| - Trace elements | - Plasma concentrations of zinc, magnesium, copper, iron, calcium, manganese, potassium, phosphate |
| - Iodine | - Urinary iodine concentration, plasma concentration of thyroid-stimulating hormone |
| - Iron | - Whole blood haemoglobin concentration; plasma concentrations of ferritin and soluble transferrin receptor |
| - Vitamin A | - Plasma concentrations of retinol and ß-carotene |
| - Vitamin B_11_ | - Erythrocyte folate concentration |
| - Vitamin B_12_ | - Plasma concentrations of cobalamin and holotranscobalamin II |
| - Vitamin E | - Plasma concentrations of α-tocopherol |
| - Vitamin D | - Plasma concentrations of 25-dihydroxyvitamin D and parathyroid hormone |
| - Protein-energy | - Plasma albumin concentration |
| - Stunting, wasting | - Height-for-age and weight-for-height z-scores |

Table 3. **Immunity outcomes to be measured at scheduled meetings**

| **Target** | **Indicator** |
| --- | --- |
| *Cellular* |  |
| - Cell proliferation | - Proportion of cells producing Ki-67 (protein marker of cell activity) |
| - Apoptosis, necrosis | - Proportion of stainable cells with annexin V (protein that binds to the outer surface of dead cells) or proprium iodide (DNA stain) |
| - Cell subsets | - Proportion of Th1 cells (CD4^+^), naïve/memory T cells (CD45RO/CD45RA^+^); CD4^+^ T-cells expressing IL-4/IL-10 and IFN-γ/IL-10 |
| - Cytokines | - Proportion of cell subsets with intracellular IL-4, IL-10 and TNF-α |
|  |  |
| *Soluble* |  |
| - Previous exposure to malaria | - Plasma concentrations of malaria-specific immunoglobulin classes and subclasses |
| - Acute phase proteins | - Plasma C-reactive protein concentration, IL-4 and IFN-γ |
| - Soluble adhesion markers | - Plasma concentrations of sICAM-1, sCD14 and sIL-2 receptor |
| - Cytokines | - Plasma concentrations of IL-1β, IL-4, IL-5, IL-6, IL-8, IL-10, IL-12, TNF-α and IFN-γ in culture supernatants |

*Withdrawals:* Children will be withdrawn from study when having haemoglobin concentrations <60 g/L with signs of respiratory distress; haemoglobin concentrations <50 g/L without signs of respiratory distress; when moving out of the study area; or when hospitalised or severely sick, with the research clinician judging continuation of the intervention undesirable for medical reasons. Children whose parents refuse further supplementation will be asked for their reasons, and whether they want to continue bring their children for examination, so that they can contribute to the intention-to-treat analysis.

*Laboratory analyses:* Blood samples will be collected in containers free of trace elements and with sodium heparine as anticoagulant (Becton-Dickinson, Franklin Lakes, NJ). Blood will be centrifuged and separated within an hour after its collection to prevent leaking of zinc from erythrocytes into plasma (Frank et al. 2001). Malaria infection will be examined by dipstick test, followed by conventional microscopic examination of whole blood (50 µL) when a positive result has been obtained by dipstick test.

Dipstick tests are highly specific for the presence in blood of antigens of both *Plasmodium falciparum* and any of four human *Plasmodium* species (WHO 2000b). Plasma concentrations of pLDH will be measured quantitatively by immunocapture pLDH activity assay, and qualitatively by immuno-chromatographic antigen detection test (Makler et al. 1998, Piper et al. 1999). These tests require 150 µL and 10 µL whole blood, respectively (Piper et al. 1999). For blood samples with >50 *P. falciparum* parasites/mL (0.001% parasitemia), it has been found that the immuno-chromatographic OptiMAL assay has a sensitivity of approximately 96% (Piper et al. 1999). This test also allows the detection of vivax infections of approximately 200 parasites/mL of blood, and to distinguish between falciparum and non-falciparum malarial infections. It has been tested under field conditions in Central America and sub-Saharan Africa and found to perform well in routine diagnosis of malaria (Piper et al. 1999, Moody 2002). In a study in The Gambia, it was found that for initial diagnosis of Plasmodium falciparum, irrespective of stage, this assay had a sensitivity of 91%, and a specificity of 92%, as compared to examination of thick and thin blood smears by highly experienced microscopists (Cooke et al. 1999).

When using the quantitative ICpLDH assay, the levels of pLDH activity closely mirror the levels of parasitemia in both initial diagnosis and while following patient therapy. By using a combination of antibodies, both tests can not only detect but differentiate between falciparum and non-falciparum malaria. Both assays result in very few instances of false-positive samples, especially with samples from patients recently cleared of malaria infection (Piper et al. 1989). Diagnostic tests based on the detection of pLDH are both sensitive and practical for the detection, speciation, and quantitation of all human *Plasmodium* infections.

Severity of malarial infection will be determined by parasite count. Thick and thin blood films will be stained by Giemsa stain and examined by experienced technicians on the day of collection and cross-checked independently later. The number of parasites present per high power microscope field will be determined and the parasite density calculated from this value and the assumed volume of blood present in one high power field (Greenwood and Armstrong 1991). At least 100 high power (× 1000) fields of the thick films will be examined for malaria parasites before a slide is considered negative.

Biochemical indicators of nutritional status will be determined as measures of efficacy of the interventions, compliance with the intervention, and to determine the adequacy of a biochemical response as a prerequisite for expecting a response in functional outcomes (Bates 2003). Concentrations of trace elements will be measured simultaneously by inductively coupled plasma mass spectrometry. This sensitive technique requires very little plasma (<5 µL) and allows the use of an internal standard such as yttrium to monitor background signal, instrument drift, and matrix effects (Ash and Komaromy-Hiller 1997, Frank et al. 2001). Erythrocyte concentrations of folate and cobalamine, and plasma concentrations of holotranscobalamine II, thyroglobulin, 25-hydroxyvitamin D, parathyroid hormone, folate and cobalamine will be determined by electro-chemiluminescence immuno-assay. Plasma concentrations of ferritin, C-reactive protein, soluble transferrin will be measured by nephelometry. Plasma concentrations of retinol, alpha-tocopherol and beta-carotene will be determined by high-pressure liquid chromatography.

Plasma concentrations of sICAM-1, sCD14 and sIL-2 receptor, and *P. falciparum*-specific antibodies will be measured by enzyme-linked immunosorbent assay (ELISA). Plasma concentrations of IL-1, IL-6, IL-8, TNF-α, IL-10, IL-12, and IL-4, IL-5, IL-10, IL-12, IFN-γ, IL-12 will be determined using CBA inflammation and Th1/Th2 kits, respectively (Becton and Dickinson, Franklin Lakes, NJ). Peripheral blood mononuclear cells isolated by standardised Ficol density centrifugation will be washed with medium and either analysed directly or frozen in liquid nitrogen using procedures that ensure maximum responsiveness after thawing. Plasma samples will be stored within 6 h after centrifugation in cryovials at –80ºC until subsequent biochemical analysis. We will measure polymorphisms that may determine the incidence of malaria and that may confound the estimated treatment effects, including α^+^-thalassaemia (Liu et al. (2000), polymorphisms for toll-like receptors (Mockenhaupt et al. 2006) and glutatione-S-transferase (Kavishe et al. 2006). If the blood volume collected allows, we will freeze small aliquots collected at baseline and at 20 w for subsequent determination of metallothionein mRNA (Cao 2000, 2001) to assess their potential for improved indicators of zinc status.

Stool samples will be stored frozen or in sodium acetate acetic acid formalin until subsequent determination of *Giardia* parasites. Haemoglobin concentrations will be measured by photometer (HemoCue, Ängelholm, Sweden), which requires 10 µL whole blood. Faecal antigen tests to detect *H. pylori* infection are non-invasive, and appear particularly suitable for young children. Both the sensitivity and specificity of these tests are 90%-95% (Logan and Walker 2001, Kabir 2003). PCR has the advantage of allowing the detection of virulence factors such as *cag*A, *vac*A and *ice*A genes. It will be used in children found to be positive by faecal antigen test.

*Definition of primary endpoints*

The primary endpoint is a febrile episode of malaria (Table 4). Lists of secondary outcomes and their definitions are provided in Tables 2 and 4. Because the survival analysis from the study in Papua New Guinea (Shankar et al. 2000) suggests that a reduction in malaria incidence may become evident almost instantly but at least within 50 days of supplementation, we will start measurement of possible effects on malaria immediately after commencement of supplementation.

Table 4. **Definition of endpoints**

| **Outcome** | **Definition (tentative) ^1^** |
| --- | --- |
|  |  |
| - Episodes of malaria | - Axillary temperature ≥37.50 °C with positive result for pLDH test; age-specific case definitions based on parasite density or quantitative pLDH test will be used to improve specificity |
| - Episodes of diarrhoea | - ≥3 loose or watery stools during a 24-h period, as reported by mothers or caretakers |
| - Episodes of severe or moderate dehydration | - *Severe:* ≥2 of the following: lethargic or unconscious, sunken eyes, not able to drink or drinking poorly, reduced skin turgor; - *Moderate:* ≥2 of the following: restless/irritable, abnormal thirst, dry oral mucosa, reduced skin turgor, sunken eyes, or decreased urine output |
| - Episodes of acute lower respiratory infections | - Cough plus respiratory rate ≥50/min (infants) or ≥40/min (older children) or indrawing of the ribs |
| - Episodes of pneumonia | - Either a combination of cough with crepitations or bronchial breathing by auscultation, or episodes of acute lower respiratory tract infection associated with ≥1 of lower chest indrawing, convulsions, not able to drink or feed, extreme lethargy, restlessness or irritability, nasal flaring, or abnormal sleepiness ^2^ |
| ^1^ Based on guidelines from the World Health Organization (WHO 1998, 2000a): final definitions will be determined after thorough review and expert consultation; ^2^ Shann et al. 1984, Smyth et al. 1998, Usen et al. 1999). | |

**Statistical considerations**

*Study size calculation:* We expect children in our study population who receive supplements without active substances (double-placebo) to experience malaria with a frequency of 1.50 episodes per year. This estimate is quite arbitrary because it depends on many environmental factors that can fluctuate in time, but seems reasonable considering values reported elsewhere (Lusingu et al. 2004, MacKinnon et al. 2005).

We furthermore expect that single interventions (supplementation with either zinc or other micronutrients) will reduce this incidence by 30% (Shankar et al. 2000) from 1.50 to 1.05 episodes/child-year, and that the double intervention (supplementation with both zinc and multi-micronutrients) will further reduce this incidence by another 30% to 0.74 episodes/child-year. We do not expect antagonistic effects between zinc and other micronutrients that would otherwise reduce the precision of the treatment effects (Brittain and Wittes 1989).

We wish to measure an incidence reduction by 30% unambiguously, if the true effect of the intervention actually has this anticipated value. We therefore decided on a minumum probability of 80% that the 95%CI excludes the null value.

Lastly, we anticipate that the fraction of children who stop taking supplements during the intervention period, *p*, is 0.10, and that these children experience malaria episodes in the same frequency as children not receiving supplements. Taking into account that we will retain these children in the intention-to-treat analysis, we adjusted the sample size for this drop-out by a factor 1/(1-*p*)^2^ (Wittes 2002).

With an effective sample size of 142 child-years per group, and with values for the malaria incidence in the reference group of 1.50 episodes per child-year and 1.05 episodes per child-year, the probability of excluding the null value from the 95% CI would be 92% and 80%, respectively (Figure 1). With these incidence values, the 95% CIs for the incidence ratio of 0.70 would be 0.57 to 0.86, and 0.55 to 0.90, respectively. Adjusted for 10% drop-out, this amounts to a *total sample size requirement of 704 child-years* (176 child-years per group). The precision achieved with this sample size on other indicators is shown in Table 5. Based on previous experience, we estimate that 50 children would yield sufficient precision in estimating production of IFN-γ versus IL-4.

**Figure 1.** Probability of excluding the null value from the 95%CI as a function of the effective sample size, expressed in child-years observation per group. Computations are based on methods by Smith and Morrow (1991), assuming that the incidence in the reference group is 1.50 per child-year (red line), 1.05 per child-year (blue line) and 0.70 per child-year (pink line); interventions result in an incidence ratio of 0.70; and α=0.05. The dotted line indicates the intended sample size of 142 child-years per group (see text).

*Data entry, analysis, interpretation and reporting:* Data will be entered and cleaned using Access software (Microsoft, Seattle, WA); z-scores of anthropometric indices will be computed using EpiInfo software (Centers for Disease Control and Prevention, Atlanta, GA). To define deficiency, we will use recommended cut-off levels for indicators of nutritional status (Bates 2003). The ratio of IFN-γ to IL-4 production will be interpreted as a quantitative indicator of Th1 versus Th2 differentiation, and IL-10-producing regulatory T-cell sets as a possible determinant of the extent and severity of infection. For data analysis, we will use SPSS (SPSS Inc., Chicago, IL) software.

Data will be analysed according to a pre-established plan using an intention-to-treat primary analysis. Multivariate regression will be used to evaluate the extent to which effect on the incidence of malarial episodes in children receiving supplementation with zinc and other micronutrients is greater than the sum of that attained by either zinc alone or micronutrients other than zinc alone. If there is evidence of such interaction, then effect estimates for each intervention will be reported for each of the two strata defined by the other intervention, to indicate the range of effect sizes that may be expected with each intervention alone. If there is no evidence of such interaction, then main effects of the interventions will reported, adjusted for the effect of the other intervention. The effect thus reported for zinc alone would be of particular relevance to efforts to develop biofortified crops rich in zinc, while it would be of biological interest to measure the effect of supplementation of micronutrients other than zinc alone. Lastly, the effect of combined supplementation with zinc and other multimicronutrients will be reported as an indication of what could be achieved in programmes to provide multi-micronutrients. Multivariate techniques will be used to adjust for differences in factors that will be a priori defined on the basis of the findings of the preliminary survey. Effects on incidence rates will be reported as incidence ratios, attributable fractions and child-years of supplementation required per incident case of malaria prevented.

Survival analysis will be used to assess effects on time until first episode of malaria. In exploratory analyses, multivariate techniques will be used to identify subgroups that benefit more than others from the interventions. In particular, we hypothesize that the effects of interventions on the primary outcomes depends on age, height-for-age, weight-for-height, plasma zinc concentration, *H. pylori* infection. Results will be reported according to established guidelines (Moher et al. 2001, Altman et al. 2001). Treatment effects on continuous outcomes will be analysed by comparing group means, and by multivariate and non-parametric methods as appropriate. Treatment effects will also be evaluated on nutritional indicators as determined at the end of the intervention period. Analysis of repeated measures will be used to assess effects on indicators that are measured on a monthly basis (plasma zinc concentration and blood haemoglobin concentration).

Table 5. **Anticipated effects, and precision achieved with a sample size of 176 children per group**

| Outcome | **Placebo group** | **Comparison group** | **Difference expected** | **95% CI** |
| --- | --- | --- | --- | --- |
| Episodes of diarrhoea ^1^ | 1.50 ^3^ | 1.23 ^3^ | 18% | -7% to 43% |
| Episodes of acute lower respiratory infections ^1^ | 1.62 ^4^ | 1.25 | 23% | -1% to 46% |
| Plasma zinc concentration, µmol/L | 9.9 ^5^ | 7.3 | 2.6 ^6^ | 0.1 to 5.1 |
| Height-for-age, z-score | -1.68 ^5^ | -1.18 | 0.50 ^**^ | 0.64 to 1.11 |
| Calculations assume 10% of children being lost to follow-up or withdrawn before the scheduled end of observation. ^1^ Incidence (number of first episodes per child-year observed over a 26-week period); estimates from ^2^ Shankar et al. (2000),^3^ Bhutta et al. (1999), ^4^ Bhandari et al. (2002), ^5^ Mwaniki et al. (2002),^6^ Osendarp et al. (2001), and ^7^ data from Ethiopia by Umeta (personal communication, 2002). | | | | |

*Time schedule/outputs:* Preparations for the project have started in The Netherlands on 1 July 2004. A pilot study was conducted in 2006. We expect to complete the field work for the trial within 63 weeks after randomising the first child, which will start soon after the ethical clearance certificate has been received. We will subsequently need 9 months to prepare manuscripts and PhD theses.

*Management:*

1. All persons will be employed according to KCMC regulations and procedures, and with approval from KCMC.
2. The implementation of the field work will start only after the Principal Investigator has received written approval from the Tanzanian authorities (ethical clearance, research permits);
3. Cooperation with the local counterpart co-investigator:
   1. The Research Coordinator (Dr. Verhoef) and the local co-investigator will meet fortnightly to report on progress made and to plan future activities;
   2. Dr. Verhoef will submit a monthly progress report to KCMC;
   3. The local co-investigator will visit the field at least monthly to supervise the field work;
   4. A budget will be defined for expenditures related to coordination and field visits by the local co-investigator.
4. Once ethical clearance has been provided, we will establish in consultation with senior KCMC staff
   1. a Trial Oversight Committee comprising independent Tanzanians to monitor all activities by the research team;
   2. a Data Monitoring Board to serve and protect trial participants, and to assist and advise the Principal Investigators and the Field Coordinator so as to protect the validity and credibility of the trial.

*Budget/sponsors:* Funds have been secured (see Annex 4) through grants from Netherlands Foundation for the Advancement for Tropical Research (NWO/WOTRO), Wageningen University, the Centre for Poverty-Related Infection-Oriented Research, PRIOR) and the UN Children’s Fund (Unicef). The funding of our research project by the main funding agency (NWO/WOTRO) comprises two main components. First, WOTRO awarded a personal grant to a Dutch clinician (Mrs Veenemans; *curriculum vitae attached*) to assess the effects of micronutrient supplementation on malaria, infectious diseases, growth and biochemical indicators of nutritional status. Following this reward, WOTRO awarded an Integrated Programme, which includes a net grant for a Tanzanian PhD fellow (*curriculum vitae attached*) to assess the effect of the same intervention (micronutrient supplementation) on cellular and soluble mediators of immunity. Under the grant provisions for the WOTRO Integrated Programme, the financial support for the Tanzanian PhD fellows is divided between a living allowance and research budget, whereby the former is expected to cover all personal costs, including housing, medical costs, insurances, travel to/from work. Additionally, the following costs are not covered by these WOTRO grants and the Unicef grant:

- Salary costs of others than the researcher(s), e.g. supervisors who are already employed by a research institution.
- Costs for congress visits, print costs of thesis, and other expenses etc.
- For researchers from developing countries receiving a net grant: all personal expenses which are covered by the living allowance (see above).
- Capacity building other than the training of local PhD fellows (grants specifically intended for capacity building can be obtained on a competitive basis from WOTRO).

The PRIOR grant foresees in approximately 10% of our budget and is intended to support capacity building at KCMC. The disbursement of his grant will be in agreement between KCMC, Wageningen University and the PRIOR Secretariat in The Netherlands (see section ‘Local capacity building’ below).

**Ethical considerations**

The present proposal is in compliance with the ethical guidelines for research (WMA 2000, CIOMS 1992a,b, 1993, 2002). It has received clearance by the ethical review committee for the district Ede-Wageningen in The Netherlands and the Kilimanjaro Christian Medical Centre in Moshi. Several specific issues are discussed below, together with a reference to the corresponding article in the Declaration of Helsinki (WMA 2000).

1. Locally accepted procedures will be used to obtain informed consent from communities and individuals participating in the study. Parents will be thoroughly informed in their local language about the aims and procedures of the study, that participation is voluntary, that they can withdraw consent at any time, and that withdraw of consent will not lead to repercussions. As foreseen, we will collect venous blood up to three times for each child during the intervention period (at the start and the end of the intervention, and when presenting with symptoms of malaria). Parents and an auxiliary worker (community health worker, traditional birth attendant or community elder) will be invited to be present during invasive procedures (venipuncture) so that they can observe and comfort the child. Parents, an auxiliary worker and project staff will judge whether a child must be withdrawn because he/she resists the procedures. An information sheet and a consent form will be developed in close collaboration with health and civil authorities in the area, and with community leaders. (Articles 9, 20-24)
2. Confidentiality of patient information obtained during the study will be maintained. After data entry and cleaning, a code will be used and kept with an independent person so that the anonymity is maintained. (Article 11)
3. Information about funding and sponsors is provided below (see section ‘Budget’). The senior researchers (Professor Shao, Professor Savelkoul, Dr Verhoef) have no potential conflict of interest. (Article 13)
4. Clinical responsibility of children participating in the studies will be with a Tanzanian paediatrician, who will also supervise the PhD fellows in clinical aspects of their work. (Article 15). Children will be assessed and treated by a Tanzanian Clinical Officer, who carries direct responsibilty for patient care.
5. Zinc gluconate is listed as ‘generally regarded as safe’ (GRAS) by the US Food and Drug Administration. The World Health Organization and the Food and Agriculture Organization of the UN recently considered that a daily intake of 23-28 mg zinc is unlikely to pose risk of adverse health effects from excess in apparently healthy children (FAO/WHO 2002). Our supplemental dose of 10 mg/day is based on evidence of the benefit from previous randomised trials that provided zinc at the same dose (median value in 29 trials) to children aged between 7 mo and 8 y (IZiNCG 2004b). In agreement with guidelines for Good Clinical Practice, we will monitor for possible adverse effects of treatment. Because there has been some unproven concern that supplemental zinc may compete for absorption with dietary iron and copper, we will evaluate the effects of the interventions on the status for these nutrients. We will advise parents, however, to provide the supplements apart from meals, so that direct interference between supplemental zinc and dietary iron and copper is avoided. In addition, the International Zinc Consultative Group noted in an authoritative review (IZiNCG 2004b) that no effect on copper status was observed in a study in Indonesian children aged 6 months who daily received 10 mg zinc or placebo for 6 months. IZiNCG concluded that further prospective studies of the possible adverse effects of varying levels of supplemental zinc are needed, including studies with higher doses than used in the Indonesian study. Another review concluded that it is unlikely that zinc (10 mg daily ) will have adverse effects on copper and iron absorption and metabolism (Fischer-Walker et al. 2005). This review concluded that ‘zinc supplementation alone does not appear to have a clinically important negative effect on iron status’, and that ‘joint supplementation generally does not negatively affect the biochemical outcomes expected from individual supplementation.’ No studies have reported a reduced iron status as a result of zinc supplementation. There is no evidence that zinc supplemented at the regimen foreseen in our study may cause adverse effects associated with copper deficiency (e.g. copper deficiency anaemia). A prolonged and very high intake of zinc (>60 mg) has been associated with reduced activity of erythrocyte copper-zinc superoxide dismutase (ESOD, an enzyme in erythrocytes that is indicative of copper status), but the clinical importance of this observation remains unknown (IOM 2002). (Article 16)
6. Regarding the importance of the research, we feel supported by expert consensus that studies are needed about the functional effects of zinc, particularly its effects on malaria and other parasitic diseases, and on neurobehavioural development in different groups (IZiNCG 2004a). (Article 18)
7. Our vision on how the population in which the study is carried out will benefit from the research is outlined below (see section ‘Relevance to policies and development’). (Article 19)
8. Children participating and their siblings will be treated free of charge for common childhood diseases occurring in the course of the intervention period, on the basis of guidelines from the Tanzanian Ministry of Health. As foreseen, children with symptoms of malaria and confirmed parasitaemia will be treated using artesunate/lumefantrin. We intend not to treat children detected in scheduled examinations who are symptom-free but who have a malarial infection. We consider this justified for the following arguments:
9. It is not known whether treatment of asymptomatic carriers is beneficial or not. Several studies of intermittent administration of anti-malarial drugs indicate at least some children may benefit from regular treatment. However, there are several counter-arguments. First, asymptomatic infection is needed to develop immunity that may protect them from subsequent severe disease and death; thus treatment of these infections may even, at least in theory, increase their risk of death. Second, several studies have found that asymptomatic carriers are less likely to develop fever and are at higher risk of anaemia than those without parasitaemia (Färnert et al. 1999, Kun et al. 2002, Lusingu 2004, Ofori et al. 2002). The risks and benefits of treating asymptomatic infections likely depend on age and the level of endemicity. Third, evidence from studies using molecular markers suggests that subsequent febrile malaria attacks in children who are asymptomatically infected are mostly due to newly acquired infections, and not due to recrudescence of old infections (Contamin et al. 1996, Babiker 1998, Roper et al. 1998). This implies that treatment of asymptomatic carriers does not protect against subsequent febrile disease, and that any association between asymptomatic infection and subsequent febrile attacks is because children who are asymptomatically infected are at increased exposure to infectious mosquito bites. Fourth, it has been found that malaria infections in infants tend to be rapidly cleared without causing clinical symptoms (Franks 2001).
10. Zinc may be more efficacious in protecting against febrile malaria episodes in children who are chronically infected than in their peers who are treated regularly for asymptomatic infections. Treatment of children with asymptomatic infections may interfere with the mechanisms of immunity whereby zinc protects against febrile malaria episodes. We hypothesize that chronic parasite-induced inflammation (and the counteracting immune response) is essential to educate children’s immune system to develop a strong immunoregulatory network. Zinc may boost this process by increasing or activating interleukin-10 (IL-10) secreting regulatory T cells (Tr1 cells) (which are needed to suppress ongoing immune responses) and thereby protect against immune-mediated pathology.
11. The surveys are times of intense activity; because of logistical constraints, we may not be able to read all the slides from these surveys immediately (blood slides of children with clinical suspicion of malaria will be read the same day). Hence, it would not make a lot of sense to go back and treat if the child is healthy. We will let the parents know that the slides are not read immediately, and instruct them that they must bring their child immediately to the research clinic when sick.
12. We want to measure malarial infection status in monthly surveys to assess the extent to which zinc and other micronutrients can reduce malaria prevalence (this medically important because asymptomatic infection is associated with anaemia). Treatment of children found to be symptom-free but infected in these surveys would effectively make it impossible for us to evaluate treatment effects on the incidence of febrile malaria episodes (primary outcome).

Based on the above, we conclude that there is no ethical obligation to treat children with symptom-free parasitaemia in our trial. If, however, the Ethical Review Committee disagrees with our views and would insist that asymptomatic children are nevertheless treated, then we propose to avoid this dilemma by not measuring malaria infection status in our monthly surveys. (Article 28, 30)

1. There are at present no national policies for supplementation with zinc in Tanzania, and the use of a placebo for zinc is essential to reach conclusions about efficacy about zinc supplementation. There is no conclusive evidence that zinc is beneficial in Tanzanian children or in African populations in malaria-endemic areas. Thus we feel that the use of zinc placebo is justified. The project staff will in no way discourage compliance with the national guidelines for vitamin A supplementation.

Supplementation with multi-micronutrients is not available as an established effective intervention in Tanzania. Thus in the Tanzanian context, multi-micronutrient supplements are a new intervention for which the efficacy and safety must be evaluated in randomized placebo-controlled trials before being approved for general use. The revised CIOMS guidelines (2002) specifically state that placebo may be used as a comparator in trials when there is no established effective intervention. Efficacy can be determined only in placebo-controlled trials. Thus we consider that the use of placebos for both zinc and other micronutrients as proposed in our trial is justified. (Article 29)

Other issues:

1. *Blood volume to be collected:* We will collect one tube of venous blood (Becton-Dickinson Vacutainer for trace element analysis) per phlebotomy. The blood collection system used allows blood to be drawn into the tube by filling and replacing a vacuum. Thus the flow of blood stops automatically when the vacuum in the tube has dissipated. Although the label on these tubes indicate that 7 mL can be collected, we found in the pilot survey that this maximally yields 5.9 mL; for many of the children studied in the pilot survey, the blood volume collected was even less because the vacuum in the tubes is gone earlier. Thus the volume actually collected will be maximally 5.9 mL, and often less because the vacuum in the tubes will have disappeared earlier. It should be noted that there are no collection tubes commercially available that are both suitable for trace element analysis and yield smaller blood volumes. *Thus it is technically not possible to collect smaller blood volumes for our study (e.g. 5 mL instead of 5.9 mL).*

A blood volume of 5.9 mL is minimally required to conduct the large numbers of tests foreseen, even with the special biochemical tests we will use and that require minimal volumes of whole blood or plasma. In Annex 5, we discuss the ethical issues about the frequency, volume and method of blood collection, the discomfort caused to the child, and the probability and extent that this would cause adverse effects. It is highly unlikely that our procedures for the collection of venous blood would lead or contribute to anaemia in any of the children studied. When collecting capillary blood in sick children, we will use automated lancets that cause minimal discomfort to children.

1. Archived biological specimens will be used strictly for the purposes for which ethical clearance and informed consent have been obtained. When these are required for another experiment, we will submit a new application for ethical clearance.
2. Regarding article 20 of the CIOMS (2002) guidelines, we foresee that this project will have a strong local capacity building component (see separate Section below).
3. Most of the immunological, biochemical and parasitological tests will be conducted in Tanzania, and we will seek opportunities to train local laboratory personnel for this purpose. It should be noted, however, that some of the tests require sophisticated equipment that is not available in Tanzania, or that is prohibitively expensive or requires highly specialised personnel (e.g. ICP-MS equipment). Additionally, few laboratories have the capacity to conduct the variety of biochemical tests that are foreseen in our project. The analyses will be conducted in laboratories with a track record in measuring these indicators, and that have internal and external quality control procedures in place. Thus, we foresee that some of the tests may have to be conducted abroad. In that case, we will seek opportunities for our Tanzanian partners to become involved in the analyses and to be trained in the techniques employed. Export of biological specimens will be in full compliance with the regulations in Tanzania.
4. We do not foresee any patents arising from this work; opportunities will be provided to Tanzanian colleagues to participate in the work, so that joint authorship of scientific papers is justified.
5. Disposal of worn out equipment(s) or all equipments at the end of the collaboration shall be determined jointly by KCMC and Wageningen University but all equipments which have been transferred to Tanzania shall remain in Tanzania.

Innovative aspects

We expect this study to provide new and conclusive evidence on the efficacy of zinc supplementation on malaria, cellular or soluble mediators of immunity, and cognitive and psychomotor development. We expect to have better chances of demonstrating protective effects of zinc than previous trials because, contrary to the study from Burkina Faso, episodes of malaria will be detected using a clinic-based surveillance system, and because we will measuring a broader range of indicators of cognitive and psychomotor development. By measuring effects on status for other micronutrients, our study may provide new evidence of deficiencies that have been little studied in children but often considered as a possible public health problem. This will also contribute to international efforts to develop and evaluate innovative supplementation vehicles suitable for young children in tropical environments.

By using a factorial design, the study will provide novel data on the extent to which underlying nutritional factors determine the response to zinc supplementation and, conversely, the extent to which zinc affects the response to other micronutrients.

Modern immunology has brought conceptual advances such as those concerning Tr1 cells, regulatory IL-10-dependent networks, which have generated new, exciting ways to analyse the immune capacity without the need to wait for actual challenging of individuals with infectious organisms. Our study seeks to apply these insights in combination with novel technical advances and molecular techniques such as Th polarisation assays, intracellular cytokine staining and flowcytometric analysis, cytokine bead array system with flow cytometry.

Randomised controlled trials are unique in being their ability to produce unbiased results, but they are cumbersome, expensive, and logistically complicated in their implementation. The trial proposed provides a unique opportunity to minimise research costs that would have occurred if individual projects were implemented independently. The diverging techniques used by scientists with different fields of expertise in this programme will allow us to integrate work at cell, individual and population level.

Lastly, by linking researchers working in the fields of clinical medicine and other health sciences with those working on the interface with agriculture, the project provides a unique opportunity to seek solutions for nutritional health problems in agriculture- and food-based approaches, rather than being exclusively restricted to a conventional medicinal approach.

Partner institutions and collaboration

The project will foster and expand a partnership between a number of institutes with an ongoing and successful collobaration, in which each partner contributes complementary expertise (Annex 6).

The *Kilimanjaro Christian Medical Centre (KCMC)* of Tumaini University is the tertiary referral centre for approximately 12 million people living in northern Tanzania. It has medical facilities and laboratories that are well-equipped for the studies proposed. The hospital is a huge complex with over 450 beds, with hundreds of outpatients and visitors coming to the centre everyday. Over 1000 staff are employed at KCMC, and the centre is headed by Professor John F Shao (microbiologist). One of the original reasons for establishing KCMC was to be a national teaching centre. Since 1971, training has grown in size, and in 1997, Kilimanjaro Christian Medical College was opened. The quality of clinical services profits a great deal from research and teaching, which both play important roles at KCMC. Since its inception in 1971, KCMC staff members have produced well over 500 scientific papers and lectures. These are mainly concerned with common health problems such as malaria, schistosomiasis, amoebiasis, AIDS, malnutrition and others topics. An infectious diseases clinic was recently established at KCMC with help from the Global Fund to Fight AIDS, Tuberculosis and Malaria. Malaria research is currently being undertaken at KCMC by the *Joint Malaria Programme*, which is an activity established by KCMC in partnership with the National Institute of Medical Research (NIMR), London School of Hygiene and Tropical Medicine (LSHTM) and the Centre for Medical Parasitology, University of Copenhagen, Denmark. The current project will allow the Kilimanjaro Christian Medical Centre (KCMC) to fulfil its wish to expand its research agenda and become involved in nutrition research. In a meeting held in March 2004, it was agreed with KCMC staff and with Dr Chris Drakeley, LSHTM, to try and share basic infrastructure and information so that the project currently proposed will complement the work of the Joint Malaria Program.

Contrary to expectations, and despite a protracted recruitment period, it has been impossible to find applicants from KCMC for the local PhD Fellowships. No Tanzanian applied for the PhD Fellowship in Child Development, despite widely announcing the availability of this position within and outside Tanzania (e.g. KCMC, NIMR, MUCHS, universities in Europe and the USA). Mr Erasto Mbugi (Tanzanian) and Mrs Mary Waka (Kenyan) were recruited from outside KCMC, following recruitment procedures and with approval by KCMC. We foresee possibilities for Tanzanian MSc students to become involved in the research.

*Wageningen University (WU)* is an internationally leading institution with a track record in nutritional and epidemiological research, with extensive links and collaborations with biomedical institutions in developing countries. Wageningen University has contributed substantially to the current project by providing staff time to develop of the proposal in collaboration with its partners. The *WU Cell Biology and Immunology Group* (Head: Professor Savelkoul) will provide expertise on the application of immunological techniques. The three PhD fellows will be registered to obtain their degree at Wageningen University, with Professors Savelkoul as promotor and under co-supervision by Dr Verhoef (see Annex 7). Wageningen University has a broad expertise in conducting randomised trials, including recent community-based trial in Kenya to assess the effect of intermittent administration of iron and antimalarial drugs on anaemia and malaria (Verhoef et al. 2002a,b), and to assess the efficacy of maize flour fortification with iron (Andango et al., in press).

The *Department of Biological Psychology at the Free University, Amsterdam* (Dr Dirk Heslenfeld) will share expertise in neurophysiological measurement of brain functioning in humans. The *Department of Cross-cultural Psychology of Tilburg University* (Professor Fons Van de Vijver) will assist in the interpretation of these test results.

**Professor** Bernard Brabin from the **Child and Reproductive Health Group** at the *Liverpool School of Tropical Medicine* is Professor of Tropical Paediatrics and visiting professor of Tropical Child Health at the *Academic Medical Centre/University of Amsterdam*. He has a particular research interest in the health of pregnant women and children in the tropics, malaria in pregnant women and infants, birth weight patterns, respiratory disease, child growth and nutritional assessment, parasitic diseases in pregnancy and their effect on the infant; childhood anaemia. He will advise the PhD fellow in clinical aspects of the study.

The *University Medical Centre Nijmegen (UMCN)* has extensive expertise in research on clinical and pathophysiological aspects of infectious diseases and public health in developing countries, and collaborates on these issues with institutions in various countries, notably Tanzania and Indonesia. UMCN has a history of 37 years of collaboration with the Tanzania Health system and has been closely involved in development of Tumaini University in Moshi, Tanzania. In the present project, Professor Jos van der Meer from the Department of General Internal Medicine will provide expertise in clinical medicine. Additionally Robert Sauerwein from the Department of Medical Microbiology will share his expertise in basic and clinical research on cytokines and host defence, immunology and molecular biology and vaccine studies of malaria. The current study will be conducted under the auspices of a new, virtual centre (PRIOR), which is coordinated by UMCN. PRIOR brings together several Dutch institutions that are committed to support KCMC and several research institutions in Indonesia in becoming centres of excellence on research in malaria, HIV/AIDS and tuberculosis.

Project structure and integration

Figure 3 provides an overview of the project structure. The work will be divided into two PhD projects focused on outcomes in the field of clinical medicine (infectious diseases and nutritional status, neurophysiological indicators of cognitive development) and immunology (cellular and soluble mediators). The central research question linking these components concerns the benefits on the health, development and well-being of African children of increased intake of zinc and/or other multi-miconutrients, and the underlying mechanisms that might explain these benefits. Integration will be fostered because both projects will use one design as the basis for multidisciplinary collaboration, and for data collection and analysis. Both PhD projects will contribute to the implementation of the field work, with academic support by a broad alliance of researchers in from different fields of research (nutrition, epidemiology, tropical medicine, immunology, parasitology, psychology, physiology and biochemistry).

The PhD fellowships will be extended to one Tanzanian and one Dutch candidate. The field work will be coordinated by post-doc epidemiologist/nutritionist (Hans Verhoef). He will be the first-line supervisor to the PhD fellows, and will provide theoretical and on-the-job training in design and analysis of health studies. Administrative and financial responsibility for the project will be with the applicant (Professor Savelkoul). Each PhD fellow will receive technical support from a committee of academic advisers (Figure 3), and will provide opportunities to committee members to make contributions in study design, implementation, analysis and interpretation of data, and preparation of manuscripts. Results of the research will be reported in peer-reviewed international journals according to established guidelines (International Committee Of Medical Journal Editors 1997, Davidoff et al. 2001).

An international workshop is foreseen to be held at KCMC in 2008. The tentative aims of this workshop are to enhance integration between PhD projects by jointly reviewing the proposal and developing study protocols with all partners involved in the project, to establish a data monitoring committee, and to improve collaboration with the Joint Malaria Programme.

**Local capacity building**

As foreseen, local capacity building will comprise the following components:

1. **Co-supervision of the PhD fellows and the field work by KCMC senior staff**

Senior staff from KCMC will be co-supervising all three PhD fellows (see organizational chart in Section ‘Programme structure and integration’). Professor Olomi, paediatrician, will supervise the implementation of the field work. For this purpose, he will pay regular field visits and meet on average fortnightly with his counterpart (Dr Verhoef). Dr Verhoef will also send monthly progress reports to Professor Olomi and the Chairman of KCMC’s Ethics Review Committee. The costs related to the supervisory role of Professor Olomi will be covered by the project.

1. **Training of local PhD fellows**

One Tanzanian fellow will be trained and is registered for his PhD fellowship at Wageningen University, The Netherlands. The PhD fellowship in clinical medicine is based on a WOTRO personal grant for a Dutch fellow and could not be given to a local candidate.

1. **Technical and other training support**

As foreseen, this would entail several components:

- 1. Sharing of laboratory protocols and, where appropriate, in setting up new assays and establishing new techniques at KCMC.
  2. Professor Savelkoul has offered to teach modules or master classes in immunology at KCMC.
  3. Collection of epidemiological baseline data as a basis for future studies.
  4. Dr Verhoef has offered to contribute to the training of MMed students by assisting in curriculum development and lecturing in formal training courses in the field of epidemiology, biostatistics and micronutrient malnutrition.
  5. MMed students could conduct their research projects in the context of the project, with field supervision by the PhD students and the field coordinator (Dr Verhoef).

1. **Sharing of resources within PRIOR and with the Joint Malaria Programme**
   1. Sharing the costs of the laboratory and some of the administrative facilities.
   2. Possibilities will be explored to submit joint proposals for research projects and capacity building grants to various funding agencies.

#### Dissemination of results

Results will be reported in peer-reviewed international journals according to established guidelines (International Committee of Medical Journal Editors 1997, Moher et al. 2001, Davidoff et al. 2001) and in 3 PhD theses. Additionally, they will be disseminated through scientific conferences, through international alliances established for advocacy and technical support, and through technical agencies of the United Nations. An international workshop will be held at the end of the programme to synthesize and share the results with researchers and/or policy makers. Because of the different professional backgrounds involved, the research team is uniquely positioned to ensure that results are disseminated to key policy makers. The team members are well connected with influential technical agencies, advocacy groups and industry. At the global and regional levels, these include the HarvestPlus Program of the Consultative Group on International Agricultural Research, the International Zinc Nutrition Consultative Group (see below), and key technical agencies of the United Nations such as UNICEF, the World Health Organization, the Food and Agriculture Organization of the UN, and the World Food Program. At the national level, KCMC senior staff is actively engaged in policy forums, working closely with the national Ministry of Health and advising government on the outcomes and implications of research findings.

Dr Hans Verhoef, WU

**(Field coordinator)**

*Funding (partly):*

WOTRO Integrated Programme

Mrs J Veenemans, MD

**(PhD Clinical sciences)**

*Nationality:* Dutch

*Funding:*

WOTRO Personal Grant

*Supervision:*

Prof R Olomi, KCMC

Prof B Brabin, UvA/LSTM

Prof JWM van der Meer, UMCN

Dr H Verhoef, WU

Mr E Mbugi, MVM

**(PhD Immunology)**

*Nationality:* Tanzanian

*Funding:*

WOTRO Integrated Programme

*Supervision:*

Prof R Olomi, KCMC

Prof J Shao, KCMC

Prof H Savelkoul, WU

Dr H Verhoef, WU

Dr C Drakeley, JMP/LSHTM

Prof R Sauerwein, UMCN

Mrs Mary Waka, MEduc

**(PhD Child development psychology)**

*Nationality:* Kenyan

*Funding:*

WOTRO Integrated Programme

*Supervision:*

Prof R Olomi, KCMC

Prof F van de Vijver, TU

Dr D Heslenfeld, VU

Dr P Holding, KEMRI/WT

Dr Verhoef, WU

Prof Huub Savelkoul, WU *

**(Applicant)**

Figure 3. **Framework for collaboration**

* : Promotor for all three candidates; candidates will be registered for their PhD degree at WU

Positions funded by the WOTRO Integrated Programme are indicated in **thick lines**; the PhD project of Mrs Veenemans (**dashed line**) is a WOTRO personal grant.

Abbreviations: JMP: Joint Malaria Programme; KCMC: Kilimanjaro Christian Medical Centre; KEMRI: Kenya Medical Research Institute; LSHTM: London School of Hygiene and Tropical Medicine; LSTM: Liverpool School of Tropical Medicine; TU: Tilburg University; UMCN: University Medical Centre, Nijmegen; UvA: University of Amsterdam; VU: Free University, Amsterdam; WT: Wellcome Trust; WU: Wageningen University

Relevance to policies and development/Limitations of the study

The spectacular gains made in the last decades in the global fight against iodine and vitamin A deficiencies (Mason et al. 2001) have provided useful lessons on how to move from research to public health programmes (Sommer 1998, Underwood 1998). To achieve the goal of a more nutritious diet and better health, sound scientific research should be the foundation for all subsequent actions. Demonstration of efficacy of health interventions – particularly when published in leading international journals – is one of the best methods available for advocacy within the medical establishment, politicians and the public at large (Sommer 1998). Such studies also constitute the first of several consecutive and overlapping phases leading to action (Underwood 1998):

- Description of the magnitude of the problem, identification of risk groups;
- Development of interventions and their integration into public health strategies;
- Development of partnerships for advocacy, technical and financial support;
- Implementation of policies with corresponding political and financial commitment.

Regarding zinc, we are still largely dealing with the first phase. In the process of developing and evaluating public health interventions (De Zoysa et al. 1998), intervention studies should be undertaken to measure efficacy (i.e. maximum effect achieved under highly controlled conditions) before assessing the magnitude of effects achieved under real-life conditions. The efficacy trial proposed will show the potential role of zinc and other micronutrients investigated in improving health, provide guidance as to whether programmes should pursue single interventions or use combined interventions, and set goals that can potentially be achieved using more realistic strategies.

Malaria control is increasingly difficult to achieve, particularly now that chloroquine and other antimalarials rapidly become ineffective due to widespread drug resistance. International and national efforts to control this disease have failed largely because of the lack of effective tools. As a result, malaria continues to cause 1-2 million deaths and >500 million attacks per year in African children. Insecticide-impregnated mosquito nets and chemotherapy continue to be the mainstay of control for the coming decades. A recent authoritative review concluded that, in addition to using mosquito nets and chemotherapy, increasing the intake of zinc and vitamin A are the only interventions that may potentially reduce malarial mortality (Jones et al. 2003). It should also be noted that the reduction in malaria-attributable fever due to zinc supplementation as reported by Shankar et al. (2000) is greater than the effect reported for insecticide-impregnated mosquito nets (Lengeler 2000).

Conclusive evidence of effects of zinc supplementation on cognitive development could be crucial for the development of policies and programmes. For example, recent evidence of impaired mental development of iron deficiency in children, coupled with concerns of possible links with impaired school performance and mental functioning in adults, has been instrumental in forging an international consensus on the need for programmes to control iron deficiency in children (Verhoef et al. 2003).

Growing realisation of the health importance of zinc nutrition in the 1990s has led to the establishment in 2000 of the International Zinc Nutrition Consultative Group (IZiNCG). This alliance of professionals will likely become a leading group for advocacy and technical support, similar to comparable bodies established for iron and vitamin A (the International Nutritional Anemia Consultative Group and the International Vitamin A Consultative Group). Perhaps because realistic interventions to control zinc deficiency are missing, there is at present – and contrary to iodine, vitamin A and iron – little political commitment to combat zinc deficiency. Thus the UN General Assembly and UNICEF recently set international goals to control deficiencies of iodine, vitamin A and iron, but not of zinc (UN 2002).

The current proposal should be considered as part of a wider multidisciplinary approach taken by the research partners to contribute to the control of micronutrient deficiency disorders through agriculture- and food-based approaches and supplementation.

1. Wageningen University (Hans Verhoef) is a partner in a global alliance led by the Consultative Group on International Agricultural Research (CGIAR), which includes the globally leading institutions in research on tropical crops. This multi-million dollar, 10-year research programme (‘HarvestPlus Program’) has started in January 2004, and is undertaken to improve nutrition of poor people in developing countries through breeding of staple crops with increased concentrations of micronutrients (biofortification). The programme will initially focus on six major crops (rice, maize, wheat, sweet potato, cassava and common beans) and three micronutrients (iron, provitamin A and zinc). The work on crops commonly consumed in Africa is so far mainly focused on provitamin A (maize, sweet potato, cassava, rice); increasing zinc concentrations is technically much more challenging and will require large financial inputs. The Nutrition Coordinator for the HarvestPlus Program (Dr Penny Nestel) will be based in Wageningen University and start her duties on 1 January 2004.
2. Another strategy that may prove an effective and low-cost method in improving zinc nutriture involves the application of zinc fertiliser to staple crops. This has the advantage that a single farmer could apply fertiliser in a single or several rounds during the crop cycle, as compared to administrating daily oral supplements to all of her children. The Division of Human Nutrition, Wageningen University is currently conducting a study in China (with the Jiangsu Province Centers for Disease Control) to determine the effect of consuming zinc-fertilised rice on zinc status in children. This project is undertaken in the context of a multidisciplinary research programme at Wageningen University to develop agriculture- and food-based approaches to alleviate micronutrient deficiencies. This programme involves soil scientists, plant breeders, plant physiologists, chemists, and food processing specialists. If successful, the efficacy of zinc fertilisation could be further examined with staple crops in Africa.
3. The study proposed is part of an innovative approach by a UNICEF-led coalition to develop and evaluate new supplementation vehicles in the form of chewable tablet-cookies and a nutrient-dense spread suitable for young children in tropical environments (Briend and Solomons 2003, Gross and Solomons 2003). This evaluation also comprises a multi-country study (Peru, Vietnam, Indonesia, South Africa) to evaluate the efficacy, safety and acceptability of these new vehicles under different conditions. The study presently proposed is the only one to be conducted in children in tropical Africa, who account for >90% of global deaths due to malaria, and also the only one designed with sufficient sample size to assess effects on functional health outcomes in addition to nutritional status.

**References**

Albert MJ, Qadri F, Wahed MA, et al. Supplementation with zinc, but not vitamin A, improves seroconversion to vibriocidal antibody in children given an oral cholera vaccine. J Infect Dis 2003;187:909-13.

Altman D. The revised CONSORT statement for reporting randomized trials: explanation and elaboration. Ann Intern Med 2001;134:663-94.

Ash KO, Komaromy-Hiller G. Analysis of clinical specimens using inductively coupled plasma mass spectrometry. In: Handbook of analytical therapeutic drug monitoring and toxicology (Wong SHY, Sunshine I, eds.). Boca Raton, FL: CRC Press, Inc., 1997:107-25.

Babiker HA, Abdel-Mushin AM, Ranford-Cartwright LC, Satti G, Walliker D. Characteristics of *Plasmodium falciparum* parasites that survive the lengthy dry season in eastern Sudan where malaria transmission is markedly seasonal. Am J Trop Med Hyg 1998;59:582-90.

Baker SJ, Mathan VI. Tropical enteropathy and tropical sprue. Am J Clin Nutr 1972;25:1047-55.

Bao Y, Silva TM, Guerrant RL, Lima AM, Fox JW. Direct analysis of mannitol, lactulose and glucose in urine samples by high-performance anion-exchange chromatography with pulse amperometric detection. Clinical evaluation of intestinal permeability in human immunodeficiency virus infection. J Chromatogr B Biomed Appl 1996;685:105-12.

Barabino A. *Helicobacter pylori*-related iron deficiency anemia. Helicobacter 2002;7(Suppl. 1):71-75.

Bates CJ. Assessment of micronutrient status in mothers and young infants. In: Micronutrient deficiencies in the first months of life. Nestlé Nutrition Workshop Series Pediatric Program, Volume 52. Vevey, Switzerland: Nestec Ltd/Basel, Switzerland: S Karger AG, 2003:1-34.

Bates CJ, Evans PH, Dardenne M, et al. A trial of zinc supplementation in young rural Gambian children. Br J Nutr 1993;69:243-55.

Bayley N. Bayley scales of infant development. 3rd edn. San Antonio, TX: The Psychological Corporation, 1993.

Behrens RH, Docherty H, Elia M, et al. A simple enzymatic method for the assay of urinary lactulose. Clin Chim Acta 1984;137:361-67.

Bentley ME, Caulfield LE, Ram M, et al. A trial of zinc supplementation in young rural Gambian children. J Nutr 1997;127:1333-38.

Bhandari N, Bahl R, Taneja S, et al. Effect of routine zinc supplementation on pneumonia in children aged 6 months to 3 years: randomised controlled trial in an urban slum. BMJ 2002;324:1358-62.

Bhutta ZA, Bird SM, Black RE, et al. Therapeutic effects of oral zinc in acute and persistent diarrhea in children in developing countries: pooled analysis of randomized controlled trials. Zinc Investigators' Collaborative Group. Am J Clin Nutr 2000;72:1516-22.

Bhutta ZA, Black RE, Brown KH, et al. Prevention of diarrhea and pneumonia by zinc supplementation in children in developing countries: pooled analysis of randomized controlled trials. Zinc Investigators' Collaborative Group. J Pediatr 1999;135:689-97.

Bjarnason I, MacPherson A, Hollander D. Intestinal permeability: an overview. Gastroenterology 1995;108:1566-81.

Bjarnason I, Maxton D, Reynolds AP, Catt S, Peters TJ, Menzies IS. Scand J Gastroenterol 1994;29:630.

Black MM. Zinc deficiency and child development. Am J Clin Nutr 1998;68(Suppl):464S-9S.

Black MM. The evidence linking zinc deficiency with children’s cognitive and motor functioning. J Nutr 2003a;133:1473S-76S.

Black RE. Zinc deficiency, infectious disease and mortality in the developing world. J Nutr 2003b;133:1485S-89S.

Bleichrodt N, Bonan E. Iodine deficiency and physical and mental development: final report. Jakarta, Indonesia: Universitas Indonesia, 1977.

Boomsma DI, Beem AL, Van den Berg M, et al. Netherlands twin family study of anxious depression (NETSAD). Twin Research 2000;3:323-34.

Bouvier P, Rougemont A, Breslow N, et al. Seasonality and malaria in a west African village: does high parasite density predict fever incidence? Am J Epidemiol 1997;145:850–57.

Briend A. Possible use of spreads as a FOODlet for improving the diets of infants and young infants. Food Nutr Bull 2002;23:239-43.

Briend A, Solomons NW. The evolving applications of spreads as a FOODlet for improving the diets of infants and young children. Food Nutr Bull 2003;24 (Suppl):S34-S38.

Brittain E, Wittes J. Factorial designs in clinical trials: the effects of non-compliance and subadditivity. Stat Med 1989;8:161-71.

Brown KH. Effect of infections on plasma zinc concentration and implications for zinc status assessment in low-income countries. Am J Clin Nutr 1998;68(2 Suppl):425S-29S.

Brown KH, Peerson JM, Rivera J, Allen LH. Effect of supplemental zinc on the growth and serum zinc concentrations of prepubertal children: a meta-analysis of randomized controlled trials. Am J Clin Nutr 2002;75:1062-71.

Brown KH, Wuehler SE. Zinc and human health: results of recent trials and implications for program interventions and research. Ottawa, Canada: The Micronutrient Initiative, 2000.

Campbell DI, Lunn PG, Elia M. Age-related association of small intestinal mucosal enteropathy with nutritional status in rural Gambian children. Br J Nutr 2002;88:499-505.

Cao J, Bobo JA, Liuzzi JP, Cousins RJ. Effects of intracellular zinc depletion on metallothionein and ZIP2 transporter expression and apoptosis. J Leukoc Biol 2001;70:559-66.

Cao J, Cousins RJ. Metallothionein mRNA in monocytes and peripheral blood mononuclear cells and in cells from dried blood spots increases after zinc supplementation of men. J Nutr 2000;130:2180-87.

Castillo-Duran C, Heresi G, Fisberg M, Uauy R. Controlled trial of zinc supplementation during recovery from malnutrition: effects on growth and immune function. Am J Clin Nutr 1987;45:602-08.

Cataldo F, Simpore J, Greco P, Ilboudo D, Musumeci S. *Helicobacter pylori* infection in Burkina Faso: an enigma within an enigma. Dig Liver Dis 2004;36:589-93.

Cheour M, Leppänen P, Kraus N. Mismatch negativity as a tool for investigating auditory discrimination and memory in infants and children. Clin Neurophys 2000;111:4-16.

Christian P, West KP. Interactions between zinc and vitamin A: an update. Am J Clin Nutr 1998;68(2 Suppl):435S-441S.

CIOMS. Ethics and epidemiology: international guidelines (Bankowski Z, Bryant JH, Last JM, eds.). Geneva, Switzerland: Council For International Organizations Of Medical Sciences, 1992a.

CIOMS. Ethics and research on human subjects: international guidelines (Bankowski Z, Levine RJ, eds.). Geneva, Switzerland: Council For International Organizations Of Medical Sciences, 1992b.

CIOMS. International ethical guidelines for biomedical research involving human subjects. (Bankowski Z, ed.) Geneva, Switzerland: Council For International Organizations Of Medical Sciences, 1993.

CIOMS. International ethical guidelines for biomedical research involving human subjects. Geneva, Switzerland: Council For International Organizations Of Medical Sciences, 2002.

Contamin H, Fandeur T, Rogier C, et al. Different genetic characteristics of Plasmodium falciparum isolates collected during successive clinical malaria episodes in Senegalese children. Am J trop Med Hyg 1996;54:632-43.

Cooke AH, Chiodini PL, Doherty T, Moody AH, Ries J, Pinder M. Comparison of a parasite lactate dehydrogenase–based immunochromatographic antigen detection assay (optimalt) with microscopy for the detection of malaria parasites in human blood samples. Am J Trop Med Hyg 1999;60:173-76.

Cousins RJ. Absorption, transport, and hepatic metabolism of copper and zinc: special reference to metallothionein and ceruloplasmin. Physiol Rev 1985;65:238-309.

Davidoff F, DeAngelis CD, Drazen JM, et al. Sponsorship, authorship, and accountability. Lancet 2001;358:854-56.

Daley LE. Confidence intervals and sample sizes. In: Statistics with confidence: confidence intervals and statistical guidelines (Altman DG, Machin D, Bryant TN, Gardner MJ, eds). London, UK: BMJ Books, 2000:139-52.

De Zoysa I, Habicht JP, Pelto G, Martines J. Research steps in the development and evaluation of public health interventions. Bull World Health Organ 1998;76:127-33.

Elburg L, Hulshof PJM, West CE. Manual for the determination of retinol and carotenoids in blood and human milk. Wageningen, The Netherlands: Wageningen University, Division of Human Nutrition, 1992.

Ellmann R, Maxwell C, Finch R, Shayo D. Malaria and anaemia at different altitudes in the Muheza district of Tanzania: childhood morbidity in relation to level of exposure to infection. Ann Trop Med Parasitol 1998;92:741-53.

Elton M, Winter O, Heslenfeld DJ, et al. Event-related potentials to tones in the presence and absence of sleep spindles. J Sleep Res 1997;6:78-83.

Erhardt JG, Estes JE, Pfeiffer CM, Biesalski HK, Craft NE. Combined measurement of ferritin, soluble transferrin receptor, retinol binding protein, and C-reactive protein by an inexpensive, sensitive, and simple sandwich enzyme-linked immunosorbent assay technique. J Nutr 2004;134:3127-32.

FAO/WHO. Human vitamin and mineral requirements. Report of a joint FAO/WHO expert consultation Bangkok, Thailand. UN Food and Agriculture Organization/World Health Organization. Rome, Italy: Food and Nutrition Division, UN Food and Agriculture Organization, 2001.

Färnert A, Rooth I, Svensson, Snounou G, Bjorkmann A. Complexity of Plasmodium falciparum infections is consistent over time and protects against clinical disease in Tanzanian children. J Inf Dis 1999;179:989-95.

Fischer Walker C, Kordas K, Stoltzfus RJ, Black RE. Interactive effects of iron and zinc on biochemical and functional outcomes in supplementation trials. Am J Clin Nutr 2005;82:5-12.

Fraker PJ, Jardieu P, Cook J. Zinc deficiency and immune function. Arch Dermatol 1987;123:1699-701.

Fraker PJ, King LE. Reprogramming of the immune system during zinc deficiency. Annu Rev Nutr 2004;24:277-98.

Fraker PJ, King LE, Laakko T, Vollmer TL. The dynamic link between the integrity of the immune system and zinc status. J Nutr 2000;130:1399S-1406S.

Frank EL, Hughes MP, Bankson DD, Roberts WL. Effects of anticoagulants and contemporary blood collection containers on aluminium, copper and zinc results. Clin Chem 2001;47:1109-12.

Franks S, Koram KA, Wagner GE, et al. Frequent and persistent, asymptomatic Plasmodium falciparum infections in African infants, characterized by multilocus genotyping. J Inf Dis 2001;183:796-804.

Friel JK, Andrews WL, Matthew JD, et al. Zinc supplementation in very-low-birth-weight infants. J Pediatr Gastroenterol Nutr 1993;17:97-104.

Friis H, Ndhlovu P, Mduluza T, et al. The impact of zinc supplementation on *Schistosoma mansoni* reinfection rate and intensities: a randomized, controlled trial among rural Zimbabwean schoolchildren. Eur J Clin Nutr 1997a;51:33-37.

Friis H, Ndhlovu P, Mduluza T, et al. The impact of zinc supplementation on growth and body composition: a randomized, controlled trial among rural Zimbabwean schoolchildren. Eur J Clin Nutr 1997b;51:38-45.

Gibson RS. Zinc supplementation for infants. Lancet 2000;355:2008-09.

Gibson RS, Ferguson EL. Assessment of dietary zinc in a population. Am J Clin Nutr 1998;68(Suppl):430S-34S.

Grantham-McGregor SM, Ani CC. The role of micronutrients in psychomotor and cognitive development. Br Med Bull 1999;55:511-27.

Greenwood BM, Armstrong JR. Comparison of two simple methods for determining malaria parasite density. Trans R Soc Trop Med Hyg 1991;85:186-88.

Gross R. Micronutrient supplementation throughout the life cycle. New York: UN Children’s Fund, 2001.

Gross R., Dwivedi A, Solomons NW. Introduction to the proceedings of the International Research on Infant Supplementation (IRIS) Initiative. Food Nutr Bull 2003;24(Suppl 3):S3-S6.

Gross R, Solomons NW. Multiple micronutrient deficiencies: future research needs. Food Nutr Bull 2003;24(3 Suppl):S42-53.

Hallberg L, Brune M, Rossander L. Effect of ascorbic acid on iron absorption from different types of meals. Studies with ascorbic-acid rich foods and synthetic ascorbic acid given in different amounts with different meals. Hum Nutr Appl Nutr 1986;40A:97-113.

Hamadani JD, Fuchs GJ, Osendarp SJ, et al. Randomized controlled trial of the effect of zinc supplementation on the mental development of Bangladeshi infants. Am J Clin Nutr 2001;74:381-86.

Hamadani JD, Fuchs GJ, Osendarp SJ, et al. Zinc supplementation during pregnancy and effects on mental development and behaviour of infants: a follow-up study. Lancet 2002;360:290-94.

Hefler L, Grimm C, Leodolter S, Tempfer C. To butterfly or to needle: the pilot phase. Ann Intern Med 2004;140:935-36.

Heslenfeld DJ. Visual mismatch negativity. In: Detection of change: event-related potential and fMRI findings. (J Polich, ed.). Dordrecht, The Netherlands: Kluwer, 2003:44-63.

Heslenfeld DJ, Kenemans JL, Kok A, Molenaar PCM. Brain mechanisms of feature processing. In: Recent advances in event-related potential research. (Ogura C, Koga Y, Shimokochi M, eds.). Amsterdam, The Netherlands: Elsevier, 1996:308-13.

Hotz C, Brown KH. Identifying populations at risk of zinc deficiency: the use of supplementation trials. Nutr Rev 2001;59:80-84.

Huizenga HM, Heslenfeld DJ, Molenaar PCM. Optimal measurement conditions for EEG/MEG source analysis. Psychometrika 2002;67:299-313.

Hurrell RF. Fortification: overcoming technical and practical barriers. J Nutr 2002a;132:806S-12S.

Hurrell RF. How to ensure adequate iron absorption from iron-fortified food. Nutr Rev 2002b:60:S7-S15.

Ilboudo D, Bougouma A, Sombie R, Sawadogo A; Sanou I, Diomande I. Infection a *Helicobacter pylori* chez l'enfant en zone tropicale. Aspects endoscopiques et histologiques. [*Helicobacter pylori* infections in children in the tropical zone. Endoscopic and histological aspects]. Gastroenterol Clin Biol 1998;22:855-57.

International Committee Of Medical Journal Editors. Uniform requirements for manuscripts submitted to biomedical journals. New Engl J Med 1997;336:309-315.

IOM. Dietary reference intakes for calcium, phosphorous, magnesium, vitamin D and fluoride. Washington DC: Institute of Medicine, 1997.

IOM. Dietary reference intakes for vitamin C, vitamin E, selenium, and carotenoids. Washington DC: Institute of Medicine, 2000a.

IOM. Dietary reference intakes for thiamin, riboflavin, niacin, vitamin B6, folate, vitamin B12, pantothenic acid, biotin, and choline. Washington DC: Institute of Medicine, 2000b.

IOM. Dietary reference intakes for vitamin A, vitamin K, arsenic, boron, chromium, copper, iodine, iron, manganese, molybdenum, nickel, silicon, vanadium, and zinc. Washington DC: Institute of Medicine, 2002.

IZiNCG. Research needs. Food Nutr Bull 2004a;25(Suppl):S187-S198.

IZiNCG. Developing zinc intervention programs. Food Nutr Bull 2004b;25(Suppl):S163-S186.

Jones G, Steketee R, Black RE, et al. How many child deaths can we prevent this year? Lancet 2003;362:65-71.

Kabir S. Review article: clinic-based testing for *Helicobacter pylori* infection by enzyme immunoassay of faeces, urine and saliva. Aliment Pharmacol Ther 2003;17:1345-54.

Kaufman AS, Kaufman NL. Kaufman Assessment Battery for Children. Circle Pines, MN: American Guidance Service, 1983.

Kavishe RA, Koenderink JB, McCall MB, et al.. Short report: Severe Plasmodium falciparum malaria in Cameroon: associated with the glutathione S-transferase M1 null genotype. Am J Trop Med Hyg 2006;75:827-29.

Kikafunda JK, Walker AF, Allan EF, Tumwine JK. Effect of zinc supplementation on growth and body composition of Ugandan preschool children: a randomized, controlled, intervention trial. Am J Clin Nutr 1998;68:1261-66.

Kirksey A, Wachs TD, Yunis F, et al. Relation of maternal zinc nutriture to pregnancy outcome and infant development in an Egyptian village. Am J Clin Nutr 1994;60:782-92.

Klein PD, Gilman RH, Leon-Baruda R, et al. The epidemiology of *Helicobacter pylori*. Peruvian children between 6 and 30 months of age. Am J Gastroenterol 1994;89:2196-200.

Krebs N. Overview of zinc absorption and extretion in the human gastrointestinal tract. J Nutr 2000;130:1374S-77S.

Kun JFJ, Missinou MA, Lell B, Sovric M, Knoop H, Bojowald B, Dangelmaier O, Kremsner PG. New emerging *Plasmodium falciparum* genotypes in children during the transition phase from asymptomatic parasitaemia to malaria. Am J Trop Med Hyg 2002;66:653-58.

Lanata CF, Black RE. Lot quality assurance sampling techniques in health surveys in developing countries: advantages and current constraints. World Health Stat Q 1991;44:133-39.

Lemeshow S, Taber S. Lot quality assurance sampling: single- and double-sampling plans. World Health Stat Q 1991;44:115-32.

Lengeler C. Insecticide-treated bednets and curtains for preventing malaria. Cochrane Database Syst Rev 2000;CD000363.

Lind T, Lonnerdal B, Stenlund H, et al. A community-based randomized controlled trial of iron and zinc supplementation in Indonesian infants: interactions between iron and zinc. Am J Clin Nutr 2003;77:883-90.

Lindenbaum J, Alam AK, Kent TH. Subclinical smallintestinal disease in East Pakistan. Br Med J 1966;2:1616-19.

Lindenbaum J. Tropical enteropathy. Gastroenterology 1973;64:637–652.

Liu YT, Old JM, Miles K, Fisher CA, Weatherall DJ, Clegg JB. Rapid detection of a-thalassaemia deletions and a-globin gene triplication by multiplex polymerase chain reactions. Br J Haematol 2000;108:295-99.

Logan RP, Walker MM. Epidemiology and diagnosis of *Helicobacter pylori* infection. BMJ 2001;323:920-22.

Lohman TG, Roche AF, Martorell R. Anthropometric standardization reference manual. Champaign, IL: Human Kinetics Books, 1988.

Lönnerdal B. Dietary factors influencing zinc absorption. J Nutr 2000;130:1378S-83S.

Lopez de Romana G, Gross R. IRIS. III: proposal of a multicenter efficacy study using a high-energy, micronutrient-dense spread. Food Nutr Bull 2003;24(3 Suppl):S39-41.

Lopriore C, Guidoum Y, Briend A, Branca F. Spread fortified with vitamins and minerals induces match-up growth and eradicates severe anemia in stunted refugee children aged 3-6 y. Am J Clin Nutr 2004;80:973-81.

Lunn PG. The impact of infection and nutrition on gut function and growth in childhood. Proceed Nutr Soc 2000;59:147-54.

Lunn PG, Northrop-Clewes CA, Downes RM. Intestinal permeability, mucosal injury, and growth faltering in Gambian infants. Lancet 1991a;338:907-10.

Lunn PG, Northrop-Clewes CA & Downes RM. Recent developments in the nutritional management of diarrhoea. 2. Chronic diarrhoea and malnutrition in The Gambia: studies on intestinal permeability. Trans R Soc Trop Med Hyg 1991b;85:8111.

Lusingu JPA, Vestergaard LS, Mmbando BP, et al. Malaria morbidity and immunity among residents of villages with different *Plasmodium falciparum* transmission intensity in North-Eastern Tanzania. Malaria Journal 2004;3:26.

Lynch SR, Stoltzfus RJ. Iron and ascorbic acid: proposed fortification levels and recommended iron compounds. J Nutr 2003;133:2978S-84S.

Mackinnon MJ, Mwangi TW, Snow RW, Marsh K, Williams TN. Heritability of Malaria in Africa. PLoS Med 2(12): e340.

Makler MT, Piper RC, Milhous WK. Lactate dehydrogenase and the diagnosis of malaria. Parasitol Today 1998;14:376-77.

Malaty HM, El-Kasabany A, Graham DY, et al. Age at acquisition of *Helicobacter pylori* infection: a follow-up study from infancy to adulthood. Lancet 2002;359:931-35.

Mason JB, Lofti M, Dalmiya N, et al. The micronutrient report: current progress and trends in the control of vitamin A, iodine, and iron deficiencies. Ottawa, Canada: The Micronutrient initiative, 2001.

Meulenbelt I, Droog S, Trommelen, GJM, Boomsma DI & Slagboom PE. High yield noninvasive human genomic DNA isolation method for genetic studies in geographically dispersed families and populations. Am J Hum Genet 1995;57:1252-54.

Miller WM. To Butterfly or To Needle: The Specimen Testing Question. Ann Intern Med 2004;141:645.

Mitchell HM, Lee A, Berkowicz J, Borody T. The use of serology to diagnose active *Campylobacter pylori* infection. Med J Aust 1988;149:604-09.

Mitchell H, Mégraud F. Epidemiology and diagnosis of *Helicobacter pylori* infection. Helicobacter 2002;7(Suppl. 1):8-16.

Mockenhaupt FP, Hamann L, Von Gaertner C, et al. Common polymorphisms of toll-like receptors 4 and 9 are associated with the clinical manifestation of malaria during pregnancy. J Infect Dis 2006;194:184-88.

Moher D, Schulz KF, Altman DG, for the CONSORT Group. The CONSORT statement: revised recommendations for improving the quality of reports of parallel-group randomised trials. Lancet 2001;357:1191-94.

Moody A. Rapid diagnostic tests for malaria parasites. Clin Microbiol Rev 2002;15:66-78.

Moran JR, Lewis JC. The effects of zinc deficiency on intestinal permeability: an ultrastructural study. Pediatr Res 1985;19:968-73.

Müller O, Becher H, Baltussen van Zweeden A, et al. Effect of zinc supplementation on malaria and other causes of morbidity in west African children: randomised double blind controlled trial. Brit Med J 2001;322:1567-70.

Müller O, Garenne M, Reitmaier P, Baltussen van Zweeden A, Kouyate B, Becher H. Effect of zinc supplementation on growth in West African children: a randomized double-blind placebo-controlled trial in rural Burkina Faso. Int J Epidemiol 2003;32:1098-1102.

Mutabingwa TK, Malle LN, De Geus A, Wernsdorfer WH. Malaria in infants whose mothers received chemoprophylaxis: response to amodiaquine therapy. Trop Geogr Med 1992;44:293-98.

Mutabingwa T, Nzila A, Mberu E, et al. Chlorproguanil-dapsone for treatment of drug-resistant falciparum malaria in Tanzania. Lancet 2001;358:1218-23.

Mwaniki D, et al. Anaemia and status of iron, vitamin A and zinc in Kenya Nairobi: UNICEF Kenya Country Office, 2002.

Näätänen R, Tervaniemi M, Sussman E, et al. Primitive intelligence in the auditory cortex. Trends Neurosci 2001;24:283-88.

Nestel P, Briend A, De Benoist B, et al. Complementary food supplements to achieve micronutriënt adequacy for infants and young children. J Pediatr Gastroenterol Nutr 2003;36:316-28.

Nestel P, Alnwick D. Iron/multimicronutrient supplements for young children: summary and conclusions of a consultation held at UNICEF,Copenhagen, August 19-20, 1996. Washington DC: International Nutritional Anemia Consultative Group, 1997.

O'Brien KO, Zavaleta N, Caulfield LE, Wen J, Abrams SA. Prenatal iron supplements impair zinc absorption in pregnant Peruvian women. J Nutr 2000;130:2251-55.

Ofori MF, Dodoo D, Staalsoe T, et al. Malaria-induced acquisition of antibodies to Plasmodium falciparum variant surface antigens. Infect Immun 2002, 70:2982-88.

Ojwang PJ, Ogada T, Gonzalez-Redondo JM, Kutlar A, Kutlar F, Huisman TH. beta S-haplotypes and alpha-thalassemia along the coastal belt of Kenya. East Afr Med J 1989; 66: 377-80.

Oppenheimer SJ. Iron and its relation to immunity and infectious disease. J Nutr 2001;131:616S-35S.

Osendarp S. Zinc supplementation in Bangladeshi women and infants: effects on pregnancy outcome, infant growth, morbidity and immune response. PhD thesis. Wageningen, The Netherlands: Wageningen University, 2001.

Piper R, Lebras J, Wentworth L, Hunt-Cooke A, Houzé S, Chiodini P, Makler M. Immunocapture diagnostic assays for malaria using Plasmodium lactate dehydrogenase (pLDH). Am J Trop Med Hyg 1999;60:109-18.

Prasad AS. Effects of zinc deficiency on Th1 and Th2 cytokine shifts. J Infect Dis 2000;182(Suppl):S62-S68.

Raab GM, Day S, Sales J. How to select covariates to include in the analysis of a clinical trial. Control Clin Trials 2000;21:330-342.

Rahman MM, Vermund SH, Wahed MA, et al. Simultaneous zinc and vitamin A supplementation in Bangladeshi children: randomised double blind controlled trial. BMJ 2001;323:314-18.

Regan D. Human brain electrophysiology. New York: Elsevier, 1989.

Roma-Giannikou E, Shcherbakov PL. *Helicobacter pylori* infection in pediatrics. Helicobacter 2002;7(Suppl. 1):50-55.

Ronaghy HA, Reinhold JG, Mahloudji M, Ghavami P, Fox MR, Halsted JA. Zinc supplementation of malnourished schoolboys in Iran: increased growth and other effects. Am J Clin Nutr 1974;27:112-21.

Ronaghy H, Spivey Fox MR, Garn SM, Israel H, Harp A, Moe PG, Halsted JA. Controlled zinc supplementation for malnourished school boys: a pilot experiment. Am J Clin Nutr 1969;22:1279-89.

Roper C, Richardson W, Elhassan IM, et al. Seasonal changes in the Plasmodium falciparum population in individuals and their relationship to clinical malaria: a longitudinal study in a Sudanese village. Parasitology 1998;116:501-10.

Rothenbacher D, Inceoglu J, Bode G, Brenner H. Acquisition of *Helicobacter pylori* infection in a high-risk population occurs within the first 2 years of life. J Pediatr 2000;136:744-48.

Rønn AM, Msangeni HA, Mhina J, Wernsdorfer WH, Bygbjerg IC. High level of resistance of *Plasmodium falciparum* to sulfadoxine-pyrimethamine in children in Tanzania. Trans R Soc Trop Med Hyg 1996;90:179-81.

Roy SK, Behren RH, Haider R, Akramuzzaman SM, Mahalanabis D, Wahed MA. Impact of zinc supplementation on intestinal permeability in Bangladeshi children with acute diarrhoea and persistent diarrhoea syndrome. J Peadiatr Gastroenterol Nutr 1992;15:289-96.

Roy SK, Tomkins AM, Haider R, et al. Impact of zinc supplementation on subsequent growth and morbidity in Bangladeshi children with acute diarrhoea. Eur J Clin Nutr 1999;53:529-34.

Sandstead HH. Improving study design. Am J Clin Nutr 1999;70:110-11.

Sandstead HH, Penland JG, Alcock NW, et al. Effects of repletion with zinc and other micronutrients on neuropsychologic performance and growth of Chinese children. Am J Clin Nutr 1998;68(suppl):470S–75S.

Sandstrom B, Lonnerdal B. Promoters and antagonists of zinc absorption. In: Zinc in human biology (Mills CF, ed.). New York, NY: Springer Verlag , 1989:57-78.

Sazawal S, Bentley M, Black RE, Dhingra P, George S, Bhan MK. **Effect of zinc supplementation on observed activity in low socioeconomic Indian preschool children.** Pediatrics 1996;98:1132-37.

Sazawal S, Jalla S, Mazumder S, Sinha A, Black RE, Bhan MK. Effect of zinc supplementation on cell-mediated immunity and lymphocyte subsets in preschool children. Indian Pediatr 1997;34:589-97.

Shah VS, Taddio A, Bennett S, Speidel BD. Neonatal pain response to heel stick vs venepuncture for routine blood sampling. Arch Dis Child 1997;77:F143-44.

Shankar AH. Nutritional modulation of malaria morbidity and mortality. Am J Trop Med Hyg 2000;62:663-69.

Shankar AH, Genton B, Baisor M, et al. The influence of zinc supplementation on morbidity due to *Plasmodium falciparum*: a randomized trial in preschool children in Papua New Guinea. Am J Trop Med Hyg 2000;62:663-69.

Shankar AH, Prassad AS. Zinc and immune function: the biological basis of altered resistance to infection. Am J Clin Nutr 1998;68:447S-63S.

Sharquie KE, Najim RA, Farjou IB, Al Timimi DJ. Oral zinc sulphate in the treatment of acute cutaneous leishmaniasis. Clin Exp Dermatol 2001;26:21-26.

Shann F, Hart K, Thomas D. Acute lower respiratory tract infections in children: possible criteria for selection of patients for antibiotic therapy and hospital admission. Bull World Health Organ 1984;62:749-53.

Sheu BS, Wu JJ, Lo CY, et al. Impact of supplement with Lactobacillus and Bifidum-containing yoghurt on triple therapy for *Helicobacter pylori* eradication. Aliment Pharmacol Ther 2002;16:1669-75.

Smith PG, Morrow RH. Methods for field trials of interventions against tropical diseases: a toolbox. UNDP/World Bank/WHO Special Programme for Research and Training in Tropical Diseases. Oxford: Oxford University Press, 2001:54-56.

Smuts CM, Benade AJ, Berger J, et al. IRIS. I: a FOODlet-based multiple-micronutrient intervention in 6- to 12-month-old infants at high risk of micronutrient malnutrition in four contrasting populations: description of a multicenter field trial. Food Nutr Bull 2003;24:S27-33.

Smyth A, Carty H, Hart CA. Clinical predictors of hypoxaemia in children with pneumonia. Ann Trop Paediatr 1998;18:31-40.

Snow RW, Marsh K. New insights into the epidemiology of malaria relevant for disease control. Br Med Bull 1998;54: 293–309.

Solomons NW, Ruz M, Gibson RS. Single-nutrient interventions with zinc. Am J Clin Nutr 1999;70:111-12.

Sommer A. Moving from science to public health programs: lessons from vitamin A. Am J Clin Nutr 1998;68(Suppl):513S-16S.

Stolzenberg-Solomon RZ, Blaser MJ, Limburg PJ, et al. *Helicobacter pylori* seropositivity as a risk factor for pancreatic cancer. J Natl. Cancer Inst 2001;93:937-41.

Sullivan PB, Marsh MN, Mirakian R, Hill SM, Milla PJ, Neale G. Chronic diarrhoea and malnutrition – histology of the small intestinal mucosal lesion. J Pediatr Gastroenterol Nutr 1991;12:195-203.

Takeda A. Zinc homeostasis and functions of zinc in the brain. Biometals 2001;14:343-51.

Umeta M, West CE, Haidar J, Deurenberg P, Hautvast JG. Zinc supplementation and stunted infants in Ethiopia: a randomised controlled trial. Lancet 2000;355:2021-26.

UN. How to weigh and measure children: assessing the nutritional status of young children in household surveys. New York, NY: United Nations, Department of Technical Cooperation for Development and Statistical Office, National Household Survey Capability Programme, 1986.

UN. Report of the Ad Hoc Committee of the Whole of the twenty-seventh special session of the General Assembly. General Assembly Official Records, Twenty-seventh special session, Supplement No. 3 (A/S-27/19/Rev.1). New York:United Nations, 2002.

Underwood BA. From research to reality: the micronutrient story. J Nutr 1998;128:145-51.

Usen S, Weber M, Mulholland K, Jaffar S, Oparaugo A, Omosigho C, Adegbola R, Greenwood B. Clinical predictors of hypoxaemia in Gambian children with acute lower respiratory tract infection: prospective cohort study. BMJ 1999;318:86-91.

Van de Vijver AJR, Willemse GR. Are reaction time tasks better suited for cultural minorities than paper pencil tests? In: Contemorary issues in cross-cultural psychology (Bleichrodt N, Drenth PJ, eds.). Lisse, The Netherlands: Swets and Zeitlinger, 1991:450-64.

Van de Vijver FJ, Jongmans MJ. Learning potential from a cross-cultural perspective: problems and perspectives. In: Learning potential assessment and cognitive training: actual research and perspectives in theory building and methodology, Volume 7. (Van der Aalsvoort GM, Resing W, Ruijssenaars AJ, eds.). Oxford, UK: Elsevier, 2002:211-26.

Verhoef H, West CE. Dietary factors that determine bioavailability of micronutrients: targets for crop breeding ? [Submitted for publication.]

Verhoef H, West CE, Bleichrodt N, Dekker PH, Born MP. Effects of micronutrients during pregnancy and early infancy on mental and psychomotor development. In: Micronutrient deficiencies in the first months of life. Nestlé Nutrition Workshop Series Pediatric Program, Volume 52. Vevey, Switzerland: Nestec Ltd/Basel, Switzerland: S Karger AG, 2003:327-57.

Verhoef H, West CE, Kraaijenhagen R, et al. Malarial anemia leads to adequately increased erythropoiesis in asymptomatic Kenyan children. Blood 2002a;100:3489-94.

Verhoef H, West CE, Nzyuko SM, et al. Intermittent administration of iron and sulfadoxine-pyrimethamine to control anaemia in Kenyan children: a randomised controlled trial. Lancet 2002b;360:908-14.

Wellinghausen N, Kirchner H, Rink L. The immunobiology of zinc. Immunol Today 1997;11:519-21.

WHO. Adaptation guide: a guide to indentifying necessary adaptations of clinical policies and guidelines, and to adapting the charts and modules for the WHO/UNICEF course. C: Technical basis for adapting clinical guidelines, feeding recommendations, and local terms, working draft version 4. Geneva, Switzerland: World Health Organization, Department of Child and Adolescent Health and Development, 1998.

WHO. Management of the child with a serious infection or severe malnutrition: guidelines for care at the first-referral level in developing countries. Reference no. WHO/FCH/CAH/00.1. Geneva, Switzerland: World Health Organization, Department of Child and Adolescent Health and Development, 2000a.

WHO. New Perspectives: malaria diagnosis. Report of a joint WHO/USAID informal consultation (25–27 October 1999). Reference no. WHO/CDS/RBM/2000.14. Geneva, Switzerland: World Health Organization, 2000b.

WHO/FAO. Human vitamin and mineral requirements. Report of a joint FAO/WHO Expert consultation (Bangkok, Thailand). Geneva, Switzerland: World Health Organization, 2002.

Wittes J. Sample size calculations for randomized controlled trials. Epid Rev 2002;24:39-53.

WMA. World Medical Association declaration of Helsinki. Ethical principles for medical research involving human subjects. World Medical Association, 2001. Available from <http://www.wma.net/e/policy/17-c_e.html>

Yacoob J, Jafri W, Abid S. *Helicobacter pylori* infection and micronutriënt deficiencies. World J Gastroenterol 2003;9:2137-39.

Yuzbasiyan-Gurkan V, Grider A, Nostrant T, Cousins RJ, Brewer GJ. Treatment of Wilson’s disease with zinc: X. Intestinal metallothionein induction. J Lab Clin Med 1992;120:380-86.

Zlotkin S, Arthur P, Schauer C, Antwi KY, Yeung G, Piekarz A. Home-fortification with iron and zinc sprinkles or iron sprinkles alone successfully treats anemia in infants and young children. J Nutr 2003;133:1075-80.

Annex 1.


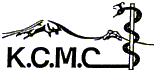


**Informed Consent Form**

Field coordinator: Dr Hans Verhoef

Name of the organization: Kilimanjaro Christian Medical Centre

Name of the sponsor: UN Children’s Fund/Netherlands Foundation for the Advancement of Tropical Research (NWO/WOTRO)/Wageningen University, The Netherlands

**Information Sheet for parents and relatives of children participating in the Research Project:**

**“**Effects of supplementation with zinc and other micronutrients on the health, development and well being of African children (Tanzania)”

**(Draft version)**

We conduct health studies for the Kilimanjaro Christian Medical Centre in Moshi. We are here to discuss a study on nutrition and malaria in children.

**Purpose of the study:** Food contains nutrients that our bodies need to function properly. Nutrients are taken up by the intestine, and end up in the blood. Once the nutrients are in the blood, they are transported to all parts of the body. Nutrients are needed to fight diseases, for body growth, and for the brain to function properly. The quality of food is very important: poor food quality means poor nutrition, and poor nutrition means poor health.

Some nutrients occur in foods in tiny amounts, but these nutrients are very important. Because they occur in such small amounts, we call them micronutrients. We are concerned that the types of foods used in Tanzania do not provide sufficient micronutrients. If young children would be given more micronutrients, their bodies might become stronger to fight diseases such as malaria. We also think that they may learn better from their environment. However, it could also be that foods eaten by children in this area already contain sufficient micronutrients. In that case, increasing the intake of micronutrients would have no effect on health.

The reason for doing this research is to find out if children become healthier when given extra micronutrients. For this reason, we will provide micronutrients in different combinations. We hope that the blood of the children consuming micronutrients will contain more of these nutrients. We also hope that children taking these micronutrients will have less malaria, and that they would become a little smarter because they learn better from their environment.

**Information about the micronutrients:** The micronutrients that we use in our study occur naturally in food. However, we think that the foods used in Tanzania does not contain sufficient of these micronutrients. There have been studies in other countries to examine the effects of micronutrients. In some of these studies, there was no effect at all. In other studies, however, children had less diarrhoea, and less pneumonia. Children in other countries often eat other food stuffs, and this could determine whether or not extra micronutrients are beneficial to health. We do not expect the micronutrients in our study to cause any harm, but we will nevertheless check for side effects that may occur.

**Procedures:** To find answers to some of our questions, we invite you to take part in this research project. We will divide children into 8 groups in such a way that each child has an equal chance of being in any of the groups (as in drawing lots, for example). Six out of eight groups will get the micronutrients that we are studying, and two out of eight groups will get an inactive substance. So each child has a three in four chance of receiving the micronutrients.

To help us know the true effect of the medicine, neither the doctors and scientists working on this research project nor the nurses will know who is in which group until the end of the research. This does not mean that the persons in the clinic looking after the participants will not know the condition of the participants’ disease during this period. Indeed, they will keep a very close watch on the participants.

**Why should we use an inactive medicine or a control medicine?** To find out if the micronutrients in our study work, we must compare children taking extra micronutrients with children who do not. All children in the study will daily receive a tablet.[[1]](#footnote-2) Most children receive tablets that contain micronutrients. Some children receive tablets that do not contain micronutrients.

If you choose to participate, we will collect blood from your child. In the first visit to the clinic, we will give a medical examination, and we will collect an amount of blood *[tube will be shown]* from a vein in the arm. The blood will be tested for the amount of nutrients, for the presence of infections, and for the presence of substances that help your body to fight infections. In these visits, you will also be asked to bring stool and urine samples that we will examine for worm infections.

A community volunteer will then daily provide a tablet to each child, although*,* as explained earlier, neither you nor we will know whether the tablets contain micronutrients or not. Each child will receive the same type of tablets for a period of 6 months. For this purpose, the children will be divided into groups that are indicated by different colours. It is very important that each child stays in the same group throughout the trial, and that children take their tablets daily, as scheduled.

During the study, we would ask you to bring the child every month for a medical examination, and we will collect a small amount of blood *[0.5 mL will be indicated on a tube]* by finger prick to test for the presence of malaria and for the amount of nutrients. At the end of the study, we will do the same measurements as at the start of the study. This means that we will again collect blood *[tube will be shown]* from a vein in the arm.

**What will happen if your child gets sick?** We would ask that immediately after the start of the illness, or any time that you are concerned about your child, you bring him or her to the research clinic. It is very important that you come us, and not to other clinics or hospitals, because we need to find out how often your child gets sick, and about the cause of the illness. We will medically examine your child, and do laboratory tests as needed. The first time that we find that your child has malaria, we will collect extra blood blood *[tube will be shown]* from a vein in the arm. We need this blood to test for the amount of nutrients, and for the presence of substances that help your body to fight infections.

Any child participating and their siblings will be treated free of charge and immediately for common childhood diseases occurring during the intervention period. Treatment will be given according to guidelines from the Tanzanian Ministry of Health. In the unlikely case that we find that this anti-malaria medicine does not have the desired effect, or not to the extent that we wish it to have, we will use another medicine.[[2]](#footnote-3)

To summarise, the duration of the study is 6 months, and you will be required to come to the research clinic monthly, and any time your child is ill. Each visit will take about two hours, except for the first and the last visit, which may take a large part of a day. We will collect blood every two months by finger punctures during the intervention period of 6 months. We will collect blood from a vein in the arm two times, or maximally three times: at the start and at the end of the 6-month intervention period, and the first time that we think that the child has malaria.

The test for the amount of nutrients in blood, and for the presence of substances that help your body to fight infections, can only be done in specialised laboratories. For this reason, part of the blood that is collected will be stored for future testing. This blood will be used for the purposes of this research only. Any blood remaining will be destroyed when the research is completed.

**Side effects:** As mentioned above, we do not expect any side effects from the micronutrients. However, we will follow each child closely. In the unlikely case that side effects occur, we may stop the use of the tablets. We will discuss with you, if this is necessary, and you will always be consulted before we move to the next step*.*

**Risks and discomforts:** By participating in this research, the child is likely to experience some discomfort from the blood collection, particularly at the beginning and the end of the study when we will be collecting blood from the veins in the arm. We will use especially fine needles and lancets in the hope that this will minimize discomfort. The amount of blood collected is small compared to the total amount in the body and we do not expect that the blood collection is harmful.

**Benefits:** If you participate in this research, you are likely to have the following benefits: common illnesses in your child or his/her siblings will be treated at no charge to you. If your child falls sick during this period he/she will be treated free of charge. For the duration of the study, your community will benefit from the presence of our research clinic. Your participation is likely to help us find the answer to the research question whether extra micronutrients are important for the health and development of African children. Future generations are likely to benefit.

**Incentives:** You will not be provided any incentive to take part in this research.

**Confidentiality:** The information that we collect about your child will be kept by the research team and will not be shared with anyone else.

**Right to refuse or withdraw:** You do not have to take part in this research if you do not wish to do so. You may stop participating in the research at any time that you wish without losing any of your rights as a patient.

**Who to contact:** If you have any questions you may ask them now or later. If you wish to ask questions later, you may contact any of the following: *[name of a local person, address/telephone number/e-mail]*[[3]](#footnote-4)

This proposal has been reviewed and approved by the Scientific and Ethical Review Committees of KCMC and the National Institute for Medical Research. The task of these committees is to make sure that research participants are protected from harm. If you wish to find about more about the Scientific and Ethical Review Committees, contact Dr Frank Mosha, Kilimanjaro Christian Medical Centre, Moshi, telephone *[to be provided].*

**Certificate of Consent**

I have been invited to take part in the research on etc. I have been told the purpose of this research study is to examine the effect of micronutrients on the health and development of children, particularly with respect to malaria. I understand that a community volunteer will daily give my child a tablet that may or may not contain micronutrients. I understand that blood will be collected from my child monthly, and when malaria is suspected. I have been told that there no risks are expected for my child, although the monthly blood collection may cause discomfort. I understand that I have the right to withdraw from the study at any time without in any way affecting the medical care of myself, my child or my family as a patient. My child or his/her siblings will be treated free of charge for common illnesses that may occur during the study. For the duration of the study, my community will benefit from the presence of our research clinic. I will not be provided any incentive to take part in this research. Information that will be collected about my child will be kept by the research team and will not be shared with anyone else. If I wish to ask questions later, I can contact any of the following: *[name of a local person, address/telephone number/e-mail]*[[4]](#footnote-5)

I have read the foregoing information, or it has been read to me. I have had the opportunity to ask questions about it and any questions that I have asked have been answered to my satisfaction. I consent voluntarily that my child will participate in this study.

Name: Date and signature:

___________________________ ___________________________

___/___/___ (*dd/mm/yy*)

***If illiterate***

Name of independent literate witness: Date and signature of witness:

___________________________ ___________________________

___/___/___ (*dd/mm/yy*)

Name of researcher: Date and Signature of Researcher:

Dr Hans Verhoef
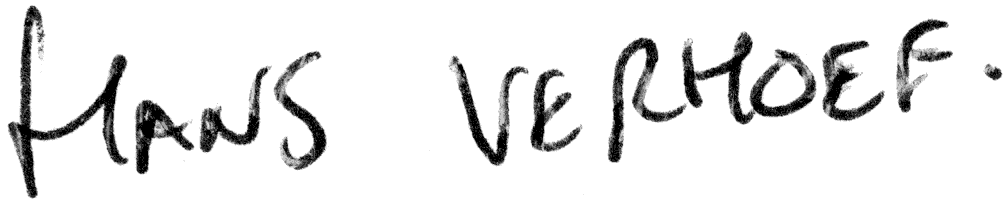


___/___/___ (*dd/mm/yy*)


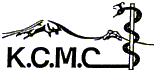


**Fomu ya ridhaa ya kushiriki katika utafiti**

**(informed consent form**)

**Mkuu wa Mradi:** Dr Hans Verhoef

**Taasisi:** Hospitali ya Kikristo ya Kilimanjaro (KCMC), Moshi

**Mfadhili:** Mfuko wa Umoja wa mataifa wa huduma kwa watoto (UNCF)/Mfuko wa Maendeleo ya Utafiti wa Kitropiki wa Uholanzi (NWO/WOTRO)/Chuo kikuu cha Wageningen, Uholanzi

**Fomu ya taarifa ya wazazi wanaoshiriki kwenye mradi wa utafiti:**

“Matokeo ya viasili/madini/virutubisho aina ya Zinc na jamii yake katika afya ya watoto wa Tanzania“.

Utafiti huu wa kiafya utafanyika chini ya usimamizi wa hospitali ya KCMC ya mjini Moshi. Hapa tunaelezea faida ya lishe kwa watoto katika kujikinga na maradhi hasa Malaria.

**Lengo la Utafiti**

Katika chakula tulacho kila siku, tunapata virutubisho mbalimbli ambavyo hufika sehemu mmbalimbali za mwili kwa njia ya mfumo wa damu. Mwili huhitaji virutubisho mbalimbali vilivyomo ndani ya chakula kwa ukuaji, kujikinga na maradhi, na pia kuwezesha akili kufanya kazi yake sawasawa. Ubora wa chakula ni muhimu sana. Chakula duni husababisha lishe duni ambayo husababisha afya mbaya.

Baadhi ya virutubisho hupatikana kidogo sana katika chakula tulacho lakini huhitajika sana mwilini na kazi yake ni kubwa. Virutubisho hivi huitwa micronutrients. Kwa bahati mbaya vyakula vingi tunavyokula hasa katika sehemu mbalimbali za nchi yetu vina vurutubisho vidogo sana kukudhi mahitaji ya mwili. Hivyo kama vyakula tunavyokula vingekuwa na virutubisho vya kutosha, hasa vile tunavyowalisha watoto wetu, vingeipa miili ya watoto hawa uwezo wakupambana na magonjwa kama malaria ambayo ni moja ya magonjwa yanayoua watoto wengi wa Tanzania na Afrika kwa jumla. Pia chakula bora humuwezesha mtoto kujifunza na kupambana na mazingira yanayomzunguka. Pia inawezekana kuwa watoto hawa wanweza kula vyakula vyenye virutubisho vyakutosha ingawa si wote, kiasi kwamba hata kuongeza kiasi kidogo katika milo yao hakuwezi kuathiri chochote katika ukuaji wa mtoto.

Hivyo basi, lengo la utafiti huu ni kuangalia mabadiliko ya kiafya wakati mtoto anapopewa virutubisho zaidi katika chakula chake. Katika utafiti huu watoto watapewa virutubisho hivi katika michanganyiko mbalimbali. Mategemeo ni kwamba watoto wanaopata virutubisho hivi katika chakula watapata nguvu ya kujikinga na ugonjwa hatari wa malaria na kuimarisha akili na uwezo wa kufikiri na kujifunza vizuri zaidi juu ya mazingira yanayowazunguka.

**Taarifa kuhusu virutubisho**

Virututubisho vitakavyotumika katika utafiti huu ni vile ambavyo vinapatikana katika vyakula asilia tulavyo kila siku. Hata hivyo, tunafikiri kwamba vyakula hivyo havina virutubisho vya kutosha kama ilivyogundulika katika tafiti mbalimbali zilizofanyika katika nchi nyingine duniani, ingawa matokeo ya tafiti mbalimbali yanaonyesha kutofautiana. Hata hivyo utafiti unaonyesha kwamba watoto waliopewa virutubisho hivi, walipata kinga dhidi ya magonjwa kama kuhara na nyumonia (pneumonia). Tunategemea kuwa virutubisho vitakavyotumika katika utafiti huu havina athari kwa washiriki katika utafiti huu (subjects). Hata hivyo kazi mojawapo itakuwa ni kuangalia pia athari zozote zinazoweza kujitokeza kutokana na virutubisho hivyo kakatika kipindi cha utafiti huu.

**Utaratibu**

Ili kutimiza lengo la utafiti huu tuna waalika nyote kushiriki kama wadau wakuu katika utafiti huu. Watoto watakaoshiriki katika utafiti huu watagawanywa katika makundi 8 kiasi kwamba kila mtoto atakuwa na nafasi sawa ya kuwa kwenye kundi lolote kati ya hayo nane. Makundi sita kati ya nane watapewa micrinutrients (virutubisho/madini) tunayofanyia utafiti, na makundi mawili kati ya hayo nane watapata placebo (control) kiasi kwamba kila mtoto atakuwa na nafasi tatu kati ya nne ya kupata virutubisho hivyo.

Ili kupata matokeo mazuri ya utafiti huu, si manesi wala madaktari na watafiti katika mradi huu watakaojua kundi lipi limepewa nini mpaka mwisho wa utafiti huu. Hii haimaanishi kwamba waangalizi wa watoto katika kliniki husika hawatajua maendeleo ya kiafya ya washiriki katika utafiti huu bali watakuwa karibu na kuangalia maendeleo ya watoto hawa kwa karibu kabisa.

**Kwa nini tutumie control Medicine?**

Ili kutambua kwamba virutubisho tunavyotumia katika utafiti wetu vinafanya kazi, ni lazima kulinganisha kati ya kundi la watoto wanaopata virutubisho vya ziada na lile ambalo halipati kabisa. Wototo wote watapata vidonge vyenye virutubisho hivi. Wengi wa watoto hawa watapata vidonge vyenye virutubisho. Wachache watapata vidonge visivyo na virutubisho hivyo.

Kama utaamua kushiriki katika utafiti huu, tutachukua damu toka kwa mtoto wako. Siku ya kwanza ya kufika kliniki tutachunguza afya ya mtoto, na tutachukua kiasi cha damu (Neli ya ujazo wa mililita 7 itaonyeshwa) kutoka katika mshipa wa damu wa aina ya vena wa mkono wa mtoto. Damu hii itachunguzwa kuona kiasi cha virutubisho vilivyomo, ugonjwa wowote kama upo, na uwepo wa kinga za mwili kupambana na magonjwa mbalimbali. Pia tutaomba ulete kiasi cha haja kubwa (kinyesi cha mtoto) na mkojo (haja ndogo) kwa uchunguzi wa magonjwa kama minyoo n.k.

Mwanajamii wa kujitolea atakuwa hapo kila siku kutoa vidonge hivyo kwa kila mtoto ingawa hatajua mtoto yupi amepata vidonge vipi kati ya vyenye virutubisho au la! Kila mtoto atapata aina ile ile ya kidonge kwa muda muda wa miezi sita mfululizo. Ili kutimiza lengo hili basi, watoto watagawanywa katika makundi ambayo yataonyeshwa na tofauti ya rangi za vidonge vitakavyotolewa. Ni muhimu kwamba kila mtoto atabaki katika kundi lilelile mpaka mwisho wa utafiti na kwamba watoto watapata na kutmia vidonge hivyo kila siku kama ratiba itakavyokuwa.

Tungependa mzazi ama mlezi kumleta mtoto wako kwa uchunguzi wa afya yake kila mwezi kwa muda wote wa utafiti huu, na tutachukua damu kidogo (ujazo wa mililita 0.5 utaonyeshwa katika neli) kutoka kwenye kidole cha mtoto kwa uchunguzi ili kuona kama ana malaria au la na pia kuangalia kiasi cha virutubisho vilivyomo. Baada ya muda wa wiki ishirini tutachukua vipimo pia kama tulivyofanya mwanzoni mwa utafiti. Hii ina maana kuwa tutachukua damu tena (neli ya ujazo wa mililita 7 itaonyeshwa) kotoka katika mshipa wa damu wa vena wa mkononi wa mtoto.

**Nini kitafanyika endapo mtoto wako ataugua wakati utafiti unaendelea?**

Tungeshauri na kusistiza kumleta mtoto wako kliniki kwa uchunguzi mara mtoto wako anapopata homa ama unapofikiri kuwa mtoto wako anahitaji kuchunguzwa. Pia tunasistiza kuwa ni vema na ni muhimu kumleta mtoto wako kwetu punde inapohitajika na si kliniki ama hospitali nyingine, kwani tunahitaji kujua pia ni mara ngapi mtoto wako anaugua na sababu za ugonjwa. Tutamchunguza mtoto wako na kumpima kwa kadiri itakavyohitajika tatika maabara yetu. Mara tutakapogundua kwa mara ya kwanza kuwa mtoto wako ana malaria, tutachukua damu toka kwenye vena ya mkono wako (neli ya ujazo wa mililita 7 itaonyeshwa). Damu hii itahitajika kwa ajili ya kuangalia kiasi cha madini na virutubisho vingine, na pia kama kuna viini vinavyoasaidia mwili kupambana na magonjwa.

Watoto wote wanoshiriki kwenye utafiti na wadogo zao watatibiwa bure kwa magonjwa yote ya kawaida yanayowaathiri watoto katika kipindi hicho cha miezi sita cha utafiti. Matibabu yatatolewa kwa mujibu wa muongozo toka Wizara ya Afya ya Tanzania. Ili kutibu malaria, tutatoa dawa (mchanganyiko wa artesunate na lumefantrin) ambazo ni nzuri na gharama kidogo ukilinganisha na dawa ambazo kwa kawaida hutumika kama vile fansida, ambazo pia zinashauriwa na Shirika la Afya Duniani (WHO). Endapo itatokea kwamba dawa hii haitafaa kutibia, tutabadilisha aina ya dawa kwa kadiri inavyoonekana kufaa kutibu mtoto kulingana na hali yake kiafya.

Kwa muhtasari, utafiti utafanyika kwa muda wa miezi kumi na miwili (12), na utatakiwa kuja kliniki kila mwezi, na muda wowote mtoto anapokuwa anaumwa. Kila wakati utakapomleta mtoto kliniki, itachukua muda wa masaa mawili (2) isipokuwa siku ya kwanza na siku ya mwisho ambazo zitachukua muda mrefu kidogo. Tutachukua damu kila miezi miwili kutoka kwenye kidole cha mtoto kwa muda wa miezi kumi na miwili (12). Tutachukua damu kutoka kwa mshipa wa damu wa mkononi mara mbili na si zaidi ya mara tatu: mwanzoni na mwishoni mwa utafiti, na mara ya kwanza mtoto anapokuwa anafikiriwa kuwa na malaria.

Uchunguzi wa kiasi cha virutubisho vilivyomo kwenye damu, na kuangalia uwepo wa viini vinavyousaidia mwili wake kupambana na magonjwa, utafanyika katika maabara maalumu tu. Kwa sababu hii, sehemu ya damu itahifadhiwa kwa kufanyiwa uchunuzi wa baadaye. Damu hii itatumika kwa uchunguzi kama inavyotaarifiwa katika utafiti huu tu na si vinginevyo. Damu inayobaki itatupwa katika sehemu maalumu za kuharibia uchafu mara utafiti utakapokuwa umefikia tamati.

**Athari:** Kama ilivyoarifiwa hapo juu, hatutegemei athari zozote kutokana na virutubisho na dawa tutakazotumia katika utafiti huu. Hata hivyo, tutafuatilia kila mtoto kwa karibu. Na kama itatokea kuwa na athari zozote, kitu ambacho hakitarajiwi sana, tutasimamisha matumizi ya vidonge hivyo. Tutajadiliana nawe, kama itabidi, na pia tutakuona kwa ushauri kabla ya kuendelea na hatua inayofuata.

**Yatokanayo na utafiti:**

Kwa kushiriki katika utafiti, inategemewa kuwa matoto atajisikia vibaya wakati wa kuchukua damu, hasa mwanzoni na mwishoni mwa utafiti wakati damu itakapochukuliwa kutoka kwenye mshipa wa damu wa mkononi. Tutatumia sindano nyembamba ambazo hazitasababisha maumivu kwa mtoto. Kiasi cha damu kitakachotolewa ni kidogo ukilinganisha na kiasi cha damu mwilini na hatutegemei kuwa itasababisha athari kwa mtoto.

**Faida:**

Kwa kushiriki katika utafiti huu, unatarajiwa kupata faida zifuatazo: Matibabu bure kwa watoto wako wote. Kama mtoto wako ataugua wakati wowote wa utafiti atatibiwa bure. Kwa kipindi hicho cha utafiti, jamii nzima itafaidika na kliniki yetu ya utafiti hapo. Ushiriki wenu utatusaidia kuona ni namna gani tutainua afya ya watoto wa Tanzania na kama inahitajika kuongeza kiasi fulani cha virutubisho kwenye chakula kwa afya na maendeleo ya mtoto wa Africa. Hii ni faida tarajali kwa vizazi vijavyo.

**Malipo:**

Hakutakuwa na malipo yoyote yatakayotolewa kwa kushiriki katika utafiti huu.

**Usiri:**

Taarifa itakayopatikana kuhusiana na mtoto wako itatuzwa na ni siri ya timu ya utafiti na hakuna mtu mwingine yeyote ataweza kuifikia na kuitumia.

**Haki ya kukataa ama kujitoa kwenye utafiti:**

Ni haki ya mtu kuchagua kushiriki ama kutoshiriki kwenye zoezi hili la utafiti bila kushurutishwa wala kulazimishwa. Na mtu atashiriki tu endapo ataamua mwenyewe kwa hiari yake. Pia ni haki ya mshiriki kujitoa kushiriki kwenye utafiti wakati wowote bila kupoteza haki zake kama mgonjwa ama mshiriki.

**Kwa maelezo zaidi:**

Kama una maswali unaweza kuuliza sasa au baadaye. Na kama unafikiria kuuliza maswali baadaye, unaweza kumuona yeyote kati ya wafuatao: (*Jina la mtu mwenyeji wa sehemu husika, anwani/namba ya simu/anwani ya barua pepe [email address]*)

Muongozo huu wa utafiti umepitiwa na kuthibitishwa na bodi ya kupitia na kuidhinisha utafiti (ethical Review Committees) ya KCMC na Taasisi ya Utafiti ya Taifa (NIMR). Kazi ya vyombo hivi ni kuhakikisha kuwa washiriki wa utafiti wanalindwa na hawapati madhara yoyote yatokanayo na utafiti. Kwa taarifa zaidi kuhusu vyombo hivi, vya utafiti, tafadhali muone ama mtafute Dr Frank Mosha, Hospitali ya Kikristu ya Kilimanjaro (KCMC), Moshi, nambari ya simu (itatolewa).

**CHETI CHA USHIRIKI WA HIARI (CERTIFICAT OF CONSENT)**

Nimealikwa kushiriki katika utafiti wa matokeo ya ulishaji wa virutubisho vya aina ya Zinc na jamii yake kwa watoto wa Tanzania. Nimeelezwa lengo la utafiti huu kwamba ni kuangalia matokeo ya ulishaji wa virutubisho hivyo katika kuimarisha afya na maendeleo ya watoto, hasa katika kupambana na ugonjwa hatari wa malaria. Naelewa kwamba kutakuwa na mwanajamii atakayejitolea kutoa kila siku, kwa mtoto wangu kidonge chenye virutubisho ama kisicho na virutubisho. Ninaelewa kwamba mtoto wangu atachukuliwa damu kila mwezi, na punde atakapohisiwa kuwa na malaria kwa uchnguzi. Nimeambiwa kuwa hakuna athari zinazotarajiwa kwa mtoto, ingawa utoaji wa damu wa kila mwezi utampunguzia starehe mtoto ingawa ni kwa muda mfupi. Ninaelewa kuwa ninayo haki ya kujitoa kushiriki kwenye utafiti wakati wowote bila kupoteza haki yangu ya kutibiwa, mtoto wangu, wala familia yangu kama wagonjwa. Mtoto wangu na wanaozaliwa pamoja watatibiwa bure kwa magonjwa ya kawaida yanaweza kujitokeza wakati wote wa utafiti. Kwa muda huo wa utafiti, jamii yangu itafaidika na uwepo wa kliniki ya utafiti. Sitapewa malipo ili kushiriki kwenye utafiti. Taarifa itakayokusanywa juu ya mtoto wangu itatuzwa na timu ya watafiti na hakuna mtu yeyote nje ya timu ataruhusiwa kuiona wala kuitumia. Kama nitataka kuuliza maswali baadaye, naweza kumuona yeyote kati ya wafuatao: (*Jina la mtu mwenyeji wa sehemu husika, anwani/namba ya simu/anwani ya barua pepe [email address]*)

Nimesoma taarifa hii, au nimesomewa taarifa hii. Nilikuwa na nafasi ya kuuliza maswali kuhusiana na kazi hii na swali lolote nililouliza lilijibiwa kiasi cha kuniridhisha. Ninakubali kwa hiari kwamba mtoto wangu atashiriki katika utafiti huu.

Jina: Tarehe na sahihi:

___________________________ ___________________________

___/___/___ (*siku/mwezi/mwaka*)

***Kama mzazi hajui kusoma wala kuandika***

Jina la shahidi huru: Tarehe na sahihi:

___________________________ ___________________________

___/___/___ (*siku/mwezi/mwaka*)

Jina la mtafiti: Tarehe na Sahihi ya mtafiti:

Dr Hans Verhoef
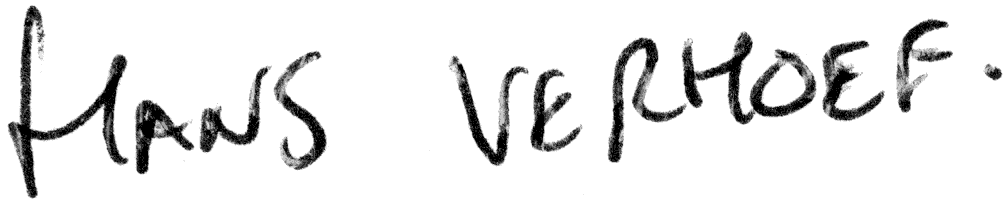


___/___/___ (*siku/mwezi/mwaka*)

^4^Itatolewa kwa ushauri ushirikiano na serikali ya mtaa na mtaalamu wa afya.

Annex 2. **Tentative composition of the supplements: justification**

**General considerations:**

1. For practical reasons, one dose of supplements will be used for each treatment group, regardless of age.
2. For vitamins, an overage will be taken into account to compensate for possible losses or degradation during storage due to environmental factors, and to insure that the target level will be minimally achieved. The overage estimates are based on the percentages determined by the Micronutrient Initiative.
3. For each micronutrient, the total daily intake (dose from supplement excluding overage, plus the dietary intake) should not exceed the Adequate Intake (AI) or Tolerable Upper Intake Level (UL) as defined by US Institute of Medicine (IOM)[[5]](#footnote-6) with the exception of zinc (see below). In addition, the contents of the micronutrients should be at levels that are below values known or suspected to inhibit the effect of supplemental zinc.
4. The supplement should supply at least 1 Recommended Daily Allowance (RDA)[[6]](#footnote-7) aged 1-3 years, in addition to the dietary supply. This will result in a higher total intake of micronutrients than recommended for US children, but takes into account that the micronutrients needs of children from poor, rural African families is likely to be higher than of their US peers for the following reasons. First, because of infections, African children may have an increase metabolic rate (e.g. loss of folate due to increased erythrocyte turn-over in malaria), or they may lose micronutrients through blood (e.g. iron loss due to hookworm), faeces (e.g. loss of trace elements during diarrhoea) and urine (e.g. loss of vitamin A during febrile episodes). Second, they may already be deficient in micronutrients at the onset of supplementation.

**Zinc:**

1. Zinc will be supplemented at 10 mg/day, with consideration that daily doses of 10-20 mg have been used repeatedly and successfully in many randomized controlled trials in children in the same age group without any adverse effects.[[7]](#footnote-8). A daily dose of 10 mg is higher than the ULs as determined by IOM. However, because of the lack of adequate data on zinc toxicity, these ULs may be inappropriately low (IZiNCG 2004). This is supported by the fact that, in a substantial proportion of US children aged 1-3 years, the actual intake of zinc exceeds the UL of 7 mg/day (median: 6.3 mg from the diet alone; 6.4 mg when also considering intake from supplements). Accordingly, IZiNCG only determined a no-observed-adverse-effect (NOAEL) level (6 mg/day, 8 mg/day and 14 mg/day in infants 7-12 mo, children 1-3 y and children 4-8 y, respectively). These NOAEL estimates incorporated an uncertainty factor of 1.5, assuming that zinc supplements may be consumed with a meal. In our study, we will advise parents to provide the supplements apart from meals, so that direct interference between supplemental zinc and dietary iron and copper is possibly avoided. When estimated without this correction factor, the NOAEL would have been 9 mg/day, 12 mg/day and 22 mg/day for infants 7-12 mo, children 1-3 y and children 4-8 y, respectively. IZiNCG concluded that further prospective studies of the possible adverse effects of varying levels of supplemental zinc are needed, including studies with higher doses than used in the Indonesian study.

**Micronutrients other than zinc:**

1. *Iron:* Supplementation at a level between 1 and 2 mg iron/kg body weight/day is appropriate to prevent anaemia and other adverse health effects associated with iron deficiency (INACG 1997). Assuming reference body weights of 7.7 kg, 9.3 kg, 11.5 kg, 16.5 kg and 15.7 kg for children aged 6-12 mo, 1-2 y, 2-3 y, 3-4 y and 4-5 y, respectively (these values are somewhat less than values from US children of similar age; <http://www.cdc.gov/growthcharts>), a supplementation level of 1.5 mg iron/kg body weight/day corresponds to 11.6 mg, 14.0 mg, 17.2 mg, 19.7 mg and 21.8 mg/day, respectively. In our study, the supplements will contain 18 mg/day, which would corresponds to a molar ratio of Fe:Zn of 2:1 when iron is supplemented in combination with zinc. There is no evidence of adverse effects at this level of iron supplementation on biochemical zinc status, either whether given alone or in combination with zinc.[[8]](#footnote-9) Even when absorption is doubled due to the high levels of vitamin C in the supplement (see below), the levels of supplemental iron would not exceed the AI or UL (both the AI for infants and the UL for children aged between 1 and 8 years are 40 mg/day; IOM 2001).
2. *Vitamin C:* For vitamin C, the RDA is estimated as the amount required to provide antioxidant protection (IOM 2000:139-145). Optimisation of iron absorption, however, requires higher levels of vitamin C. When taken with phytate-containing foods, the molar ratio of ascorbic acid to iron should be at least 4:1, corresponding to a weight ratio of 12.6:1.[[9]](#footnote-10) Ascorbic acid probably improves iron absorption even in the absence of phytate. We will provide vitamin C as purified L-ascorbic acid. [Note: iso-ascorbic acid or D-ascorbic acid (erythorbic acid) is a biological inactive vitamin C enantiomer. It has antioxidant capacity, and is used as an additive, but is not involved in biologically relevant redox (hydroxylation) reactions.]
3. *Iodine:* The supplements will provide 130 µg iodine, which corresponds to 1 RDA for infants but is higher than the RDA for children aged 1-5 years.
4. *Magnesium:* Magnesium will be supplemented at a level corresponding to the UL for children aged 1-8 y (65 mg). The estimate of this UL was based on the assumption that higher intake levels of magnesium from non-food sources (including various magnesium salts used for pharmacologic purposes) may result in very mild, reversible osmotic diarrhoea. The lack of any available data regarding the effects of magnesium supplements in infants makes it impossible to establish a specific UL for infants (IOM 1997). For data on bioavailability: see Ranade and Somberg (2001).[[10]](#footnote-11)
5. *Vitamin A:* The requirements for infants is higher than would be provided by our supplements (500 µg versus 400 µg), but many Tanzanian infants already receive high-dose vitamin A as part of the immunization campaign (e.g. in a survey conducted in 2000 in 4 divisions of Handeni and Korogwe Districts, 75% of children <5 y were reported to have taken vitamin A capsules in the previous 6 months; unpublished data, World Vision 2002).
6. *Calcium:* Although not a micronutrient, we decided to also include calcium in the supplement because of the intake of calcium in developing countries is generally low. The contents were conservatively set to provide 50 mg/day (for data on bioavailability of calcium carbonate: see Hanzlik et al. 2005).[[11]](#footnote-12) Although it appears unlikely that calcium per se has a negative effect on zinc absorption, we cannot exclude that the supplements are consumed shortly before or after a meal, whilst calcium may reduce zinc absorption from phytate-containing meals due to the formation of phytate-Ca-Zn complexes.[[12]](#footnote-13)
7. *Copper:* Copper will be provided as cupric sulphate. In most supplements, copper is provided as cupric oxide but there is strong evidence that this is not absorbed.[[13]](#footnote-14)
8. *Other micronutrients:* For all other micronutrients, the content of the supplements was set to supply daily at least one Recommended Daily Allowance (RDA)[[14]](#footnote-15) for children aged 1-3 years (at baseline of the randomized trial, approximately two-thirds of all children are expected to be within this age range). For infants, these levels are above the RDA but well below the AI or UL.

| **For none of the nutrients would the intake from supplements exceed the Adequate Intake or Tolerable Upper Intake Level for children within the age range in our study, even when assuming no losses due to environmental degradation.** |
| --- |

**Table. Tentative composition of the micronutrient supplements**

| Active substance | Target level (including overage) and form | **Infants 6-12 mo** | | **Children 1-3 y** | | **Children 4-5 y** | | **Upper Limit as determined by the US IOM** | | |
| --- | --- | --- | --- | --- | --- | --- | --- | --- | --- | --- |
|  |  | RDA  (IOM) | RNI  (FAO/WHO) | RDA  (IOM) | RNI (FAO/WHO) | RDA  (IOM) | RNI  (FAO/WHO) | Infants  6-12 mo | Children  1-3 y | Children  4-5 y |
| Vitamin A | 450 µg RAE all-*trans* retinyl acetate | 500 µg | 400 µg | 300 µg | 400 µg | 400 µg | 450 µg | 600 µg | 600 µg | 900 µg |
| Vitamin B_1_ | 0.75 mg as thiamin mononitrate | 0.3 mg | 0.3 mg | 0.5 mg | 0.5 mg | 0.6 mg | 0.6 mg | ND ^b^ | ND ^b^ | ND ^b^ |
| Vitamin B_2_ | 0.55 mg riboflavin | 0.4 mg | 0.4 mg | 0.5 mg | 0.5 mg | 0.6 mg | 0.6 mg | ND ^b^ | ND ^b^ | ND ^b^ |
| Niacin | 6.6 mg niacinamide | 4 NE* | 4 NE* | 6 NE* | 6 NE* | 8 NE* | 8 NE* | ND | 10 mg | 15 mg |
| Vitamin B_6_ | 0.58 mg pyridoxine | 0.3 mg | 0.3 mg | 0.5 mg | 0.5 mg | 0.6 mg | 0.6 mg | ND | 30 mg | 40 mg |
| Folate (vit. B_11_) | 187.5 µg folate equivalents | 80 µg | 80 µg | 150 µg | 160 µg | 200 µg | 200 µg | ND | 300 µg | 400 µg |
| Vitamin B_12_ | 1.2 µg cyanocobalamid | 0.5 µg | 0.5 µg | 0.9 µg | 0.9 µg | 1.2 µg | 1.2 µg | ND ^c^ | ND ^c^ | ND ^c^ |
| Vitamin C | 75 mg purified L-ascorbic acid | 50 mg | 30 mg | 15 mg | 30 mg | 25 mg | 30 mg | ND ^d^ | 400 mg | 650 mg |
| Vitamin D | 6.8 µg as vitamin D_3_ (cholecalciferol) | 5 µg | 5 µg | 5 µg | 5 µg | 5 µg | 5 µg | 25 µg | 50 µg | 50 µg |
| Vitamin E | 6.6 mg tocopherol equivalents ^a^ | 0.6 mg/kg BW | ND | 6 mg | ND | 7 mg | ND | ND | 200 mg | 300 mg |
| Vitamin K | 45 µg natural K vitamins | 2.5 µg | 10 µg | 30 µg | 15 µg | 55 µg | 20 µg | ND ^e^ | ND ^e^ | ND ^e^ |
| Zinc | 10 mg zinc sulphate (ZnSO_4_) |  |  |  |  |  |  |  |  |  |
| Iron | 18 mg as ferrous sulphate (FeSO_4_) | 11 mg | 18.6 mg | 7 mg | 11.6 mg | 10 mg | 12.6 mg | 40 mg | 40 mg | 40 mg |
| Iodine | 90 µg potassium iodide (KI) | 130 µg | 90 µg | 90 µg | 90 µg | 90 µg | 90 µg | ND ^f^ | 200 µg ^f^ | 300 µg ^f^ |
| Copper | 340 µg cupric sulphate (CuSO_4_) | 220 µg | ND | 340 µg | ND | 440 µg | ND | ND | 1 mg | 3 mg |
| Selenium | 20 µg sodium selenate (Na_2_SeO_4_) | 20 mg | 10 mg | 20 mg | 17 mg | 30 mg | 22 mg | 60 µg | 90 µg | 150 µg |
| Magnesium | 50 mg magnesium chloride (MgCl_2_) | 75 mg | 54 mg | 80 mg | 60 mg | 130 mg | 76 mg | ND | 65 mg ^g^ | 110 mg ^g^ |

ND: Not derived; BW: body weight; NE: 1 niacin equivalent = 60 mg of tryptophan = 1 mg of niacin

Overages: 50% (vitamins A, C and K); 35% (vitamin D); 30% vitamin B_12_; 25% (vitamin B1, folate); 15% (vitamin B_6_); 10% (vitamin B_2_, niacin, vitamin E); 0% (trace elements)

^a^ 1TE=1 mg RRR-α-tocopherol

^b^ Toxicity is not a problem (WHO/FAO 2000)

^c^ Therapeutic oral dose of 1 mg has never been reported to cause adverse effects (WHO/FAO 2002)

^d^ Upper limit according to WHO/FAO (2002): 1 g/day.

^e^ When taken orally, natural K vitamins seem free of toxic effects

^f^ Upper limits according to WHO/FAO (2002): 7-12 mo: 840 µg; 1-3 y: 750 µg; 4-8 y: 750 µg

^g^ Applicable to supplementary Mg only

Annex 3. **Tentative list of outcome indicators of cognitive and psychomotor development**

*Event-related potential (ERP) parameters* are electrophysiological measures used for detecting abnormal neurophysiological processes (Regan 1989). Very early ERPs are influenced mainly by physical characteristics of stimuli, and are used to study nervous pathways that transmit the incoming information to the sensory areas in the brain. ERPs in the range 100-500 ms are used to study the effects of arousal and attention on information processing. Auditory stimuli are particularly convenient in infants, because they do not require the subjects' understanding of the task and cooperation. A powerful way to study early memory and cognition is the auditory mismatch paradigm. A tone is presented repeatedly to create a memory of the current auditory environment. Every now and then, this tone is replaced by a slightly different, 'deviant' tone which leads to a specific response of the auditory cortex called 'mismatch negativity' (Näätänen et al. 2001, Heslenfeld 2003, Huizinga et al. 2002, Elton et al. 1997). This response has been shown to be independent of the attention of the subject and the physical characteristics of the deviant tone (Heslenfeld et al. 1996). To detect such a deviant tone in a sequence of standard tones, the neural system needs to have a memory for the standard tones, it needs to create an expectation about which tones are likely to occur, and it needs a mechanism to compare actual tones with expected ones. As the mismatch paradigm measures these functions even without subjects' intention to participate, this paradigm is ideally suited to study memory and cognition in very young children (Cheour et al. 2000). Additionally, standard neurophysiological indices such as N100 amplitude and latency can be derived easily from ERPs evoked by both tones.

Annex 4. **Budget (in euro)**

| **No** | **Category** | **Item** | **Justification** | **Year 1** | **Year 2** | **Year 3** | **Year 4** | **Total** |
| --- | --- | --- | --- | --- | --- | --- | --- | --- |
| 1 | Personnel | PD fellow, 4 fte | Research coordinator in Tanzania, 4fte | PM * | PM | PM | PM | PM |
| 2 | Personnel | PhD fellow (Dutch), 4 fte | PhD (Clinical medicine) | PM | PM | PM | PM | PM |
| 3 | Personnel | PhD fellows (Tanzanian) | Allowances for study in The Netherlands ^1^ | 26,400 | 0 | 0 | 26,400 | **52,800** |
| 4 | Personnel | PhD fellows (Tanzanian) | Allowances for study in Tanzania ^2^ | 0 | 7,200 | 7,200 | 0 | **14,400** |
| 5 | Personnel | Bench fees | 4 Fellows × € 4,538 | 0 | 18,152 | 0 | 0 | **18,152** |
| 6 | Personnel | Work and study permits | Work permits for Research Fellow and Dutch PhD fellow | 500 | 500 | 500 | 0 | **1,500** |
| 7 | Personnel | Clinical officer/Assist Med Officer | 24 Months × € 250 | 0 | 3,000 | 3,000 | 0 | **6,000** |
| 8 | Personnel | Laboratory assistants | 33 Months × € 150 (immunology, parasitology) | 450 | 2,250 | 2,250 | 0 | **4,950** |
| 9 | Personnel | Field assistants | 36 Months × € 150 | 1,800 | 1,800 | 1,800 | 0 | **5,400** |
| 10 | Personnel | Enumerations CHWs | (CHW: community health worker) | 4,000 | 5,500 | 12,400 | 0 | **21,900** |
| 11 | Travel | Travel in The Netherlands | Meetings between post-doc, PhD fellows, supervisors, etc. ^3^ | 1,000 | 500 | 500 | 1,000 | **3,000** |
| 12 | Travel | Travel Netherlands-Tanzania | Return flights, visa, etc. (6 flights × € 750/flight) ^4^ | 1,500 | 1,500 | 1,500 | 0 | **4,500** |
| 13 | Travel | Travel in Tanzania | Including costs of transport, accommodation, etc. | 1,000 | 1,000 | 1,000 | 0 | **3,000** |
| 14 | Durables | Vehicles | 2 Vehicles × € 25,000 (including spare parts, maintenance) | 50,000 | 0 | 0 | 0 | **50,000** |
| 15 | Durables | Motorbikes | 2 Motorbikes × € 4,000 | 8,000 | 0 | 0 | 0 | **8,000** |
| 16 | Durables | Small office equipment |  | 500 | 500 | 0 | 0 | **1,000** |
| 17 | Durables | Clinical equipment | Haemoglobin meters, weighing scales, kneemometer, etc. | 1,000 | 1,000 | 0 | 0 | **2,000** |
| 18 | Durables | Electrophysiological equipment | EEC, etc. | 500 | 500 | 0 | 0 | **1,000** |
| 19 | Durables | Notebook computers, printer | 4 Computers × € 1,500 | 6,000 | 0 | 0 | 0 | **6,000** |
| 20 | Durables | Computer peripherals | 1 Printer € 1,000; 3 UPS × € 200, miscell. Equipment | 1,500 | 500 | 0 | 0 | **2,000** |
| 21 | Durables | Liquid nitrogen containers | Including peripherals | 3,000 | 3,000 | 0 | 0 | **6,000** |
| 22 | Consumables | Vehicle use, fixed costs | Car and motorbike insurance, road tax, anti-theft devices | 750 | 750 | 750 | 0 | **2,250** |
| 23 | Consumables | Vehicle use, variable costs | 125,000 km × € 0.09 | 3,750 | 3,750 | 3,750 | 0 | **11,250** |
| 24 | Consumables | Field facilities | Rent research clinic and laboratory | 1,000 | 1,000 | 1,000 | 0 | **3,000** |
| 25 | Consumables | Experimental supplements | 672 kids × 182 d × € 0.20/d (multinutrients) | 2,500 | 21,960 | 0 | 0 | **24,460** |
| 26 | Consumables | Clinical supplies | Drugs, needles, syringes, diagnostic supplies, etc. | 1,000 | 2,000 | 2,000 | 0 | **5,000** |
| 27 | Consumables | Immunology consumables | Ficoll, labelled antibodies for flow cytometry, CBA kits, etc. | 9,000 | 18,000 | 18,000 | 0 | **45,000** |
| 28 | Consumables | Biochemical/laboratory analyses | Details can be provided upon request | 46,540 | 0 | 35,850 | 114,820 | **197,210** |
| 29 | Consumables | Renumeration parents | Time/travel compensation, psychological tests children | 1,000 | 1,000 | 2,750 | 0 | **4,750** |
| 30 | Consumables | Library support, books, software |  | 1,250 | 1,250 | 750 | 750 | **4,000** |
| 31 | Consumables | Office supplies |  | 1,000 | 1,000 | 1,000 | 1,000 | **4,000** |
| 32 | Consumables | Communications | Including courier services, e-mail, web services, telephone | 500 | 500 | 500 | 500 | **2,000** |
| 33 | Consumables | Freight | Exper. supplements, plasma samples, consumables etc. | 1,000 | 2,000 | 2,000 | 0 | **5,000** |
| 34 | Consumables | Workshops, meetings, etc. | Within and between The Netherlands and Tanzania | 1,000 | 500 | 500 | 1,000 | **3,000** |
| 35 | Consumables | Costs of publications |  | 500 | 2,000 | 2,000 | 2,000 | **6,500** |
| 36 | Miscellaneous | Laboratory costs | Laboratory facilities and assistance in The Netherlands | 12,500 | 12,500 | 12,500 | 12,500 | **50,000** |
| 37 | Miscellaneous | Joint workshop |  | 7,500 | 0 | 0 | 4,000 | **11,500** |
| 38 | Miscellaneous | Unforeseen | Including research clearances (KCMC, NIMR, COSTECH) | 5,686 | 6,000 | 6,657 | 5,843 | **24,186** |
|  | **Total** |  |  | **203,626** | **121,112** | **120,157** | **169,813** | **614,708** |

* PM: pro memori

^1^ 2 persons × 24 months × € 1,100/month = € 52,800; ^2^ 2 persons × 24 months × € 300/month = € 14,400; ^3^ Including costs of 16 short visits (travel and accommodation @ € 125/visit) of PhD Fellow (psychology) to Tilburg University during the study period in The Netherlands (as foreseen, he/she will be based in Wageningen in this period), and costs within The Netherlands of 2 visits of 1 month each (travel and accommodation @ € 500/visit) by postdoctoral student during the field work period to coordinate with all supervisors in The Netherlands, to visit specialized libraries, etc.; ^4^ Including costs of 3 return visits (bus, train, airfare, visa, malarial prophylaxis, etc.) by Postdoctoral Fellow and 2 PhD Fellows between The Netherlands and Tanzania and, similarly, costs of 3 return visits by supervisors between The Netherlands and Tanzania.

Annex 5. **Ethical issues relating to blood collection in study children**

We will repeatedly collect venous blood samples from each child participating in our study. This may raise ethical questions about the discomfort caused to the child, the method of blood collection, and the probability and extent that the frequency and volumes of blood collected may cause adverse effects such as anaemia. During each phlebotomy, we will collect one tube (maximally 5.9 mL) of venous blood because this volume is minimally required to conduct large numbers of tests as foreseen in this study. Additionally, this blood volume is needed to achieve the correct blood anticoagulent concentration in the special tubes for trace element analysis used in our trial. Lastly, it is technically not possible to collect smaller blood volumes for our study (e.g. 5 mL): with the collection system used, blood is drawn into the container by filling and replacing a vacuum. Thus the flow of blood stops automatically when the vacuum in the tube has dissipated. This maximally yields 5.9 mL; for many of the children studied, the blood volume collected was even less because the vacuum in the tubes is gone earlier. Thus in our pilot study, the volume actually collected was maximally 5.9 mL, and often less because the vacuum in the tubes had disappeared earlier. *It should be noted that there are no collection tubes commercially available that are both suitable for trace element analysis and yield smaller blood volumes.*

To minimize discomfort to children, we will use a butterfly collection device, with paediatric needles (23G). Compared to conventional needles, butterfly needles are thought to increase the success rate of venipuncture and to decrease patients’ discomfort (Hefler et al. 2004). There appears to be no evidence that blood collection with the butterfly device is associated with decreased quantities of blood being collected; however, there is anecdotic evidence that this is associated with an increased rate of haemolysis compared with the use of conventional needles (Miller et al. 2004). It should also be noted that venipuncture is probably less painful than heel punctures in infants undergoing routine blood sampling (Shah et al. 1997).

In the intervention study, we will maximally collect three blood samples by venipuncture, namely at baseline, at the first episode of slide-confirmed malaria and at the end of the intervention. However, a volume of 5.9 mL corresponds to <1% of the total blood volume of children at aged >6 months (figure 1). At normal rates of erythropoiesis, approximately 1% of the total red cells are replaced. In case of blood loss, this production rate can easily be doubled. Hence, it is highly unlikely that our procedures for the collection of venous blood would lead or contribute to anaemia in any of the children studied.

Figure 1. **Relationship of a blood sample volumes to total blood volume in children, by age**

Blood sample volumes of 5 mL, 7.5 mL and 10 mL correspond to lines marked with solid circles, open squares and solid triangles, respectively. Calculations were based on the following blood volumes per kg body weight: prematures, 115 mL/kg; newborns, 80-110 mL/kg; infants and children, 75-100 mL/kg. Adapted from: Microtechniques for the clinical laboratory: concepts and applications. New York, NY: John Wiley & Sons, Inc., 1976 (quoted by NCCLS, 1999).

Annex 6. **Selected references from the research group**

1. Akim NI, Drakely CJ, Kingo T, Simon B, Senkoro K, Sauerwein RW. Dynamics of P. falciparum gametocytemia in symptomatic patients in an area of intense perennial transmission in Tanzania. Am J Trop Med Hyg 2000;63:199-203. [2.1]
2. Akim NI, Usarra H, Drakely CJ, Sauerwein RW, Kitua AY. Immunity to the sexual stages of Plasmodium falciparum in mothers, neonates and infants subject to intense and perennial malarial transmission. Ann Trop Med Parasitol 2002;96:735-37. [1.0]
3. Barrat FJ, Cua DJ, Boonstra A, Richards DF, Crain C, Savelkoul HFJ, et al. In vitro generation of interleukin-10 producing regulatory CD4+ T cells is induced by immunosuppressive drugs and inhibited by T helper 1 (Th1)- and Th2-inducing cytokines. J Exp Medicine 2002;195:603-16. [15.3]
4. Bleichrodt N, Shrestha RM, West CE, Hautvast JG, Van de Vijver FJ, Born MP. The benefits of adequate iodine intake. Nutr Rev 1996;54:S72-78. [2.5]
5. Boonstra AP, Barrat FJ, Crain C, Heath VL, Savelkoul HFJ, O’Garra A. 1α,25-dihydroxyvitamin D3 has a direct effect on naïve CD4+ T cells to enhance the development of Th2 cells. J Immun 2001;167:4974-80. [7.1]
6. Elton M, Winter O, Heslenfeld DJ, et al. Event-related potentials to tones in the presence and absence of sleep spindles. J Sleep Res 1997;6:78-83. [2.6]
7. Louwman MW, Van Dusseldorp M, Van de Vijver FJ, et al. Signs of impaired cognitive function in adolescents with marginal cobalamin status. Am J Clin Nutr 2000;72:762-69. [5.0]
8. Umeta M, West CE, Verhoef H, Haidar J, Hautvast JG. Long-term effects of zinc supplementation on growth of children in rural Ethiopia: a randomised controlled trial (submitted).
9. Umeta M, West CE, Verhoef H, Haidar J, Hautvast JG. Factors associated with stunting in infants aged 5-11 months in the Dodota-Sire district, rural Ethopia. J Nutr 2003;133:1064-69. [3.2]
10. Van den Briel T, West CE, Bleichrodt N, Van de Vijver FJ, Ategbo EA, Hautvast JG. Improved iodine status is associated with improved mental performance of schoolchildren in Benin. Am J Clin Nutr 2000;72:1179-85. [5.0]
11. Van de Vijver FJ, Jongmans MJ. Learning potential from a cross-cultural perspective: problems and perspectives. In: Learning potential assessment and cognitive training: actual research and perspectives in theory building and methodology, Volume 7. (GM Van der Aalsvoort, W Resing, AJ Ruijssenaars, eds.). Oxford, UK: Elsevier, 2002: p. 211-26.
12. Van de Vijver AJR, Willemse GR. Are reaction time tasks better suited for cultural minorities than paper pencil tests? In: Contemorary issues in cross-cultural psychology (Bleichrodt N, Drenth PJ, eds.). Lisse, The Netherlands: Swets and Zeitlinger, 1991: 450-64.
13. Verhoef H, West CE. Validity of the relative dose-response test and the modified relative dose-response test as indicators of vitamin A stores in liver. Am J Clin Nutr 2005;81:835-39. [4.6]
14. Verhoef H, West CE, Bleichrodt N, Dekker PH, Born MP. Effects of micronutrients during pregnancy and early infancy on mental and psychomotor development. In: Micronutrient deficiencies in the first months of life (Delange F, West KP, eds.). Nestlé Nutrition Workshop Series Pediatric Program, Vol 52. Vevey, Switzerland: Nestec, Ltd., 2003:327-357.
15. Verhoef H, West CE, Kraaijenhagen R, et al. Malarial anemia leads to adequately increased erythropoiesis in asymptomatic Kenyan children. Blood 2002;100:3489-94. [9.3]
16. Verhoef H, West CE, Ndeto P, Burema J, Beguin Y, Kok FJ. Serum transferrin receptor concentration indicates increased erythropoiesis in Kenyan children with asymptomatic malaria. Am J Clin Nutr 2001;74:767-75. [4.6]
17. Verhoef H, West CE, Nzyuko SM, de Vogel S, van der Valk R, Wanga MA, Kuijsten A, Veenemans J, Kok FJ. Intermittent administration of iron and sulfadoxine-pyrimethamine to control anaemia in Kenyan children: a randomised controlled trial. Lancet 2002;360:908-14. [13.3]
18. Waters AP, Sauerwein RW, Arnot D. Malaria vaccine research – setting the record straight. Nat Med 2000;6:234. [28.7]

Annex 7. **Curricula vitarum**

# Curriculum vitae Huub Savelkoul

**1. Personal data**

*Name*: Hubertus Fransciscus Jozef Savelkoul

*Date of birth*: July 11, 1956

*Work address*: Cell biology and Immunology, Wageningen University, P.O. Box 338, 6700 AH Wageningen, The Netherlands; tel: +31.317.483509

E-mail: [huub.savelkoul@wur.nl](mailto:huub.savelkoul@wur.nl)

**2. Scientific Career**

*MSc*

Biology, specialisations Zoology and Cell Biology; Wageningen University (1974-1981)

Majors: Biochemistry, Cell Biology, Genetics.

Foreign experience: Department of Chemical Immunology, Weizmann Institute of Science, Rehovot, Israel (1981). Advisor: prof.dr. P. Lonai

*PhD*

Erasmus University Rotterdam (September 2, 1988, *cum laude*)

Thesis: “Induction and measurement of IgE. A study in mice with emphasis on the regulatory role of lymphokines”. Promotor: prof.dr. R. Benner

*Research price* Erasmus University Rotterdam (1989)

*Postdoc*: DNAX Research Institute of Meolecular and Cellular Biology, Palo Alto, California, USA (1988-1990). Advisor: dr. R.L. Coffman

# *Courses:*

- Radiation safety: Stralingsbescherming C-niveau: J.A. Cohen Interuniversitair Instituut voor Radiopathologie en Stralenbescherming (IRS), Leiden) (1984)
- Biostatistics: Post-Academisch Onderwijs Geneeskunde Rotterdam (1986)
- Teaching: Didactische Vaardigheden, Bureau Smidts-Wijnen, Rotterdam (1996)

*Registration as* Immunologist (SMBWO, 1991)

# *Lecturer*

Assistent Professor (1990-1996) and Associate Professor (1996-2000):

Erasmus University Rotterdam, department of Immunology (chairman: prof.dr. R. Benner)

Unit: Immunoregulation and the development of allergy at childhood age

# *Professor*

Van der Leeuw chair (NWO) in Cell Biology and Immunology, Wageningen University (September 2000-September 2003). As of September 1, 2003 full professor and head of department Cell Biology and Immunology.

**3. Research**

*PhD Promotions*:

Co-promotor: R. Van Ommen (1994), A.C.T.M. Vossen (1995), S.S. Pathak (1995), T. Ten Hagen (1996), H. Koning (1996), M.P. Laan (1999).

Promotor: A. Boonstra (2000), M. Nawijn (2001), C. Kruiswijk (2002), H. Huttenhuis (2005), M.O. Huising (2006), M. Joerink (2006), J. Arts (2006)

*References* (1982-2005)

Research papers in peer-reviewed journals: 255; citations: 2350

Others: book chapters, reviews, etc: 80

*Approved Research projects*: 39 (1994-2005).

*Patents:* 8

# *Current group:*

# 4 senior staff, 4 postdoctoral fellows, 12 graduate students, 20 undergraduate students, 6 technical support staff, 1 secretary, 1 part-time professor (Professor dr H.J. Wichers).

# 4. Memberships

Editorial board member: Mediators of Inflammation, Molecular Immunology, Science and Technology Intensive Courses

Member Scientific advisory board Netherlands Asthma Foundation (1996-2000)

Netherlands Society for Allergology, Netherlands Society for Immunology, European Academy of Allergology and Clinical Immunology, European Society of Paediatric Allergology and Clinical Immunology

Chairman: Educational Committee Netherlands Society for Immunology (1999-2005)

Chairman: Users group Animal Experiments Wageningen University (2003-current)

Board Member: Netherlands Medical-biological research council (SMBWO 1996-current)

Board Member: Stichting Astma Bestrijding (1999-current)

Member Scientific advisory board: Adviescommissie Landelijk Overleg Onderwijs-Arbeid, HBO-opleiding Biologie en Medisch Laboratoriumonderzoek, HBO-raad (1999-2005)

Chairman Curriculum board Biology, Wageningen University (2002-2006)

**5. Specialisations**

Basic and applied immunology, immunoregulation, immunoassays, regulatory T cells, cytokines, fish immunology, allergy

**Publications (grouped by year)**

**2006**

Bosma, R.H.; Savelkoul, H.F.J.; Frankena, K.; Baars, T.; Laarakker, E. (2006) [Dairy herd health, impedance on six acupuncture points and immune response factors in milk: A pilot study](http://dx.doi.org/10.1016/j.livprodsci.2005.07.002). *Livestock Science 99 (2006)2-3. - ISSN 1871-1413 - p. 285 - 290.*

Gilissen, L.J.W.J.; Wichers, H.J.; Savelkoul, H.F.J.; Bogers, R.J. (2006) [Allergy matters: new approaches to allergy prevention and management: the international conference on allergy prevention, Wageningen, The Netherlands 4-6 February 2004](http://library.wur.nl/frontis/allergy_matters/index.html). *Berlin [etc.] : Springer, 2006 (Wageningen UR Frontis Series 10) - ISBN 1-4020-3895-X - p. 205.*

Joerink, M.; Savelkoul, H.F.J.; Wiegertjes, G.F. (2006) [Evolutionary conservation of alternative activation of macrophages: structural and functional characterization of arginase 1 and 2 in carp (Cyprinus carpio L.)](http://dx.doi.org/10.1016/j.molimm.2005.07.022). *Molecular Immunology 43 (2006)8. - ISSN 0161-5890 - p. 1116 - 1128.*

Kachamakova, N.M.; Irnazarow, I.; Parmentier, H.K.; Savelkoul, H.F.J.; Pilarczyk, A.; Wiegertjes, G.F. (2006) [Genetic differences in natural antibody levels in common carp (Cyprinus carpio L.)](http://dx.doi.org/10.1016/j.fsi.2006.01.005). *Fish and Shellfish Immunology 21 (2006)4. - ISSN 1050-4648 - p. 404 - 413.*

**2005**

Gilissen, L.J.W.J.; Wichers, H.J.; Savelkoul, H.F.J.; Beers, G. (2005) [Future developments in allergy prevention : a matter of integrating medical, natural and social sciences](http://library.wur.nl/frontis/allergy_matters/01_gilissen.pdf). *In: Allergy Matters : new approaches to allergy prevention and management / Gilissen, L.J.E.J. Dr., Wichers, H.J. Prof.dr., Savelkoul, H.F.J. Prof.dr.ir., Bogers, J.R., . - Springer - Life Sciences, 2005 (Wageningen UR Frontis Series 10) - ISBN 1-4020-3896-8 - p. 3 - 10.*

Heselmans, M.; Reid, G.; Akkermans, L.M.A.; Savelkoul, H.F.J.; Timmerman, H.; Rombouts, F.M. (2005) Gut flora in health and disease : potential role of probiotics. *Current Issues in Intestinal Microbiology 6 (2005)1. p. 1 - 8.*

Huising, M.O.; Kruiswijk, C.P.; Schijndel, J. van; Savelkoul, H.F.J.; Flik, G.; Verburg-van Kemenade, B.M.L. (2005) [Multiple and highly divergent IL-11 genes in teleost fish](http://dx.doi.org/10.1007/s00251-005-0012-2). *Immunogenetics 57 (2005)6. - ISSN 0093-7711 - p. 432 - 443.*

Jeurink, P.V.; Savelkoul, H.F.J. (2005) Kruisreacties bij voedselallergie. *NVOX Tijdschrift voor Natuurwetenschap op School 30 (2005)1. p. 3 - 5.*

Jeurink, P.V.; Savelkoul, H.F.J. (2005) [Induction and regulation of allergen-specific IgE](http://library.wur.nl/frontis/allergy_matters/02_savelkoul.pdf). *In: Allergy Matters : new approaches to allergy prevention and management / Gilissen, L.J.E.J. Dr., Wichers, H.J. Prof.dr., Savelkoul, H.F.J. Prof.dr.ir., Bogers, R.J., . - Springer - Life Sciences, 2005 (Wageningen UR Frontis Series 10) p. 13 - 27.*

Joerink, M.; Ribeiro, S.; Savelkoul, H.F.J.; Wiegertjes, G.F. (2005) Alternative activation of fish macrophages: structural and functional characerization of arginase 1 and 2 in carp (Cyprinus carpio L.). *In: Abstracts 12th meeting of the European association of fish pathologists, Copenhagen 11-16 September 2005. - Copenhagen, Denmark : 2005*

Joerink, M.; Savelkoul, H.F.J.; Wiegertjes, G.F. (2005) [Carp (Cyprinus carpio L.) macrophage polarization in resonse to parasites](http://library.wur.nl/wasp/bestanden/LUWPUBRD_00342783_A502_001.pdf)
*In: Abstracts of the 19th meeting of the European Macrophage and Dendritic Cell Society, Amsterdam, 6-8 October 2005. Amsterdam : 2005*

Jurecka, P.M.; Rakus, K.L.; Onara, D.F.; Kachamakova, N.; Forlenza, M.; Wiegertjes, G.F.; Savelkoul, H.F.J.; Pilarczyk, A.; Irnazarow, I. (2005) Genetic differences in disease resistance : results of challenges with Trypanoplasma borreli in six common carp (Cyprinus carpio L.) lines. *In: Abstracts 12th meeting of the European association of fish pathologists, Copenhagen 11-16 September 2005. - Copenhagen, Denmark : 2005*

Kachamakova, N.; Wiegertjes, G.F.; Savelkoul, H.F.J.; Pilarczyk, A.; Irnazarow, I. (2005) Genetically determined differences in levels of natural antibodies in carp (Cyprinus carpio L.). *In: Abstracts 12th meeting of the European association of fish pathologists, Copenhagen, 11-16 September 2005. - Copenhagen, Denmark: 2005*

Kruiswijk, C.P.; Hermsen, G.J.; Heerwaarden, J. van; Dixon, B.; Savelkoul, H.F.J.; Stet, R.J.M. (2005) [Major histocompatibility genes in the Lake Tana African large barb species flock: evidence for complete partitioning of class II B, but nog class I, genes among different species](http://dx.doi.org/10.1007/s00251-005-0767-5). *Immunogenetics 56 (2005)12. p. 894 - 908.*

Lull Noguera, C.; Wichers, H.J.; Savelkoul, H.F.J. (2005) [Antiinflammatory and immunomodulating properties of fungal metabolites](http://dx.doi.org/10.1155/MI.2005.63)
*Mediators of Inflammation 2005 (2005)2. p. 63 - 80.*

Savelkoul, H.F.J.; Cameron, S.B.; Chow, A.W. (2005) Maturation of the immune response. *In: Encyclopedic Reference of Immunotoxicology / Werner, H.W., . - Heidelberg : Springer, 2005.*

Savelkoul, H.F.J. (2005) Allergie : ook voor allergie geldt: voorkomen is beter dan genezen. *Patient Care 32 (2005)10. p. 56 - 58.*

Savelkoul, H.F.J. (2005) De darm, zijn flora en het brein. *Folia Orthica 2005 (2005)2. - p. 6 - 9.*

Savelkoul, H.F.J. (2005) Allergie : een probleem voor mens en dier. *Animal Sciences Krant (2005)2005-10-01. - p. 2.*

Stet, R.J.M.; Hermsen, G.J.; Westphal, A.H.; Jukes, J.; Engelsma, M.Y.; Verburg-van Kemenade, B.M.L.; Dortmans, J.C.F.M.; Aveiro, J.; Savelkoul, H.F.J. (2005) [Novel immunoglobulin-like transcripts in teleost fish encode polymorphic receptors with cytoplasmic ITAM or ITIM an a new structural Ig domain similar to the natural cytotoxicity receptor NKp44](http://dx.doi.org/10.1007/s00251-005-0771-9). *Immunogenetics 57 (2005)1-2. p. 77 - 89.*

Sun, N.; Yang, G.; Zhao, H.; Savelkoul, H.F.J.; An, L. (2005) [Multidose streptozotocin induction of diabetes in BALB/c mice induces a dominant oxidative macrophage and a conversion of TH1 to TH2 phenotypes during disease progression](http://dx.doi.org/10.1155/MI.2005.202).. *Mediators of Inflammation 2005 (2005)4. p. 202 - 209.*

**2004**

Huising, M.O.; Stet, R.J.M.; Savelkoul, H.F.J.; Kemenade, B.M.L. van (2004) [The molecular evolution of the interleukin-1 family of cytokines; IL-18 in teleost fish](http://dx.doi.org/10.1016/j.dci.2003.09.005). *Developmental and Comparative Immunology 28 (2004). p. 395 - 413.*

Jeurink, P.V.; Savelkoul, H.F.J. (2004) [Kruisreacties bij voedselallergie](http://www.mngm.nl/voedsel_map/kruisreacties.pdf). *Voeding Nu 6 (2004). p. 23 - 26.*

Kruiswijk, C.P.; Hermsen, G.J.; Fujiki, K.; Dixon, B.; Savelkoul, H.F.J.; Stet, R.J.M. (2004) [Analysis of genomic and expressed major histocompatibility class Ia and class II genes in a hexaploid Lake Tana African 'large' barb individual (Barbus intermedius)](http://dx.doi.org/10.1007/s00251-003-0635-0). *Immunogenetics 55 (2004). p. 770 - 781.*

Lammers, A.; Klomp, M.E.V.; Nieuwland, M.G.B.; Savelkoul, H.F.J.; Parmentier, H.K. (2004) [Adoptive transfer of natural antibodies to non-immunized chickens affects subsequent antigen-specific humoral and cellular immune responses](http://dx.doi.org/10.1016/S0145-305X(03)00102-2). *Developmental and Comparative Immunology 28 (2004). p. 51 - 60.*

Parmentier, H.K.; Lammers, A.; Hoekman, J.J.; Vries Reilingh, G. de; Zaanen, I.T.A.; Savelkoul, H.F.J. (2004) [Different levels of natural antibodies in chickens divergently selected for specific antibody responses](http://dx.doi.org/10.1016/S0145-305X(03)00087-9). *Developmental and Comparative Immunology 28 (2004). p. 39 - 49.*

Parmentier, H.K.; Kieboom, W.J.A. van den; Nieuwland, M.G.B.; Vries Reilingh, G. de; Hangalapura, B.N.; Savelkoul, H.F.J.; Lammers, A. (2004) [Differential effects of lipopolysaccharide and lipoteichoic acid on the primary antibody response to keyhole limpet hemocyanin of chickens selected for high or low antibody responses to sheep red blood cells](http://www.poultryscience.org/ps/papers/04/p0471133.pdf). *Poultry Science 83 (2004). p. 1133 - 1139.*

Parmentier, H.K.; Baelmans, R.; Savelkoul, H.F.J.; Dorny, P.; Demey, F.; Berkvens, D. (2004) [Serum haemolytic complement activities in 11 different MHC (B) typed chicken lines](http://dx.doi.org/10.1016/j.vetimm.2004.02.009). *Veterinary Immunology and Immunopathology 100 (2004). p. 25 - 32.*

Savelkoul, H.F.J. (2004) [Hatsjie!](http://library.wur.nl/wasp/bestanden/LUWPUBRD_00335236_A502_001.pdf) *Intermediair 26 (2004). p. 41 - 43.*

Savelkoul, H.F.J. (2004) De darmflora van een autist. *Limburgs Dagblad (2004)2004-07-21.*

Savelkoul, H.F.J. (2004) [Autisme is meer dan een hersenziekte](http://www.allergie.wur.nl/div/041020InterviewAutisme.pdf). *Weleda Artsen Forum (2004). - p. 1 - 3.*

Savelkoul, H.F.J. (2004) Preventie van een allergie: een gezondheidsissue. *In: Syllabus symposium - Nutricia, Duik in de wereld van allergiepreventie, recente voedingskundige inzichten en toekomstige ontwikkelingen, Harderwijk 14-10-2004. - Syllabus symposium - Nutricia Nederland N.V. (2004). - p. 4 - 8.Harderwijk : 2004 - p. 4 - 8.*

Savelkoul, H.F.J.; Gilissen, L.J.W.J. (2004) [Allergie: overgevoeligheid voor voedsel genetisch aangepakt](http://library.wur.nl/wasp/bestanden/LUWPUBRD_00335245_A502_001.pdf). *Resource: magazine van Wageningen Universiteit & Researchcentrum (2004). p. 12 - 14.*

Savelkoul, H.F.J. (2004) [Voedselallergie, relatie tussen klachten en voedsel alleen aantoonbaar via provocatietest](http://library.wur.nl/wasp/bestanden/LUWPUBRD_00335291_A502_001.pdf). *Medisch Contact 59 (2004). p. 2080 - 2083.*

Savelkoul, H.F.J. (2004) Strijd tegen allergie. *Algemeen Dagblad (2004)2004-02-03. - p. 1.*

Stolte, H.H.; Leon, K.M.; Savelkoul, H.F.J.; Flik, G.; Kemenade, B.M.L. van (2004) [Stress regulated corticosteroid receptor expression in common carp (Cyprinus carpio L.)](http://www.5isfe.uji.es/cgi-bin/submitted/mostrarAbstract.php?id=178). *In: Abstracts 5th International Symposium on Fish Endocrinology, Castellon, Spanje, 5-9 September 2004. - Castellon, Spanje : 2004 - p. 164.*

Stolte, H.H.; Leon, K.M.; Kramer, J.; Flik, G.; Savelkoul, H.F.J.; Kemenade, B.M.L. van (2004) Identification of immuno-regulatory molecules of fish by micro-array technology. *In: Abstracts of PhD retreat/WIAS symposium May 2004. - Nijmegen : 2004 - p. 41.*

Weijden, W.J. van der; Savelkoul, H.F.J.; Hin, K.J.; Wilt, J. de (2004) [Dierziektebeleid spot met Darwin](http://www.clm.nl/publicaties/data/dierziekten2.pdf). *Tijdschrift voor Diergeneeskunde 129 (2004). - ISSN 0040-7453 - p. 418 - 420.*

Wichers, H.J.; Beijer, T. de; Savelkoul, H.F.J.; Amerongen, A. van (2004) [The major peanut allergen Ara h 1 and its cleaved-off N-terminal peptide; possible implications for peanut allergen detection](http://dx.doi.org/10.1021/jf049697o). *Journal of Agricultural and Food Chemistry 52 (2004). p. 4903 - 4907.*

**2003**

Cameron, S.B.; Stolte, H.H.; Chow, A.W.; Savelkoul, H.F.J. (2003) [T helper cell polarisation as a measure of the maturation of the immune response](http://dx.doi.org/10.1080/0929350310001619744). *Mediators of Inflammation 12 (2003). p. 285 - 292.*

Dickson, B.C.; Yang, H.; Savelkoul, H.F.J.; Rowden, G.; Rooijen, N. van; Wright Jr., J.R. (2003) [Islet transplantation in the discordant tilapia-to-mouse model: a novel application of alginate microencapsulation in the study of xenograft rejection](http://dx.doi.org/10.1097/01.TP.0000048226.28357.0D). *Transplantation 75 (2003). p. 599 - 606.*

Eek, A.; Savelkoul, H.F.J. (2003) [Allergie en de hygienehypothese: de rol van een verstoord regulatoir netwerk](http://library.wur.nl/wasp/bestanden/LUWPUBRD_00325526_A502_001.pdf). *Foliolum 3 (2003). - p. 7 - 13.*

Forlenza, M.; Saeij, J.P.J.; Zou, J.; Secombes, C.J.; Savelkoul, H.F.J.; Stet, R.J.M.; Wiegertjes, G.F. (2003) The role of nitric oxide in the immune response to 'tryps' in carp, Cyprinus carpio. *In: 9th International Congress of ISDCI, St. Andrews Scotland; programme & abstracts. –*

Forlenza, M.; Scharsack, J.P.; Savelkoul, H.F.J.; Wiegertjes, G.F. (2003) Functional characterisation of carp leukocytes by flow cytometry and the corresponding expression profile of their immune regulatory genes. *In: 11th International Conference of the EAFP, St. Julians Malta. –*

Guo, F.; Savelkoul, H.F.J.; Kwakkel, R.P.; Williams, B.A.; Verstegen, M.W.A. (2003) [Immunoactive, medicinal properties of mushroom and herb polysaccharides and their potential use in chicken diets](http://library.wur.nl/wasp/bestanden/LUWPUBRD_00327493_A502_001.pdf). *Worlds Poultry Science Journal 59 (2003). p. 427 - 440.*

Huising, M.O.; Stet, R.J.M.; Kruiswijk, C.P.; Savelkoul, H.F.J.; Kemenade, B.M.L. van (2003) [Molecular evolution of CXC chemokines: extant CXC chemokines originate from the CNS](http://dx.doi.org/10.1016/S1471-4906(03)00120-0). *Trends in Immunology 24 (2003). p. 306 - 312.*

Huising, M.O.; Stet, R.J.M.; Kruiswijk, C.P.; Savelkoul, H.F.J.; Kemenade, B.M.L. van (2003) [Response to shields: molecular evolution of CXC chemokines and receptors](http://dx.doi.org/10.1016/S1471-4906(03)00165-0). *Trends in Immunology 24 (2003). p. 356 - 357.*

Huising, M.O.; Guichelaar, T.; Hoek, C.; Kemenade, B.M.L. van; Flik, G.; Savelkoul, H.F.J.; Rombout, J.H.W.M. (2003) [Increased efficacy of immersion vaccination in fish with hyperosmotic pretreatment](http://dx.doi.org/10.1016/S0264-410X(03)00497-3). *Vaccine 21 (2003). p. 4178 - 4193.*

Huising, M.O.; Stolte, H.H.; Flik, G.; Savelkoul, H.F.J.; Kemenade, B.M.L. van (2003) [CXC chemokines and leukocyte chemotaxis in common carp (Cyprinus carpio L.)](http://dx.doi.org/10.1016/S0145-305X(03)00082-X). *Developmental and Comparative Immunology 27 (2003). p. 875 - 888.*

Huising, M.O.; Kruiswijk, C.P.; Stet, R.J.M.; Savelkoul, H.F.J.; Kemenade, B.M.L. van (2003) The ancestral role of CXC chemokines: CXC chemokines predate the immune system. *In: Sixth European Winter Conference in Immunity. –*

Huising, M.O.; Stolte, H.H.; Flik, G.; Savelkoul, H.F.J.; Kemenade, B.M.L. van (2003) Evolution and function of CXC chemokines and their receptors in vertebrates. *In: 9th International Congress of ISDCI St Andrews, Scotland; Programme & Abstracts. –*

Huising, M.O.; Schijndel, J. van; Kruiswijk, C.P.; Joerink, M.; Kemenade, B.M.L. van; Savelkoul, H.F.J. (2003) Evolution of vertebrate cytokines. *In: Wintermeeting Dutch Society for Immunology. –*

Joerink, M.; Savelkoul, H.F.J.; Wiegertjes, G.F. (2003) Macrophage polarisation in carp (Cyprinus carpio). *In: 9th International Congress of ISDCI, St. Andrews Scotland; programme & abstracts. –*

Joerink, M.; Savelkoul, H.F.J.; Wiegertjes, G.F. (2003) Classically versus alternatively activated macrophages in fish. *In: Wintermeeting Dutch Society for Immunology. –*

Kemenade, B.M.L. van; Huising, M.O.; Savelkoul, H.F.J. (2003) [Molecular evolution of the interleukin-1 family of cytokines: fish have ILI-18 and IL-18 receptors](http://library.wur.nl/wasp/bestanden/LUWPUBRD_00325456_A502_001.pdf). *In: 9th International Congress of ISDCI St Andrews, Scotland; programme & abstracts. –*

Koopman, L.P.; Savelkoul, H.F.J.; Benten, I.J. van; Gerritsen, J.; Brunekreef, B.; Neijens, H.J. (2003) [Increased serum IL-10/IL-12 ratio in wheezing infants](http://dx.doi.org/10.1034/j.1399-3038.2003.00019.x). *Pediatric Allergy and Immunology 14 (2003). p. 112 - 119.*

Kruiswijk, C.P.; Hermsen, G.J.; Heerwaarden, J. van; Dixon, B.; Savelkoul, H.F.J.; Stet, R.J.M. (2003) Different modes of major histocompatibility class I and class II evolution in the Lake Tana Barbus species flock. *In: 9th International Congress of ISDCI, St. Andrews Scotland; programme & abstracts. –*

Lammers, A.; Savelkoul, H.F.J.; Parmentier, H.K. (2003) Identification and immuno-modulatory activity of chicken natural antibodies. *In: Abstracts of the 15th European Immunology Congress (EFIS 2003). - Immunology Letters 87 (2003). p. 40.*

Linde, K. van der; Boor, P.P.C.; Sandkuijl, L.A.; Meijssen, M.A.C.; Savelkoul, H.F.J.; Wilson, J.H.P.; Rooij, F.W.M. (2003) [A Gly15Arg mutation in the Interleukin-10 gene reduces secretion of Interleukin-10 in Crohn disease](http://dx.doi.org/10.1080/00365520310003011). *Scandinavian Journal of Gastroenterology 38 (2003). p. 611 - 617.*

Rakus, K.L.; Wiegertjes, G.F.; Stet, R.J.M.; Savelkoul, H.F.J.; Pilarczyk, A.; Irnazarow, I. (2003) [Polymorphism of major histocompatibility complex class II B genes in different carp lines of the common carp (Cyprinus carpio L.)](http://dx.doi.org/10.1016/S0990-7440(03)00057-3). *Aquatic Living Resources 16 (2003). p. 432 - 437.*

Rakus, K.L.; Stet, R.J.M.; Irnazarow, I.; Pilarczyk, A.; Savelkoul, H.F.J.; Wiegertjes, G.F. (2003) Allelic variation of MHC class II B in genetically different lines of common carp. *In: 9th International Congress of ISDCI, St. Andrews Scotland. –*

Rakus, K.L.; Stet, R.J.M.; Savelkoul, H.F.J.; Irnazarow, I.; Pilarczyk, A.; Wiegertjes, G.F. (2003) Detection of polymorphism of MHC class II B genes in different carp lines. *In: 11th International Conference of teh EAFP, St. Julians Malta. –*

Savelkoul, H.F.J. (2003) [Hygienehypothese: allergie door een verstoord regulatornetwerk](http://library.wur.nl/wasp/bestanden/LUWPUBRD_00325498_A502_001.pdf). *Pulmonair (2003). - p. 16 - 21.*

Savelkoul, H.F.J.; Poel, J.J. van der; Parmentier, H.K. (2003) Natural antibodies in chickens are genetically determined and affect antigen-specific humoral and cellular responses. *In: 9th International Congress of ISDCI, St. Andrews Scotland; programme & abstracts. –*

Savelkoul, H.F.J. (2003) Is ons voedsel veilig? *Melange 3 (2003). - p. 15.*

Scharsack, J.P.; Saeij, J.P.J.; Zou, J.; Secombes, C.J.; Savelkoul, H.F.J.; Stet, R.J.M.; Wiegertjes, G.F. (2003) Functional aspects of tumour necrosis factor proteins in carp (Cyprinus carpio). *In: 9th International Congress of ISDCI, St. Andrews Scotland; programme & abstracts. –*

Sijben, J.W.C.; Klasing, K.C.; Schrama, J.W.; Parmentier, H.K.; Poel, J.J. van der; Savelkoul, H.F.J.; Kaiser, P. (2003) [Early in vivo cytokine genes expression in chickens after challenge with Salmonella typhimurium lipopolysaccharide and modulation by dietary n-3 polyunsaturated fatty acids](http://dx.doi.org/10.1016/S0145-305X(03)00031-4). *Developmental and Comparative Immunology 27 (2003). p. 611 - 619.*

Stet, R.J.M.; Hermsen, G.J.; Engelsma, M.Y.; Jukes, J.; Keulen, B. van; Kemenade, B.M.L. van; Shum, B.; Savelkoul, H.F.J. (2003) Novel immunoglobulin-like transcripts (nilts): an evolutionary conserved feature of innate immunity. *In: 9th International Congress of ISDCI, St. Andrews Scotland; programme & abstracts. –*

Stolte, H.H.; Leon, K.M.; Kramer, J.; Flik, G.; Savelkoul, H.F.J.; Kemenade, B.M.L. van (2003) Identification of immuno-regulatory molecules of fish by micro-array technology. *In: Wintermeeting Dutch Society for Immunology. –*

Wolkerstorfer, A.; Savelkoul, H.F.J.; Waard van der Spek, F.B. de; Neijens, H.J.; Meurs, T. van; Oranje, A.P. (2003) [Soluble E-selectin and soluble ICAM-1 levels as markers of the activity of atopic dermatitis in children](http://dx.doi.org/10.1034/j.1399-3038.2003.00057.x). *Pediatric Allergy and Immunology 14 (2003). p. 302 - 306.*

**2002**

Barrat, F.J.; Cua, D.J.; Boonstra, P.A.; Richards, D.F.; Crain, C.; Savelkoul, H.F.J.; Waal-Malefyt, R. de; Coffman, R.L.; Hawrylowicz, C.M.; O'Garra, A. (2002) [In vitro generation of interleukin-10-producing regulatory CD4(+) T cells is induced by immunosuppressive drugs and inhibited by T helper 1 (Th1)- and Th2-inducing cytokines](http://dx.doi.org/10.1084/jem.20011629). *The journal of experimental medicine 195 (2002). p. 603 - 616.*

Buljevac, D.; Flach, H.Z.; Hop, W.C.; Hijdra, D.; Laman, J.D.; Savelkoul, H.F.J.; Meche, F.G. van der; Doorn, P.A. van; Hintzen, R.Q. (2002) [Prospective study on the relationship between infections and multiple sclerosis exacerbations](http://dx.doi.org/10.1093/brain/awf098). *Brain 125 (2002). p. 952 - 960.*

Engelsma, M.Y.; Huising, M.O.; Muiswinkel, W.B. van; Flik, G.; Kwang, J.; Savelkoul, H.F.; Verburg van Kemenade, B.M.L. (2002) [Neuroendocrine-immune interactions in fish: a role for interleukin-1](http://dx.doi.org/10.1016/S0165-2427(02)00077-6). *Veterinary Immunology and Immunopathology 87: (2002). p. 467 - 479.*

Forlenza, M.; Savelkoul, H.F.J.; Wiegertjes, G.F. (2002) Innate immune responses to parasites in carp, Cyprinus carpio L *In: Abstracts of the Jaarcongres NVVI, Veldhoven, 2002. - [S.l.] : [s.n.], 2002 - p. np.*

Huising, M.O.; Stolte, H.H.; Kemenade, B.M.L. van; Savelkoul, H.F.J. (2002) Evolution of CXC chemokines in vertebrates. *In: Abstracts NVVI Jaarcongres 2003 : NVVI Jaarcongres 2003, Veldhoven, 2003. - [S.l.] : [s.n.], 2002 - p. np.*

Jurecka, P.; Irnazarow, I.; Pilarczyk, A.; Savelkoul, H.F.J.; Wiegertjes, G.F. (2002) Identification of carp transferrin cDNA : preliminary identification of polymorphism. *In: Abstracts of the International Scientific Conference about Current Problems of Animal Genetics and their Practical Application, Brno, Czech Republic, 2002. - [S.l.] : [s.n.], 2002. - XXth Genetic Days - p. np.*

Khan, N.A.; Khan, A.; Savelkoul, H.F.J.; Benner, R. (2002) [Inhibition of septic shock in mice by an oligopeptide from the beta-chain of human chorionic gonadotrophin hormone](http://dx.doi.org/10.1016/S0198-8859(01)00372-X). *Human Immunology 63 (2002). p. 1 - 7.*

Kruiswijk, C.P.; Hermsen, G.J.; Westphal, A.H.; Savelkoul, H.F.J.; Stet, R.J.M. (2002) [A novel functional class I lineage in zebrafish (Danio rerio), carp (Cyprinus carpio), and large barbus (Barbus intermedius)showing an unusual conservation of the peptide binding domains.](http://www.jimmunol.org/cgi/content/abstract/169/4/1936) *Journal of Immunology 169 (2002) - p. 1936 - 1947.*

Rakus, K.; Wiegertjes, G.F.; Stet, R.J.M.; Savelkoul, H.F.J.; Pilarczyk, A.; Irnazarow, I. (2002) Polymorphism of MHC class II B genes in different lines of common carp (Cyprinus carpio L.). *In: Abstracts of the International Scientific Conference about Current Problems of Animal Genetics and their Practical Application, Brno, Czech Republic, 2002. - [S.l.] : [s.n.], 2002. - XXth Genetic Days - p. np.*

Stolte, H.H.; Neijens, H.J.; Savelkoul, H.F.J. (2002) [Immuunregulatie bij de ontwikkeling van allergie op de jonge kinderleeftijd.](http://library.wur.nl/wasp/bestanden/LUWPUBRD_00123102_A502_001.pdf)
*Nederlands Tijdschrift voor Allergie, ISSN 1568-2498, 1 (2002) - p. 18 - 30.*

**2001**

Boonstra, A.; Barrat, F.J.; Crain, C.; Heath, V.L.; Savelkoul, H.F.J.; O'Garra, A. (2001) [1alpha,25-dihydroxyvitamin D3 has a direct effect on naive CD4+ T cells to enhance the development of Th2 cells](http://www.jimmunol.org/cgi/content/abstract/167/9/4974). *Journal of Immunology 167 (2001). p. 4974 - 4980.*

Boonstra, A.P.; Oudenaren, A. van; Baert, M.R.M.; Steeg, Steeg van; Leenen, P.J.; Horst, van der van der; Hoeijmakers, J.H.J.; Savelkoul, H.F.J.; Garssen, J. (2001) [Differential UVB-induced modulation of cytokine production in XPA, XPC, and CSB repair-deficient mice](http://dx.doi.org/10.1046/j.0022-202x.2001.01390.x). *Journal of Investigative Dermatology 117 (2001). p. 141 - 146.*

Cameron, S.B.; Nawijn, M.C.; Savelkoul, H.F.J.; Chow, A.W. (2001) [Regulation of helper T cell responses to staphylococcal superantigens](http://library.wur.nl/wasp/bestanden/LUWPUBRD_00336414_A502_001.pdf). *European Cytokine Network 12 (2001). p. 210 - 222.*

Engelsma, M.Y.; Stet, R.J.M.; Savelkoul, H.F.J.; Verburg-van Kemenade, B.M.L. (2001) A role for interleukin-1beta in neuroendocrine-immune interactions in carp, Cyprinus carpio L. *In: Abstracts Najaarsvergadering NVVI : Najaarvergadering Nederlandse Vereniging voor Immunologie, Noordwijkerhout, december 2001. - [S.l.] : [s.n.], 2001 - p. np.*

Guichelaar, T.; Hoek, C.; Taverne-Thiele, A.; Savelkoul, H.F.J.; Rombout, J.H.W.M.; Flik, G.; Verburg-van Kemenade, B.M.L.; Huising, M.O. (2001) Influences of osmotic shock on the efficacy of vaccine uptake and immune response in common carp (Cyprinus carpio L.). *In: Abstracts 8th Benelux congress of zoology : 8th Benelux congress of zoology, Nijmegen, November 2001. 2001 - p. np.*

Gysel, D. van; Koning, H.; Baert, M.R.; Savelkoul, H.F.J.; Neijens, H.J.; Oranje, A.P. (2001) [Clinico-immunological heterogeneity in Comel-Netherton syndrome](http://dx.doi.org/10.1159/000051607). *Dermatology 202 (2001). p. 99 - 107.*

Huising, M.O.; Guichelaar, T.; Savelkoul, H.F.J.; Rombout, J.H.W.M.; Flik, G.; Verburg-van Kemenade, B.M.L. (2001) Influences of osmotic shock on efficacy of vaccine uptake and immune response in common carp (Cyprinus carpio L.). *In: 5th Nordic Symposium on Fish Immunology : 5th Nordic Symposium on Fish Immunology, Sundvolden, Norway, June 2001. 2001 - p. 51.*

Huising, M.O.; Schooten, C. van; Schreurs, K.; Engelsma, M.Y.; Burg, E. van den; Savelkoul, H.F.J.; Flik, G.; Verburg-van Kemenade, B.M.L. (2001) Neuro-endocrine immune interactions during stress: hormones beyond the scope of the endocrine system. *In: Abstracts 8th Benelux Congress of Zoology : 8th Benelux Congress of Zoology, Nijmegen, November 2001. 2001 - p. np.*

Huising, M.O.; Guichelaar, T.; Hoek, C.; Taverne, A.; Rombout, J.H.W.M.; Flik, G.; Verburg-van Kemenade, B.M.L.; Savelkoul, H.F.J. (2001) Enhancement of vaccine uptake and immune response in carp after immersion vaccination. *In: Najaarsvergadering NVVI : Najaarsvergadering Nederlandse Vereniging voor Immunologie, Noordwijkerhout, december 2001. 2001 - p. 52.*

Khan, N.A.; Khan, A.; Savelkoul, H.F.J.; Benner, R. (2001) [Inhibition of diabetes in NOD mice by human pregnancy factor](http://dx.doi.org/10.1016/S0198-8859(01)00368-8). *Human Immunology 62 (2001). p. 1315 - 1323.*

Kruiswijk, C.P.; Hermsen, G.T.; Savelkoul, H.F.J.; Stet, R.J.M. (2001) A novel functional class I lineage in three cyprinid species: Barbus, carp and zebrafish. *In: Abstracts Nederlandse Vereniging voor Immunologie : NVVI Conference, Noordwijkerhout, december 2001. 2001 - p. np.*

Nawijn, M.C.; Dingjan, G.M.; Ferreira, R.; Lambrecht, B.N.; Karis, A.; Grosveld, F.; Savelkoul, H.F.J.; Hendriks, R.W. (2001) [Enforced expression of GATA-3 in transgenic mice inhibits Th1 differentiation and induces the formation of a T1/ST2-expressing Th2-committed T Cell compartment in vivo](http://www.jimmunol.org/cgi/reprint/167/2/724.pdf). *Journal of Immunology 167 (2001). p. 724 - 732.*

Stet, R.J.M.; Jukes, J.; Siebenga, J.; Verburg-van Kemenade, B.M.L.; Savelkoul, H.F.J.; Shum, B.; Parham, P.; Engelsma, M.Y. (2001) Natural cytotoxicity receptors: an evolutionary conserved feature of innate immunity. *In: Abstracts najaarsvergadering NVVI : Najaarsvergadering Nederlandse Vereniging voor Immunologie, Noordwijkerhout, december 2001. 2001 - p. np.*

Velden, V.H.J. van der; Laan, M.P.; Baert, M.R.M.; Waal-Malefyt, R. de; Neijens, H.J.; Savelkoul, H.F.J. (2001) [Selective development of a strong Th2 cytokine profile in high-risk children who develop atopy: risk factors and regulatory role of IFN-? IL-4 and IL-10](http://dx.doi.org/10.1046/j.1365-2222.2001.01176.x). *Clinical and Experimental Allergy 31 (2001). p. 997 - 1006.*

Verburg-van Kemenade, B.M.L.; Engelsma, M.Y.; Huising, M.O.; Muiswinkel, W.B. van; Savelkoul, H.F.J.; Kwang, J.; Flik, G. (2001) Neuro-endocrine immune interactions during stress: a role for cortisol and interleukin-1. *In: Abstracts 8th Benelux congress of zoology : 8th Benelux congress of zoology, Nijmegen, November 2001. 2001 - p. np.*

**2000**

Boonstra, A.; Oudenaren, A. van; Barendregt, B.; An, L.; Leenen, P.J.; Savelkoul, H.F.J. (2000) [UVB irradiation modulates systemic immune responses by affecting cytokine production of antigen-presenting cells](http://dx.doi.org/10.1093/intimm/12.11.1531). *International Immunology 12 (2000) - p. 1531 - 1538 .*

Laan, M.P.; Baert, M.R.M.; Bijl, A.M.H.; Vredendaal, A.E.C.M.; Waard-van der Spek, F.B. de; Oranje, A.P.; Savelkoul, H.F.J.; Neijens, H.J. (2000) [Markers for early sensitisation and inflammation in relation to allergic manifestations of atopic disease up to 2 years of age in 133 high-risk children](http://dx.doi.org/10.1046/j.1365-2222.2000.00856.x). *Clinical and Experimental Allergy 30 (2000). p. 944 - 953.*

Letter, M.A. de; Visser, L.H.; Meche, F.G. van der; Ang, W.; Savelkoul, H.F.J. (2000) [Distinctions between critical illness polyneuropathy and axonal Guillain-Barre sybdrome](http://dx.doi.org/10.1136/jnnp.68.3.397). *Journal of Neurology, Neurosurgery and Psychiatry 68 (2000). p. 397 - 398.*

Loveren, H. van; Boonstra, A.; Dijk, M. van; Fluitman, A.; Savelkoul, H.F.J.; Garssen, J. (2000) [UV exposure alters respiratory allergic responses in mice](http://dx.doi.org/10.1562/0031-8655(2000)072<0253:UEARAR>2.0.CO;2). *Photochemistry and Photobiology 72 (2000) - p. 253 - 259 .*

Megens-de Letter, M.A.C.J.; Doorn, P.A. van; Savelkoul, H.F.J.; Laman, J.D.; Schmitz, P.I.M.; Coul, A.A.W. op de; Visser, L.H.; Kross, M.; Teepen, J.L.J.M.; Meche, F.G.A. van der (2000) [Critical illness polyneuromyopathy (CIPNM): evidence for local immune activation by cytokine-expression in the muscle tissue](http://dx.doi.org/10.1016/S0165-5728(99)00252-0). *Journal of Neuroimmunology 106 (2000). p. 206 - 213.*

Savelkoul, H.F.J.; Neijens, H.J. (2000) [Immune responses during allergic sensitization and the development of atopy](http://dx.doi.org/10.1034/j.1398-9995.2000.00124.x). *Allergy 55 (2000). - p. 989 - 997.*

Savelkoul, H.F.J. (2000) [Immune parameters in high-risk atopic individuals during early childhood](http://ajrccm.atsjournals.org/cgi/content/full/162/3/S1/S100). *Am J Respir Crit Care Med 162 (2000). p. S100 - 104.*

**1999**

Boonstra, P.A.; Savelkoul, H.F.J. (1999) [Activity of T-cell subsets in allergic asthma](http://library.wur.nl/wasp/bestanden/LUWPUBRD_00336386_A502_001.pdf). *In: New and exploratory therapeutic agents for asthma. / Yeadon, M. Diamant, Z., - New York : Marcel Dekker, - p. 343 - 360.*

Boonstra, P.A.; Baert, M.R.M.; Velden, V.H.J. van der; Savelkoul, H.F.J. (1999) [Flowcytometrische analyse van intracellulaire cytokinen](http://library.wur.nl/wasp/bestanden/LUWPUBRD_00336387_A502_001.pdf). *In: Flowcytometrische immunodiagnostiek. / Dongen, J.J.M. van Hooijkaas, H., . - Rotterdam : Erasmus Universiteit, p. 83 - 94.*

Heuven-Nolsen, D. van; Kimpe, S.J. de; Muis, T.; Ark, I. van; Savelkoul, H.F.J.; Beems, R.B.; Oosterhout, A.J.M. van; Nijkamp, F.P. (1999) [Opposite role of interferon-gamma and interleukin-4 on the regulation of blood pressure in mice](http://dx.doi.org/10.1006/bbrc.1998.8742). *Biochemical and Biophysical Research Communications 254 (1999). p. 816 - 820.*

Megens-de Letter, M.A.C.J.; Visser, L.H.; Doorn, P.A. van; Savelkoul, H.F.J. (1999) [Cytokines in the muscle tissue of idiopathic inflammatory myopathies: implications for immunopathogenesis and therapy](http://library.wur.nl/wasp/bestanden/LUWPUBRD_00336384_A502_001.pdf). *European Cytokine Network 10 (1999) p. 471 - 478.*

**1998**

Baert, M.R.M.; Savelkoul, H.F.J. (1998) [Intracellular staining for cytokines in blood lymphocytes of young children with allergic disease](http://library.wur.nl/wasp/bestanden/LUWPUBRD_00336370_A502_001.pdf). *IQ Press 8 (1998). - p. 7 - 9.*

Bianchi, A.T.J.; Moonen-Leusen, H.W.M.; Milligen, F.J. van; Savelkoul, H.F.J.; Zwart, R.J.; Kimman, T.G. (1998) [A mouse model to study immunity against pseudorabies virus infection: Significance of CD4+ and CD8+ cells in protective immunity](http://dx.doi.org/10.1016/S0264-410X(98)00044-9). *Vaccine 16, 1998, 1550-1558*

Hagen, T.L. ten; Vianen, W. van; Savelkoul, H.F.J.; Heremans, H.; Buurman, W.A.; Bakker-Woudenberg, I.A. (1998) [Involvement of T cells in enhanced resistance to Klebsiella pneumoniae septicemia in mice treated with liposome-encapsulated muramyl tripeptide phosphatidylethanolamine or gamma interferon](http://iai.asm.org/cgi/content/full/66/5/1962). *Infection and Immunity 66 (1998). p. 1962 - 1967.*

Hofstra, C.L.; Ark, I. van; Savelkoul, H.F.J.; Cruikshank, W.W.; Nijkamp, F.P.; Oosterhout, A.J.M. van (1998) [V-beta-8+ T lymphocytes are essential in the regulation of airway hyperresponsiveness and bronchial eosinophilia but not in allergen-specific IgE in a murine model of allergic asthma](http://dx.doi.org/10.1046/j.1365-2222.1998.00387.x). *Clinical and Experimental Allergy 28 (1998)12. p. 1571 - 1580.*

Laan, M.P.; Tibbe, G.J.M.; Oranje, A.P.; Bosmans, E.P.E.; Neijens, H.J.; Savelkoul, H.F.J. (1998) [CD4+ cells proliferate after peanut-extract-specific and CD8+ cells proliferate after polyclonal stimulation of PBMC of children with atopic dermatitis](http://dx.doi.org/10.1046/j.1365-2222.1998.00168.x). *Clinical and Experimental Allergy 28 (1998). p. 35 - 44.*

Laan, M.P.; Koning, H.; Baert, M.R.M.; Oranje, A.P.; Buurman, W.A.; Savelkoul, H.F.J. (1998) [Levels of soluble intracellular adhesion molecule-1, soluble E-selectin, tumor necrosis factor-alpha and soluble tumor necrosis factor receptor p55 and p75 in atopic children](http://library.wur.nl/wasp/bestanden/LUWPUBRD_00336366_A502_001.pdf). *Allergy 53 (1998). - p. 51 - 58.*

Laan, M.P.; Baert, M.R.M.; Vredendaal, A.E.C.M.; Savelkoul, H.F.J. (1998) [Differential mRNA expression and production of interleukin-4 and interferon-gamma in stimulated peripheral blood mononuclear cells of house-dust mite-allergic patients](http://library.wur.nl/wasp/bestanden/LUWPUBRD_00336367_A502_001.pdf). *European Cytokine Network 9 (1998). p. 75 - 84.*

Oosterhout, A.J.M. van; Esch, B. van; Hofman, G.; Hofstra, C.L.; Ark, I. van; Nijkamp, F.P.; Kapsenberg, M.L.; Savelkoul, H.F.J.; Weller, F.R. (1998) [Allergen immunotherapy inhibits airway eosinophilia and hyperresponsiveness associated with decreased IL-4 production by lymphocytes in a murine model of allergic asthma](http://ajrcmb.atsjournals.org/cgi/content/full/19/4/622). *American Journal of Respiratory Cell and Molecular Biology 19 (1998). p. 622 - 628.*

Savelkoul, H.F.J.; Laan, M.P.; Baert, M.R.M.; Oranje, A.P.; Neijens, H.J. (1998) [Peanut-specific responses in young children with atopic dermatitis: symposium 10 years ALASTAT](http://library.wur.nl/wasp/bestanden/LUWPUBRD_00338608_A502_001.pdf). *In: DPC symposia and meetings in the Benelux 1998. –*

Velden, V.H.J. van der; Savelkoul, H.F.J.; Versnel, M.A. (1998) [Bronchial epithelium: morphology, function and pathophysiology in asthma](http://library.wur.nl/wasp/bestanden/LUWPUBRD_00336362_A502_001.pdf). *European Cytokine Network 9 (1998). p. 585 - 597.*

Velden, V.H.J. van der; Naber, B.A.E.; Wierenga-Wolf, A.F.; Debets, R.; Savelkoul, H.F.J.; Overbeek, S.E.; Hoogsteden, H.C.; Versnel, M.A. (1998) [Interleukin 4 receptors on human bronchial epithelial cells.An in vivo and in vitro analysis of expression and function](http://dx.doi.org/10.1006/cyto.1998.0365). *Cytokine 10 (1998). p. 803 - 813.*

Wolkerstorfer, A.; Laan, M.P.; Savelkoul, H.F.J.; Oudesluys-Murphy, A.M.; Sukhai, R.N.; Oranje, A.P. (1998) [De rol van adhesiemoleculen bij atopisch eczeem](http://library.wur.nl/wasp/bestanden/LUWPUBRD_00336360_A502_001.pdf). *Nederlands Tijdschrift voor Dermatologie en Venereologie 8 (1998). p. 38 - 39.*

Wolkerstorfer, A.; Laan, M.P.; Savelkoul, H.F.J.; Neijens, H.J.; Mulder, P.G.; Oudesluys-Murphy, A.M.; Sukhai, R.N.; Oranje, A.P. (1998) [Soluble E-selectin, other markers of inflammation and disease severity in children with atopic dermatitis](http://dx.doi.org/10.1046/j.1365-2133.1998.02120.x). *The British journal of dermatology 138 (1998). p. 431 - 435.*

**1997**

Arkel, C. van; Hopstaken, C.M.; Zurcher, C.; Bos, N.A.; Kroese, F.G.M.; Savelkoul, H.F.J.; Benner, R.; Radl, J. (1997) [Monoclonal gammopathies in aging mu, kappa-transgenic mice: involvement of the B-1 cell lineage](http://library.wur.nl/wasp/bestanden/LUWPUBRD_00336353_A502_001.pdf). *European journal of immunology 27 (1997). p. 2436 - 2440.*

Boonstra, A.; Savelkoul, H.F.J. (1997) [The role of cytokines in ultraviolet-B induced immunosuppression](http://library.wur.nl/wasp/bestanden/LUWPUBRD_00336352_A502_001.pdf). *European Cytokine Network 8 (1997). p. 117 - 123.*

Drabek, D.; Raguz, S.; Wit, T.P.M. de; Dingjan, G.M.; Savelkoul, H.F.J.; Grosveld, F.; Hendriks, R.W. (1997) [Correction of the X-linked immunodeficiency phenotype by transgenic expression of human Bruton Tyrosine kinase under the control of the class II major histocompatibility complex Ea locus control region](http://dx.doi.org/10.1073/pnas.94.2.610). *Proceedings of the National Academy of Sciences of the United States of America 94 (1997). p. 610 - 615.*

Halteren, A.G. van; Cammen, M.J. van der; Cooper, D.; Savelkoul, H.F.J.; Kraal, G.; Holt, P.G. (1997) [Regulation of antigen-specific IgE, IgG1, and mast cell responses to ingested allergen by mucosal tolerance induction](http://www.jimmunol.org.ezproxy.library.wur.nl/cgi/content/abstract/159/6/3009). *Journal of Immunology 159 (1997). p. 3007 - 3015.*

Halteren, A.G.S. van; Cammen, M.J.F. van der; Biewenga, J.; Savelkoul, H.F.J.; Kraal, G. (1997) [IgE and mast cell responses on intestinal allergen exposure: a murine model to study the onset of food allergy](http://library.wur.nl/wasp/bestanden/LUWPUBRD_00336350_A502_001.pdf). *Journal of Allergy and Clinical Immunology 99 (1997). p. 94 - 99.*

Hessel, E.M.; Oosterhout, A.J.M. van; Ark, I. van; Esch, B. van; Hofman, G.; Loveren, H. van; Savelkoul, H.F.J.; Nijkamp, F.P. (1997) [Development of airway hyperresponsiveness is dependent on interferon-? and independent of eosinophil infiltration](http://library.wur.nl/wasp/bestanden/LUWPUBRD_00336348_A502_001.pdf). *American Journal of Respiratory Cell and Molecular Biology 16 (1997). p. 325 - 334.*

Janossy, T.; Vizler, C.; Ocsovski, I.; Tibbe, G.J.; Pipis, J.; Savelkoul, H.F.J.; Vegh, P.; Benner, R. (1997) [Impaired T cell functions preceding lymphoproliferative disorders in mice neonatally tolerized to transplantation antigens](http://library.wur.nl/wasp/bestanden/LUWPUBRD_00336347_A502_001.pdf). *Acta chirurgica Hungarica 36 (1997). p. 150 - 151.*

Koning, H.; Neijens, H.J.; Baert, M.R.M.; Oranje, A.P.; Savelkoul, H.F.J. (1997) [T cell subsets and cytokines in allergic and non-allergic children. II. Analysis of IL-5 and IL-10 mRNA expression and protein production](http://dx.doi.org/10.1006/cyto.1996.0185). *Cytokine 9 (1997). p. 427 - 436.*

Koning, H.; Neijens, H.J.; Baert, M.R.M.; Oranje, A.P.; Savelkoul, H.F.J. (1997) [T cell subsets and cytokines in allergic and non allergic children. I. Analysis of IL-4, IFN-? and IL-13 mRNA expression and protein production](http://dx.doi.org/10.1006/cyto.1996.0184). *Cytokine 9 (1997). p. 416 - 426.*

Kornelisse, R.F.; Hack, C.E.; Savelkoul, H.F.J.; Pouw-Kraan, T.C.T.M. van der; Hop, W.C.J.; Mierlo, G. van; Suur, M.H.; Neijens, H.J.; Groot, R. de (1997) [Intrathecal production of interleukin-12 and gamma-interferon in patients with bacterial meningitis](http://iai.asm.org/cgi/content/abstract/65/3/877). *Infection and Immunity 65 (1997). p. 877 - 881.*

Leenaars, P.P.A.M.; Savelkoul, H.F.J.; Hendriksen, C.F.M.; Rooijen, N. van; Claassen, E. (1997) [Increased adjuvant efficacy in stimulation of antibody responses after macrophage elimination in vivo](http://dx.doi.org/10.1111/j.1365-2567.1997.00337.x). *Immunology 90 (1997). p. 337 - 343.*

Maas, A.; Dingjan, G.M.; Savelkoul, H.F.J.; Kinnon, C.; Grosveld, F.; Hendriks, R.W. (1997) [The X-linked immunodeficiency defect in the mouse is corrected by expression of human Bruton?s tyrosine kinase from a yeast artificial chromosome transgene](http://library.wur.nl/wasp/bestanden/LUWPUBRD_00336342_A502_001.pdf), *European journal of immunology 27 (1997). p. 2180 - 2187.*

Pathak, S.S.; Tibbe, G.J.M.; Savelkoul, H.F.J. (1997) [Determination of antibody affinity and affinity distributions.Chapter 13.9](http://library.wur.nl/wasp/bestanden/LUWPUBRD_00336339_A502_001.pdf). *In: Manual of immunological methods. / Lefkovits, I., . - London : Academic Press, - p. 1078 - 1093.*

Pathak, S.S.; Savelkoul, H.F.J. (1997) [Biosensors in immunology: the story so far](http://dx.doi.org/10.1016/S0167-5699(97)01124-9). *Immunology Today 18 (1997). p. 464 - 467.*

Pathak, S.S.; Oudenaren, A. van; Savelkoul, H.F.J. (1997) [Quantitation of immunoglobulin concentration by ELISA.Chapter 13.8](http://library.wur.nl/wasp/bestanden/LUWPUBRD_00336341_A502_001.pdf). *In: Manual of immunological methods. / Lefkovits, I., . - London : Academic Press, - p. 1056 - 1074.*

Pathak, S.S.; Savelkoul, H.F.J. (1997) Quantitation of concentration and functional affinity of immunoglobulins with special reference to Terasaki-ELISA. *Trivandrum : CSI, 1997 (Life Science Advances ) - p. 92.*

Savelkoul, H.F.J.; Claassen, E.; Benner, R. (1997) [Outlook. Chapter 13.11](http://library.wur.nl/wasp/bestanden/LUWPUBRD_00336333_A502_001.pdf). *In: Manual of immunological methods. / Lefkovits, I., . - London : Academic Press, - p. 1104 - 1105.*

Savelkoul, H.F.J.; Claassen, E.; Benner, R. (1997) [Introduction. Chapter 13.1](http://library.wur.nl/wasp/bestanden/LUWPUBRD_00336334_A502_001.pdf). *In: Manual of immunological methods. / Lefkovits, I., . - London : Academic Press, - p. 979 - 980.*

Savelkoul, H.F.J. (1997) [Immunopathology in childhood allergy](http://library.wur.nl/wasp/bestanden/LUWPUBRD_00336335_A502_001.pdf). *In: DPC Allergy Symposium. - p. 5 - 6.*

Savelkoul, H.F.J. (1997) [Cytokines and asthma: basic principles and clinical applications](http://library.wur.nl/wasp/bestanden/LUWPUBRD_00336336_A502_001.pdf). *In: Proc.XXVII Ann Conf Indian Pharmacol Soc. p. 1 - 8.*

Savelkoul, H.F.J. (1997) [Alginate encapsulation of cytokine gene-transfected cells.Chapter 13.5](http://library.wur.nl/wasp/bestanden/LUWPUBRD_00336337_A502_001.pdf). *In: Manual of immunological methods. / Lefkovits, I. - London : Academic Press, - p. 1030 - 1035.*

Savelkoul, H.F.J. (1997) [Recombinante IgE en IgG4 antistoffen. Proefschriftbespreking J Schuurman](http://library.wur.nl/wasp/bestanden/LUWPUBRD_00336338_A502_001.pdf). *Pulmonair 4 (1997). p. 10 - 12.*

Wolkerstorfer, A.; Neijens, H.J.; Savelkoul, H.F.J.; Oudesluys-Murphy, A.M.; Sukhai, R.N.; Oranje, A.P. (1997) [ETAC: de eerste resultaten](http://library.wur.nl/wasp/bestanden/LUWPUBRD_00336332_A502_001.pdf). *Modern Medicine 7 (1997). p. 28 - 29.*

**1996**

Debets, R.; Savelkoul, H.F.J. (1996) [Cytokines as cellular communicators](http://library.wur.nl/wasp/bestanden/LUWPUBRD_00336319_A502_001.pdf). *Mediators of Inflammation 5 (1996). p. 417 - 423.*

Gijbels, M.J.J.; Zurcher, C.; Kraal, G.; Elliott, G.R.; HogenEsch, H.; Schijff, G.; Savelkoul, H.F.J.; Bruijnzeel, P.L.B. (1996) [Pathogenesis of skin lesions in mice with chronic proliferative dermatitis (cpdm/cpdm)](http://library.wur.nl/wasp/bestanden/LUWPUBRD_00336318_A502_001.pdf). *The American journal of pathology 148 (1996). p. 941 - 950.*

Koning, H.; Baert, M.R.M.; Oranje, A.P.; Savelkoul, H.F.J.; Neijens, H.J. (1996) [Development of immune functions related to allergic mechanisms in young children](http://library.wur.nl/wasp/bestanden/LUWPUBRD_00336317_A502_001.pdf). *Pediatric Research 40 (1996). p. 363 - 375.*

Kornelisse, R.F.; Savelkoul, H.F.J.; Mulder, P.H.G.; Suur, M.H.; Straaten, P.J.C. van der; Heijden, A.J. van der; Sukhai, R.N.; HÃƒÂ¤hlen, K.; Neijens, H.J.; Groot, R. de (1996) [Interleukin-10 and soluble tumor necrosis factor receptors in cerebrospinal fluid of children with bacterial meningitis](http://library.wur.nl/wasp/bestanden/LUWPUBRD_00336315_A502_001.pdf). *Journal of Infectious Diseases 173 (1996). p. 1498 - 1502.*

Kornelisse, R.F.; Hazalzet, J.A.; Savelkoul, H.F.J.; Hop, W.C.J.; Suur, M.H.; Borsboom, A.N.J.; Risseeuw-Appel, I.M.; Voort, E. van der; Neijens, H.J.; Groot, R. de (1996) [The relationship between plasminogen activation inhibitor-1 and proinflammatory and counterinflammatory mediators in children with meningococcal septic shock](http://library.wur.nl/wasp/bestanden/LUWPUBRD_00336316_A502_001.pdf). *Journal of Infectious Diseases 173 (1996). p. 1148 - 1156.*

Noort, W.A.; Benner, R.; Savelkoul, H.F.J. (1996) [Differential effectiveness of anti-CD8 treatment on ongoing graft-versus-host reactions in mice](http://dx.doi.org/10.1016/S0966-3274(96)80017-7), *Transplant Immunology 4 (1996). p. 198 - 202.*

Pathak, S.S.; Erkeland, S.; Tibbe, G.J.M.; Savelkoul, H.F.J. (1996) Biosensors and their potential applications
*In: Residues of veterinary drugs in food.Proc.EuroResidue III conference.Veldhoven, The Netherlands, 6-8 May 1996.Haagsma N, Ruiter A.(eds.) ISBN 90-6159-023-X. - Veldhoven, The Netherlands : [s.n.], - p. 113 - 125.*

Savelkoul, H.F.J.; Sminia, T. (1996) [Humorale immuunreacties](http://library.wur.nl/wasp/bestanden/LUWPUBRD_00336310_A502_001.pdf). *In: Medische immunologie / Benner, R. Dongen, J.J.M. van Ewijk, W. van Haaijman, J.J., . - Utrecht : Wetenschappelijke Uitgeverij Bunge, - p. 270 - 306.*

Savelkoul, H.F.J.; Ommen, R. van (1996) [Role of IL-4 in persistent IgE formation](http://library.wur.nl/wasp/bestanden/LUWPUBRD_00336311_A502_001.pdf). *European Respiratory Journal 9 (1996). p. 67s - 71s.*

Savelkoul, H.F.J. (1996) [Book review of: Centner J.and De Weck A.L.(eds.), Altlas of immuno-allergology, an illustrated primer for health care professionals.3rd ed.Gottingen: Hogrefe & Huber Publ, 1995](http://library.wur.nl/wasp/bestanden/LUWPUBRD_00336312_A502_001.pdf). *Mediators of Inflammation 5 (1996). p. 235 - 236.*

Savelkoul, H.F.J.; Sminia, T.F. (1996) [Humorale Immuunreacties](http://library.wur.nl/wasp/bestanden/LUWPUBRD_00336320_A502_001.pdf). *In: Medische immunologie. / Benner, R. ; Dongen, J.J.M. van ; Ewijk, W. van, . - Utrecht : Bunge, p. 269 - 299.*

Vossen, A.C.T.M.; Tibbe, G.J.M.; Buurman, W.A.; Benner, R.; Savelkoul, H.F.J. (1996) [Soluble tumour necrosis factor receptor release after anti-CD3 monoclonal antibody treatment in mice is independent of tumour necrosis factor-a release](http://library.wur.nl/wasp/bestanden/LUWPUBRD_00336308_A502_001.pdf). *European Cytokine Network 7 (1996). p. 751 - 755.*

VÃƒ, P.; Vizler, C.; JÃƒÂ¡nossy, T.; Savelkoul, H.F.J.; Nyirati, I.; Benner, R. (1996) [Induction and analysis of lethal graft-versus-host disease in tolerant mice](http://library.wur.nl/wasp/bestanden/LUWPUBRD_00336309_A502_001.pdf). *Transplantation Proceedings 28 (1996). p. 1241 - 1243.*

**1995**

Baert, M.R.M.; Koning, H.; Neijens, H.J.; Oranje, A.P.; Groot, R. de; Savelkoul, H.F.J. (1995) [Role of the immune system in allergic children](http://library.wur.nl/wasp/bestanden/LUWPUBRD_00336300_A502_001.pdf). *Pediatric Allergy and Immunology 6 (1995). p. 27 - 30.*

Buijs, J.; Egbers, M.W.E.C.; Lokhorst, W.H.; Savelkoul, H.F.J.; Nijkamp, F.P. (1995) [Toxocara-induced eosinophilic inflammation](http://library.wur.nl/wasp/bestanden/LUWPUBRD_00336299_A502_001.pdf). *American journal of respiratory and critical care medicine 151 (1995). p. 873 - 878.*

Hagen, T.L.M. ten; Vossen, A.C.T.M.; Vianen, W. van; Tibbe, G.J.M.; Savelkoul, H.F.J.; Heremans, H.; Bakker-Woudenberg, I.A.J.M. (1995) [Enhancement of nonspecific resistance by liposome-encapsulated immunomodulators does not affect skin graft rejection in mice](http://library.wur.nl/wasp/bestanden/LUWPUBRD_00336298_A502_001.pdf). *Transplantation 60 (1995). p. 1211 - 1214.*

Hamel, M.E.; Eynon, E.E.; Savelkoul, H.F.J.; Oudenaren, A. van; Kruisbeek, A.M. (1995) [Activation and re-activation potential of T cells responding to staphylococcal enterotoxin B](http://library.wur.nl/wasp/bestanden/LUWPUBRD_00336297_A502_001.pdf). *International Immunology 7 (1995). p. 1065 - 1077.*

Heijden, Ph.J. van der; Cornelissen, J.B.W.J.; Breedland, E.G.; Savelkoul, H.F.J.; Bianchi, A.T.J. (1995) [Cytokine response in the intestinal lamina propria of mice after infection with fasciola hepatica](http://library.wur.nl/wasp/bestanden/LUWPUBRD_00336296_A502_001.pdf). *In: Advances in mucosal immunity. / Mestecky, J. Russell, M.W. Jackson, S. Michalek, S.M. TlaskalovÃƒÂ¡-HogenovÃƒÂ¡, H. terzl, J., . - [s.l.] : [s.n.], p. 979 - 982.*

Hessel, E.M.; Oosterhout, A.J.M. van; Hofstra, C.L.; Bie, J.J. de; Garssen, J.; Loveren, H. van; Verheyen, A.K.C.P.; Savelkoul, H.F.J.; Nijkamp, F.P. (1995) [Bronchoconstriction and airway hyperresponsiveness after ovalbumin inhalation in sensitized mice](http://dx.doi.org/10.1016/S0014-2999(95)80099-9). *European Journal of Pharmacology 293 (1995). p. 401 - 412.*

Koning, H.; Baert, M.R.M.; Groot, R. de; Neijens, H.J.; Savelkoul, H.F.J. (1995) [Analysis of cytokine gene expression in stimulated T cells of small children by semi-quantitative PCR](http://library.wur.nl/wasp/bestanden/LUWPUBRD_00336294_A502_001.pdf). *Mediators of Inflammation 4 (1995). p. 196 - 204.*

Laan, M.P.; Koning, H.; Oranje, A.P.; Baert, M.R.M.; Groot, R. de; Neijens, H.J.; Savelkoul, H.F.J. (1995) [Peanut-allergen specific stimulation of PBMC in children with atopic dermatitis](http://library.wur.nl/wasp/bestanden/LUWPUBRD_00336293_A502_001.pdf). *In: XVI European Congress of Allergology and Clinical Immunology.ECACI '95. - Madrid : [s.n.], - p. 743 - 747.*

Lang, M.S.; Hovenkamp, E.; Savelkoul, H.F.J.; Knegt, P.; Ewijk, W. van (1995) [Immunotherapy with monoclonal antibodies directed against the immunosuppressive domain of p15E inhibits tumour growth](http://library.wur.nl/wasp/bestanden/LUWPUBRD_00336292_A502_001.pdf). *Clinical and experimental immunology 102 (1995). p. 468 - 475.*

Noort, W.A.; Benner, R.; Savelkoul, H.F.J. (1995) [Anti-CD4 IgG2a monoclonal antibody treatment prevents the expansion of T cells in the spleen during murine graft-vs-host disease](http://library.wur.nl/wasp/bestanden/LUWPUBRD_00336291_A502_001.pdf). *Transplantation Proceedings 27 (1995). p. 384 - 386.*

Oosterhout, A.J.M. van; Savelkoul, H.F.J. (1995) [Interleukine 5 as a drug target in allergy and asthma](http://dx.doi.org/10.1016/S0165-6147(00)88974-9). *Trends in Pharmacological Sciences 16 (1995). p. 37.*

Oosterhout, A.J.M. van; Ark, I. van; Folkerts, G.; Linde, H.J. van der; Savelkoul, H.F.J.; Verheyen, A.K.C.P.; Nijkamp, F.P. (1995) [Antibody to interleukin-5 inhibits virus-induced airway hyperresponsiveness to histamine in guinea pigs](http://library.wur.nl/wasp/bestanden/LUWPUBRD_00336290_A502_001.pdf). *Am J Respir Crit Care Med 151 (1995). p. 177 - 183.*

Samsom, J.N.; Langermans, J.A.M.; Savelkoul, H.F.J.; Furth, R. van (1995) [Tumour necrosis factor, but not interferon-gamma is essential for acquired resistance to Listeria monocytogenes during a secondary infection in mice](http://library.wur.nl/wasp/bestanden/LUWPUBRD_00336288_A502_001.pdf). *Immunology 86: (1995). p. 256 - 262.*

Schilizzi, B.M.; Savelkoul, H.F.J.; Jonge, M.W.A. de; The, T.H.; Leij, L. de (1995) [Impaired antigen-specific B-cell response and altered splenic microstructure in mice following continuous administration of IL-4 in vivo](http://library.wur.nl/wasp/bestanden/LUWPUBRD_00336287_A502_001.pdf). *Scandinavian journal of immunology 41 (1995).. 467 - 474.*

Vossen, A.C.T.M.; Tibbe, G.J.M.; Kroos, M.J.; Winkel, J.G.J. van de; Benner, R.; Savelkoul, H.F.J. (1995) [Fc receptor binding of anti-CD3 monoclonal antibodies is not essential for immunosuppression, but triggers cytokine-related side effects](http://library.wur.nl/wasp/bestanden/LUWPUBRD_00336285_A502_001.pdf). *European journal of immunology 25 (1995). p. 1492 - 1496.*

Vossen, A.C.T.M.; Tibbe, G.J.M.; Benner, R.; Savelkoul, H.F.J. (1995) [T lymphocyte and cytokine-directed strategies for inhibiting skin allograft rejection in mice](http://library.wur.nl/wasp/bestanden/LUWPUBRD_00336286_A502_001.pdf). *Transplantation Proceedings 27 (1995). p. 380 - 382.*

Wilsem, E.G. van; BrevÃƒ, J.; Savelkoul, H.; Claessen, A.; Scheper, R.J.; Kraal, G. (1995) [Oral tolerance is determined at the level of draining lymph nodes](http://library.wur.nl/wasp/bestanden/LUWPUBRD_00336284_A502_001.pdf). *Immunobiology 194 (1995). p. 403 - 414.*

**1994**

Debets, R.; Savelkoul, H.F.J. (1994) [Cytokine antagonists and their potential therapeutic use](http://dx.doi.org/10.1016/0167-5699(94)90187-2). *Immunology Today 15 (1994). p. 455 - 458.*

Delens, N.; Torreele, E.; Savelkoul, H.; Baetselier, P. de; Bouwens, L. (1994) [Tumor-derived transforming growth factor-beta 1 and interleukin-6 are chemotactic for lymphokine-activated killer cells](http://library.wur.nl/wasp/bestanden/LUWPUBRD_00336275_A502_001.pdf). *International Journal of Cancer 57 (1994). p. 696 - 700.*

Knulst, A.C.; Tibbe, G.J.M.; Noort, W.A.; Bril-Bazuin, C.; Benner, R.; Savelkoul, H.F.J. (1994) [Prevention of lethal graft-versus-host disease in mice by monoclonal antibodies directed against T cells or their subsets.I.Evidence for the induction of a state of tolerance based on suppression](http://library.wur.nl/wasp/bestanden/LUWPUBRD_00336272_A502_001.pdf). *Bone Marrow Transplantation 13 (1994). p. 293 - 301.*

Knulst, A.C.; Tibbe, G.J.M.; Bril-Bazuin, C.; Breedland, E.G.; Oudenaren, A. van; Benner, R.; Savelkoul, H.F.J. (1994) [Cytokine detection and modulation in acute graft vs.host disease in mice](http://library.wur.nl/wasp/bestanden/LUWPUBRD_00336273_A502_001.pdf). *Mediators of Inflammation 3 (1994). p. 33 - 40.*

Knulst, A.C.; Noort, W.A.; Tibbe, G.J.M.; Benner, R.; Savelkoul, H.F.J. (1994) [Prevention of lethal graft-versus-host disease in mice by monoclonal antibodies directed to T cells or their subsets.II.Differential effectiveness of IgG2a and IgG2b isotypes of anti-CD3 and anti-CD4 moAb](http://library.wur.nl/wasp/bestanden/LUWPUBRD_00336274_A502_001.pdf). *Bone Marrow Transplantation 14 (1994). p. 535 - 543.*

Kraal, G.; Schornagel, K.; Savelkoul, H.; Maruyama, T. (1994) [Activation of high endothelial venules in peripheral lymph nodes.The involvement of interferon-gamma](http://library.wur.nl/wasp/bestanden/LUWPUBRD_00336271_A502_001.pdf), *International Immunology 6 (1994). p. 1195 - 1201.*

Mink, C.M.; Esch, W.J.E. van; Savelkoul, H.F.J.; Loveren, H. van; Bernadina, W.E.; Ruitenberg, E.J. (1994) [Role of interleukin-4 and interleukin-5 in the gut immune response to Trichinella spiralis in mice](http://library.wur.nl/wasp/bestanden/LUWPUBRD_00336270_A502_001.pdf). *In: Trichinellosis.Proc.8th International Conference on Trichinellosis. / Campbell, W.C. Pozio, E. Bruschi, F., . - Rome, Italy : Istituto Superiore di Sanita Press, - p. 255 - 260.*

Ommen, R. van; Vredendaal, A.E.C.M.; Savelkoul, H.F.J. (1994) [Suppression of polyclonal and antigen-specific murine IgG1, but not IgE responses by neutralizing interleukin-6 in vivo](http://library.wur.nl/wasp/bestanden/LUWPUBRD_00336265_A502_001.pdf). *European journal of immunology 24 (1994). p. 1396 - 1403.*

Ommen, R. van; Vredendaal, A.E.C.M.; Savelkoul, H.F.J. (1994) [Secondary IgE responses in vivo are predominantly generated via?1e-double positive B cells](http://library.wur.nl/wasp/bestanden/LUWPUBRD_00336266_A502_001.pdf). *Scandinavian journal of immunology 40 (1994). p. 491 - 501.*

Ommen, R. van; Vredendaal, A.E.C.M.; Savelkoul, H.F.J. (1994) [Prolonged in vivo IL-4 treatment inhibits antigen-specific IgG1 and IgE formation](http://library.wur.nl/wasp/bestanden/LUWPUBRD_00336267_A502_001.pdf). *Scandinavian journal of immunology 40 (1994). p. 1 - 9.*

Ommen, R. van; Vredendaal, A.E.C.M.; Gooyer, M. de; Oudenaren, A. van; Savelkoul, H.F.J. (1994) [The effect of IFN-? alum and complete Freund adjuvant on TNP-KLH induced IgG1, IgE and IgG2a responses in mice](http://library.wur.nl/wasp/bestanden/LUWPUBRD_00336268_A502_001.pdf). *Mediators of Inflammation 3 (1994). p. 387 - 392.*

Ommen, R. van; Savelkoul, H.F.J. (1994) [Prolonged IL-4 treatment decreases the TNP-specific memory formation for IgG1](http://library.wur.nl/wasp/bestanden/LUWPUBRD_00336269_A502_001.pdf). *In: In vivo immunology: regulatory processes during lymphopoiesis and immunopoiesis. / Heinen, E., . - New York : Plenum Publ., - p. 39 - 43.*

Savelkoul, H.F.J.; Vossen, A.C.T.M.; Breedland, E.G.; Tibbe, G.J.M. (1994) [Semi-preparative purification and validation of monoclonal antibodies for immunotherapy in mice](http://dx.doi.org/10.1016/0022-1759(94)90376-X). *Journal of Immunological Methods 172 (1994). - p. 33 - 42.*

Savelkoul, H.F.J.; Pathak, S.S. (1994) [The IAsys biosensor for affinity measurements of antibodies in immune responses](http://library.wur.nl/wasp/bestanden/LUWPUBRD_00336262_A502_001.pdf). *Fisons Instruments Fusion 7 (1994). - p. 10 - 11.*

Savelkoul, H.F.J.; Ommen, R. van; Vossen, A.C.T.M.; Breedland, E.G.; Coffman, R.L.; Oudenaren, A. van (1994) [Modulation of systemic cytokine levels by implantation of alginate encapsulated cells](http://dx.doi.org/10.1016/0022-1759(94)90394-8). *Journal of Immunological Methods 170 (1994). - p. 185 - 196.*

Savelkoul, H.F.J. (1994) [De regulatie van de IgE antistofvorming](http://library.wur.nl/wasp/bestanden/LUWPUBRD_00336264_A502_001.pdf). *De allergie bode (1994)Issue. - p. 5 - 8.*

Vossen, A.C.T.M.; Tibbe, G.J.M.; Oudenaren, A. van; Vredendaal, A.E.C.M.; Benner, R.; Savelkoul, H.F.J. (1994) [A rat anti-mouse CD3 monoclonal antibody induces long-term skin allograft survival without inducing side-effects](http://library.wur.nl/wasp/bestanden/LUWPUBRD_00336258_A502_001.pdf). *Transplantation Proceedings 26 (1994). p. 3157 - 3158.*

Vossen, A.C.T.M.; Savelkoul, H.F.J. (1994) [Cytokines in clinical and experimental transplantation](http://library.wur.nl/wasp/bestanden/LUWPUBRD_00336259_A502_001.pdf). *Mediators of Inflammation 3 (1994). p. 403 - 410.*

Vossen, A.C.T.M.; Knulst, A.C.; Tibbe, G.J.M.; Oudenaren, A. van; Baert, M.R.M.; Benner, R.; Savelkoul, H.F.J. (1994) [Suppression of skin allograft rejection in mice by anti-CD3 monoclonal antibodies without cytokine-related side-effects](http://library.wur.nl/wasp/bestanden/LUWPUBRD_00336260_A502_001.pdf). *Transplantation 58 (1994). p. 257 - 261.*

**1993**

Oosterhout, A.J.M. van; Ark, I. van; Hofman, G.; Savelkoul, H.F.J.; Nijkamp, F.P. (1993) [Recombinant interleukin-5 induces in vivo airway hyperresponsiveness to histamine in guinea pigs](http://dx.doi.org/10.1016/0014-2999(93)90475-W). *Eur J Pharmacol 236 (1993). p. 379 - 383.*

Oosterhout, A.J.M.; Ladenius, A.R.C.; Savelkoul, H.F.J.; Ark, I. van; Delsman, K.C.; Nijkamp, F.P. (1993) [Effect of anti-IL-5 and IL-5 on airway hyperreactivity and eosinophils in guinea pigs](http://library.wur.nl/wasp/bestanden/LUWPUBRD_00336249_A502_001.pdf). *The American review of respiratory disease 147 (1993). p. 548 - 552.*

Savelkoul, H.F.J. (1993) Rol van cytokines in de regulatie van immuunrespons. *In: Het medisch jaar. / Es, J.C. van Mandema, E. Olthuis, G. Verstraete, M., . - Houten : Bohn Stafleu Van Loghum bv, p. 321 - 333.*

Wilsem, E. van; BrevÃƒ, J.; Hoogstraten, I. van; Savelkoul, H.; Kraal, G. (1993) [The influence of dendritic cells on T-cell cytokine production](http://library.wur.nl/wasp/bestanden/LUWPUBRD_00336246_A502_001.pdf). *In: Dendritic cells in fundamental and clinical immunology. / Kamperdijk, E.W.A. Nieuwenhuis, P. Hoefsmit, E.C.M., . - New York : Plenum Press, - p. 111 - 115.*

**1992**

Knulst, A.C.; Bril-Bazuin, C.; Tibbe, G.J.M.; Oudenaren, A. van; Savelkoul, H.F.J.; Benner, R. (1992) Cytokines in lethal graft-versus-host disease. *Transplant International 5 (1992). p. 679 - 680.*

Savelkoul, H.F.J. (1992) Interleukines from transfected cell lines. *In: Course manual "Cell culture for clinic and pharmaceutical industry". - Utrecht : University Hospital, 1992 - p. 1 - 6.*

**1991**

Benner, R.; Savelkoul, H.F.J. (1991) [Regulation of IgE production in mice](http://library.wur.nl/wasp/bestanden/LUWPUBRD_00336211_A502_001.pdf). *European Respiratory Journal 4 (1991). p. 97s - 104s.*

Knulst, A.C.; Bril-Bazuin, C.; Savelkoul, H.F.J.; Benner, R. (1991) [Suppression of graft-versus-host reactivity by a single host-specific blood transfusion to prospective donors of hemopoietic cells](http://library.wur.nl/wasp/bestanden/LUWPUBRD_00336210_A502_001.pdf). *Transplantation 52 (1991). p. 534 - 539.*

Savelkoul, H.F.J.; Plas, D. van der (1991) [Evaluation of the purity of synthetic oligonucleotides for PCR amplification by ion exchange FPLC](http://library.wur.nl/wasp/bestanden/LUWPUBRD_00336207_A502_001.pdf). *Science Tools: the L K B instrument journal 35: (1991). p. 1 - 3.*

Savelkoul, H.F.J.; Pathak, S.S.; Linde-Preesman, A.A. van der (1991) [Rapid purification of mouse IgE antibodies by multi-column liquid chromatography](http://library.wur.nl/wasp/bestanden/LUWPUBRD_00336208_A502_001.pdf). *In: Proceedings of the fourth FPLC symposium. - Woerden : Pharmacia, - p. 83 - 93.*

Savelkoul, H.F.J.; Linde-Preesman, A.A. van der (1991) Isolation of monoclonal antibodies from in vitro cultures of human B cell lines employing an automated three-column FLPC system. *In: Proceedings of the fourth FPLC symposium. - Woerden : Pharmacia, - p. 73 - 82.*

Savelkoul, H.F.J.; Seymour, B.W.P.; Sullivan, L.; Coffman, R.L. (1991) [IL-4 can correct defective IgE production in SJA/9 mice](http://www.jimmunol.org/content/vol146/issue6/). *Journal of Immunology 146 (1991). p. 1801 - 1805.*

Savelkoul, H.F.J.; Plas, D. van der; Helden-Meeuwsen, C.G. van (1991) Purity evaluation of synthetic oligonucleotides for PCR amplification by ion exchange FPLC. *In: Proceedings of the fourth FPLC symposium. - Woerden : Pharmacia, - p. 12 - 22.*

Savelkoul, H.F.J. (1991) Cytokines from transfected cells. *In: Course Book International Biotechnology: Cell culture for clinic and pharmaceutical industry. - Utrecht : [s.n.], 1991 - p. 1 - 3.*

**1990**

Benner, R.; Savelkoul, H.F.J. (1990) Regulation of the production of IgE. *In: Post Graduate Course Pollinosis 1990 Erasmus University, Rotterdam. - Rotterdam : Erasmus University, 1990 - p. 15 - 18.*

Savelkoul, H.F.J. (1990) Immunoblotting, chromatografie en electroforese. *Rotterdam : Afdeling Immunologie, Erasmus Universiteit Rotterdam., - p. 1 - 24.*

Savelkoul, H.F.J. (1990) [Immunologie](http://library.wur.nl/wasp/bestanden/LUWPUBRD_00336190_A502_001.pdf). *O'dokter 9 (1990) Issue. - p. 12 - 14.*

**1989**

Coffman, R.L.; Savelkoul, H.F.J.; Lebman, D.A. (1989) Cytokine regulation of immunoglobulin isotype switching and expression
*Seminars in immunology 1 (1989). p. 55 - 63.*

Pathak, S.S.; Vos, Q.; Savelkoul, H.F.J. (1989) [Terasaki-ELISA for murine IgE-anibodies III. determination of concentration and functional affinity by sequential equilibrium binding analysis](http://dx.doi.org/10.1016/0022-1759(89)90031-8). *Journal of Immunological Methods 123 (1989). - p. 71 - 81.*

Savelkoul, H.F.J.; Soeting, P.W.C.; Radl, J.; Linde-Preesman, A.A. van der (1989) [Terasaki-ELISA for murine IgE-antibodies.I.Quality of the detecting antibody: production and specificity testing of antisera specific for IgE](http://dx.doi.org/10.1016/0022-1759(89)90213-5). *Journal of Immunological Methods 116 (1989). - p. 265 - 275.*

Savelkoul, H.F.J.; Soeting, P.W.C.; Josselin de Jong, J.E. de; Pathak, S.S. (1989) [Terasaki-ELISA for murine IgE antibodies.II.Quantitation of absolute concentration of antigen-specific and total IgE](http://dx.doi.org/10.1016/0022-1759(89)90214-7). *Journal of Immunological Methods 116 (1989). - p. 277 - 285.*

Savelkoul, H.F.J.; Akker, T.W. van den; Soeting, P.W.C.; Oudenaren, A. van; Benner, R. (1989) [Modulation of total IgE levels in serum of normal and athymic nude BALB/c mice by cells and exogenous antigenic stimulation](http://library.wur.nl/wasp/bestanden/LUWPUBRD_00336157_A502_001.pdf). *International Archives of Allergy and Immunology 89 (1989). p. 113 - 119.*

**1988**

Coffman, R.L.; Seymour, B.W.P.; Lebman, D.A.; Hiraki, D.D.; Christiansen, J.A.; Shrader, B.; Cherwinski, H.M.; Savelkoul, H.F.J.; Finkelman, F.D.; Bond, M.W.; Mosmann, T.R. (1988) [The role of helper T cell products in mouse B cell differentiation and isotype regulation](http://library.wur.nl/wasp/bestanden/LUWPUBRD_00336145_A502_001.pdf). *Immunological Reviews 102 (1988). - p. 5 - 28.*

Linde-Preesman, A.A. van der; Savelkoul, H.F.J. (1988) An alternative method for immunoblotting on Phast System. *In: Pharmacia Separations. 1988 - p. 1 - 3.*

Savelkoul, H.F.J.; Termeulen, J.; Coffman, R.L.; Linde-Preesman, A.A. van der (1988) [Frequency analysis of functional Ig Ce gene expression in the presence and absence of interleukin 4 in lipopolysaccharide-reactive murine B cells from high and low IgE responder strains](http://library.wur.nl/wasp/bestanden/LUWPUBRD_00336138_A502_001.pdf). *European journal of immunology 18 (1988). p. 1209 - 1215.*

Savelkoul, H.F.J.; Pathak, S.S.; Sabbele, N.R.; Benner, R. (1988) [Generation and measurement of antibodies](http://library.wur.nl/wasp/bestanden/LUWPUBRD_00336139_A502_001.pdf). *In: Handbook of Experimental Pharmacology. Vol 85 / Bray, M.A. Morley, J., p. 141 - 185.*

Savelkoul, H.F.J.; Linssen, P.C.L.M.; Termeulen, J.; Linde-Preesman, A.A. van der; Benner, R. (1988) [Frequency analysis of functional immunoglobulin CE gene expression in LPS reactive murine B cells](http://library.wur.nl/wasp/bestanden/LUWPUBRD_00336140_A502_001.pdf). *In: Lymphocyte activation and differentiation. / Mani, J.C. Dornand, J., p. 585 - 589.*

Savelkoul, H.F.J.; Lebman, D.A.; Benner, R.; Coffman, R.L. (1988) [Increase of precursor frequency and clonal size of murine IgE-secreting cells by IL-4](http://www.jimmunol.org/cgi/reprint/141/3/749). *Journal of Immunology 141 (1988). p. 749 - 755.*

Savelkoul, H.F.J.; Greeve, A.A.M.; Rijkers, G.T.; Marwitz, P.A.; Benner, R. (1988) [Rapid procedure for coupling of protein antigens to red cells to be used in plaque assays by prewashing in chromium chloride](http://dx.doi.org/10.1016/0022-1759(88)90056-7). *Journal of Immunological Methods 111 (1988). - p. 31 - 37.*

Savelkoul, H.F.J. (1988) Quantitative ELISA: theoretical aspects and practical pointers. *Rotterdam : Afdeling Immunologie, Erasmus Universiteit Rotterdam., - p. 1 - 92.*

Savelkoul, H.F.J. (1988) Induction and measurement of IgE.A study in mice, with emphasis on the regulatory role of lymphokines. *Rotterdam : Erasmus University of Rotterdam, the Netherlands,*

**1987**

Savelkoul, H.F.J.; Pathak, S.S.; Linde-Preesman, A.A. van der (1987) [Occurrence of damaged heavy chains during purification of murine IgE antibodies by fast protein liquid chromatography (FPLC) and their effect on the determination of concentration and affinity in ELISA](http://library.wur.nl/wasp/bestanden/LUWPUBRD_00336118_A502_001.pdf). *Protides of the biological fluids 35 (1987). - p. 375 - 382.*

Savelkoul, H.F.J.; Pathak, S.S.; Linde Preesman, A.A. van der (1987) [Rapid purification of mouse IgE antibodies by multi column liquid chromatography](http://library.wur.nl/wasp/bestanden/LUWPUBRD_00336119_A502_001.pdf). *In: In: Proceedings second FPLC symposium. - Woerden : Pharmacia Nederland, - p. 83 - 93.*

Savelkoul, H.F.J.; Linde-Preesman, A.A. van der (1987) [Isolation of monoclonal antibodies from in vitro cultures of human B cell lines employing an automated three-column FPLC system](http://library.wur.nl/wasp/bestanden/LUWPUBRD_00336120_A502_001.pdf). *In: Proceedings second FPLC symposium. - Woerden : Pharmacia Nederland, - p. 73 - 82.*

**1985**

Savelkoul, H.F.J.; Soeting, P.W.C.; Benner, R.; Radl, J. (1985) [Quantitation of murine IgE in an automatic ELISA system](http://library.wur.nl/wasp/bestanden/LUWPUBRD_00336098_A502_001.pdf). *Advances in experimental medicine and biology 186 (1985). p. 757 - 765.*

Savelkoul, H.F.J.; Greeve, A.A.M.; Soeting, P.W.C.; Benner, R.; Radl, J. (1985) [Isolation, characterization and quantitation of murine IgE](http://library.wur.nl/wasp/bestanden/LUWPUBRD_00336099_A502_001.pdf). *Protides of the biological fluids 33 (1985). - p. 611 - 614.*

**1983**

Nikkels, P.G.J.; Bril, H.; Savelkoul, H.F.J.; Oudenaren, A. van; Ploemacher, R.E. (1983) [Short term immunosuppressive effects of Cis-diaminedichloroplatinum (II) (DDP) in mice](http://library.wur.nl/wasp/bestanden/LUWPUBRD_00336082_A502_001.pdf). *In: Modern Trends in Clinical Immunosuppression. / Weimar, W. Marquet, R.L. Bijnen, A.B. Ploeg, R.J., . - Rotterdam. : Dept.Renal Transplantation, - p. 167 - 174.*

**1982**

Lonai, P.; Arman, E.; Savelkoul, H.F.J.; Friedman, V.; Puri, J.; Hammerling, G. (1982) [Factors, receptors, and their ligands: studies with H-2 restricted helper hybridoma clones](http://library.wur.nl/wasp/bestanden/LUWPUBRD_00336077_A502_001.pdf). *In: Isolation, characterization, and utilization of T lymphocyte clones. / Fathman, C.G. Fitch, F.W., . - New York : Academic Press, - p. 109 - 117.*

**1981**

# Lonai, P.; Savelkoul, H.F.J.; Puri, J.; Hammerling, G. (1981) [Two separate genes regulate self-Ia and carrier recognition in H-2-restricted helper factors secreted by hybridoma cells](http://dx.doi.org/10.1084/jem.154.6.1910). *The journal of experimental medicine 154 (1981). p. 1910 - 1921.*

**Curriculum vitae**

# Hans VERHOEF, PhD (Field coordinator)

| **Personal data:** | |
| --- | --- |
| Full name: | Johannes Christiaan Maria VERHOEF |
| Address: | Cell Biology and Immunology Group, Wageningen University, P.O. Box 338, 6700 AH Wageningen, The Netherlands. Tel: + 31 317 483509; e-mail: [hans.verhoef@wur.nl](mailto:hans.verhoef@wur.nl) |
| Place/date of birth: | Bergh, The Netherlands/23 September 1960 |
| Nationality: | Dutch |
|  | |
| **Academic qualifications:** | |
| Graduation expected in August 2005 | **MSc, clinical epidemiology**  Erasmus University Rotterdam, The Netherlands |
| September 2001 | **PhD degree**  (Thesis: Iron supplementation and malaria as determinants of anaemia in African children) Wageningen University, The Netherlands |
| September 2001 | **Certificate, Human Nutrition**  Graduate School for Advanced Studies in Food Technology, Nutrition and Health Sciences (VLAG), The Netherlands |
| January 1988 | **MSc degree, agricultural and environmental sciences**  Wageningen University, The Netherlands |
|  | |
| **Work experience:** | |
| 2004-present | **Research fellow**  Cell Biology and Immunology Group, Wageningen University, The Netherlands |
| 2002 – 2003 | **Research fellow**  Division of Human Nutrition, Wageningen University, The Netherlands |
| 2001 –2002 | **Research fellow**  Graduate School for Advanced Studies in Food Technology, Nutrition and Health Sciences (VLAG), The Netherlands |
| 1996 – 2000 | **PhD fellow**  African Medical and Research Foundation, Kenya/Division of Human Nutrition and Epidemiology, Wageningen University, The Netherlands |
| 1994-1996 | **Free lance consultant** |
| 1994-1995 | **Research fellow**  Department of Epidemiology and Public Health, Wageningen University, The Netherlands |
| 1991-1994 | **Medical entomologist**  WHO/FAO/UNEP/UNCHS Panel of Experts on Environmental Management for Vector Control (PEEM), World Health Organization, Geneva, Switzerland |
| 1988-1991 | **Medical entomologist**  Malaria Control Project, World Health Organization, Solomon Islands |
|  | |
| **Publications:** | |
| 1. **Verhoef H,** West CE. Dietary factors that determine bioavailability of micronutrients: targets for breeding? (submitted) 2. **Verhoef H,** West CE. Measuring iron status, with special reference to children in developing countries (submitted). 3. Umeta M, West CE, **Verhoef H,** Haidar J, Hautvast JG. Long-term effects of zinc supplementation on growth of children in rural Ethiopia: a randomized controlled trial (submitted). 4. Cong Khan N, West CE, De Pee S, Hulshof PJ, Huy Khoi H, **Verhoef H,** Hautvast JG. A randomised controlled trial to determine how much plant foods can contribute to the vitamin A supply of lactating women in Vietnam (submitted). 5. **Verhoef H,** Veenemans J, Van Laatum S, West CE. Maternal screening for fever in African children without thermometer (submitted). 6. **Verhoef H,** Rijlaarsdam J, Burema J, West CE, Kok FJ. Variance estimation when using cluster surveys in developing countries to assess exposure-health outcome relationships (submitted). 7. **Verhoef H,** Hogervorst R, West CE, Van den Broek NR, Kok FJ. Assessment and application of a new, simple test to detect anaemia in developing countries (submitted). 8. Dillon D, **Verhoef H,** West CE, Meester H, Bos M, Hautvast JG. The effects of additional vitamin A or riboflavin on hemoglobin and iron status among anemic Indonesian adolescent schoolgirls receiving iron supplements (submitted). 9. **Verhoef H,** West CE. Validity of the relative dose-response test and the modified relative dose-response test as indicators of vitamin A stores in liver. Am J Clin Nutr, in press. 10. **Verhoef H,** West CE, Bleichrodt N, Dekker PH, Born MP. Effects of micronutrients during pregnancy and early infancy on mental and psychomotor development. In: Micronutrient deficiencies in the first months of life (Delange FM, West KP, eds.). Nestlé Nutrition Workshop Series Pediatric Program, Vol 52 (Dubai, United Arab Emirates: October 13-17, 2002). Vevey, Switzerland: Nestec/S Karger, Basel, 2003; 325-355. 11. **Verhoef H,** West CE. Anemia in African children: malaria or iron deficiency? Lancet 2003;361:2249-50. 12. Umeta M, West CE, **Verhoef H,** Haidar J, Hautvast JG. Factors associated with stunting in infants aged 5-11 months in the Dodota-Sire district, rural Ethopia. J Nutr 2003;133:1064-69. 13. **Verhoef H.** Divine intervention. Lancet 2002;360:1990. 14. **Verhoef H,** West CE, Nzyuko SM, et al. Intermittent administration of iron and sulfadoxine-pyrimethamine to control anaemia in Kenyan children: a randomised controlled trial. Lancet 2002;360:908-14. 15. **Verhoef H,** West CE, Kraaijenhagen R, et al. Malarial anaemia leads to adequately increased erythropoiesis in asymptomatic Kenyan children. Blood 2002;100:3489-94. 16. **Verhoef H,** West CE, Veenemans J, Beguin Y, Kok FJ. Stunting may determine the severity of malaria-associated anemia in African children. Pediatrics 2002;110:E48. 17. Van Crevel R, Karyadi E, Netea MG, **Verhoef H,** Nelwan RH, West CE, Van der Meer JW. Decreased plasma leptin concentrations in tuberculosis patients are associated with wasting and inflammation. J Clin Endocrin Metab 2002;87:758-63. 18. Eilander A, Schmidt M, **Verhoef H.** Voeding en gezondheid in ontwikkelingslanden. [Nutrition and health in developing countries]. Voeding Nu 2002;4:19-21. 19. **Verhoef H,** West CE, Ndeto P, Burema J, Beguin Y, Kok FJ. Serum transferrin receptor concentration indicates increased erythropoiesis in Kenyan children with asymptomatic malaria. Am J Clin Nutr 2001;74:767-75. 20. **Verhoef H,** Veenemans J, West CE. HIV-1 infection and malaria parasitaemia. Lancet 2001;357:232-33. 21. **Verhoef H.** Iron deficiency and malaria as determinants of anaemia in African children. PhD thesis. Wageningen, The Netherlands: Wageningen University, 2001. 22. **Verhoef H,** West CE, Kok FJ. Phenobarbital use in children with cerebral malaria. Lancet 2000; 356:256. 23. Malaria: a manual for community health workers. Geneva, Switzerland: World Health Organization, 1997 [edited version of a manual developed in the Solomon Islands]. 24. **Verhoef H,** Hodgins E, Eggelte TA et al. Anti-malarial drug use among preschool children in an area of seasonal malaria transmission in Kenya. Am J Trop Med Hyg 1999;61:770-75. 25. **Verhoef H,** Hodgins E, West CE, et al. Diagnosis of fever in Africa. Lancet 1998;351:372-73. 26. **Verhoef H.** Irrigation and vector-borne diseases in Africa. In: Effets sanitaires de l'irrigation et des aménagements hydro-agricoles (B Margraf, ed.). Heidelberg Studies in Applied Economics and Rural Institutions, Publications of the Research Centre for International Agrarian and Economic Development No. 28 (Gans O, ed.). Saarbrücken, Germany: Verlag für Entwicklungspolitik, 1996:17-40. 27. **Verhoef H.** Health aspects of Sahelian floodplain development. In: Water management and wetlands in Sub-Saharan Africa (Acreman MC, Hollis GE, eds.). Gland, Switzerland: World Conservation Union, 1995. 28. **Verhoef H.** Rice cultivation and human diseases: the scope for rice ecosystem management for vector control. In: World Agriculture 1994. London, UK: Sterling Publications, 1994:102-05. 29. **Verhoef H,** Hoekstra FA. Absence of 10-hydroxy-2-decenoic acid (10-HDA) in bee-collected pollen. In: Biotechnology and ecology of pollen. University of Massachusetts, Amherst, MA (eds. Mulcahy DL, Mulcahy GB, Ottaviano E). New York, USA: Springer-Verlag, 1986:391-96. | |

**Jacobien VEENEMANS, MD (PhD Fellow in clinical/nutritional aspects of the study)**

| **Personal data:** | |
| --- | --- |
| Full name: | Jacobien VEENEMANS |
| Address: | Cell Biology and Immunology Group, Wageningen University, P.O. Box 338, 6700 AH Wageningen, The Netherlands. Tel: + 31 317 483509; e-mail: [jacobien.veenemans@wur.nl](mailto:jacobien.veenemans@wur.nl) |
| Place/date of birth: | Laren NH, The Netherlands/12 August 1974 |
| Nationality: | Dutch |
|  | |
| **Academic qualifications:** | |
|  | Resident in internal medicine, University Medical Centre Nijmegen (started: 1 November 2003). Residency training will be integrated with the PhD work (‘AGIKO’ construction) |
| July 2002 | Medical Doctor, University of Amsterdam, The Netherlands |
|  | |
| **Work experience:** | |
| July 2003 – present | **Physician**  Department of Internal Medicine, Hospital ‘Gelderse Vallei’, Ede, The Netherlands |
| 2002 - 2003 | **Editorial assistant**  Nederlands Tijdschrift voor Geneeskunde (Netherlands Journal for Medicine) |
| 1996, 2000 | Internships in India and Kenya |
|  | |
| **Publications:** | |
| 1. Verhoef H, **Veenemans J,** Van Laatum S, West CE. Maternal screening for fever in African children without thermometer (submitted). 2. Verhoef H, West CE, Nzyuko SM, De Vogel S, Van der Valk R, Wanga MA, Kuijsten A, **Veenemans J,** Kok FJ.. Intermittent administration of iron and sulfadoxine-pyrimethamine to control anaemia in Kenyan children: a randomised controlled trial. Lancet 2002;360:908-14. 3. Verhoef H, West CE, **Veenemans J,** Beguin Y, Kok FJ. Stunting may determine the severity of malaria-associated anemia in African children. Pediatrics 2002;110:E48. 4. Verhoef H, **Veenemans J,** West CE. HIV-1 infection and malaria parasitaemia. Lancet 2001;357:232-33. | |

**Erasto MBUGI, MSc (PhD Fellow in immunological aspects of the study)**

| **Personal data:** | |
| --- | --- |
| Full name: | Erasto Vitus MBUGI |
| Address: | Muhimbili University College of Health Sciences,  Faculty of Medicine, Department of Biochemistry,  P. O. Box 65001, Dar es Salaam. Tel: +255 (0) 741 418279 |
| Place/date of birth: | 5 January 1972/Kibaha, Tanzania |
| Nationality: | Tanzanian |
|  | |
| **Academic qualifications:** | |
| November 2004 | **Master of Veterinary Medicine (MVM)**  Sokoine University of Agriculture (SUA), Morogoro, Tanzania. Specialisation: biochemistry and molecular biology. MVM thesis: Investigation of multiplicity of infections and drug resistance to *Plasmodium falciparum* malaria using the MSP2, DHFR and DHPS genes as target genes. |
| July 1999 | **Bachelor of Veterinary Medicine (BVM)**  Sokoine University of Agriculture (SUA), Morogoro, Tanzania |
|  | |
| **Work experience:** | |
|  |  |
| August 2001 -present: | **Tutorial Assistant**  Department of Biochemistry, Faculty of Medicine, Muhimbili University College of Health Sciences (MUCHS), Morogoro |
| 2000 - 2001 | **Veterinary Officer**  Moshi Police Training School |
| 1999 -2000 | **Veterinary Consultant**  Dar Vetcare |

**Curriculum vitae**

Professor Raimos M. Olomi

Name: Raimos Moses OLOMI

Year of birth: 1944

Sex: Male

Marital status: Married

Designation: Director of Post-Graduate Studies

Institutional address: KCMC College

P.O. Box 2240, Moshi, Tanzania

E-mail address: [raimosolomi@hotmail.com](mailto:raimosolomi@hotmail.com)

Telephone: + 255 27 2754377 ext. 443, res. + 255 27 2753220

Fax: + 255 27 2754381

Degrees: MD University of Dar es Salaam 1967-72

MMed University of Dar es Salaam 1975-82

MPH Royal Tropical Institute, Amsterdam 1988-89

Cert. Neonatology, Jessops Hospital for Women, Sheffield 1981-82

**Positions held:**

- 1. District Medical Officer, Newala District
  2. Regional Medical Officer, Mara Region, Tanzania

1993-on Head of Paediatrics Department KCMC

1993-on Coordinator, Control of Diarrhoeal Disease, Northern Zone, Tanzania

1998-on Coordinator, Postgraduate Training and Research, KCMC College

1999-on Professor of Paediatrics, KCM College

2002-on Coordinator, Joint Malaria Programme (KCMC)

2004-on Tumaini University Senate Committee Member

**Other relevant skills/experiences:**

Teacher for several years at KCMC Hospital and KCM College

Examiner Undergraduate/Postgraduate level

Supervisor several dissertations in MMed/MPH

Board Member of several bodies at KCM College and outside KCM College

**Publications**:

Bergsjo P, Olomi RM, Talle A, Klepp KI. Bar attendants as health educators: prevention of sexually transmitted diseases in high-risk areas. Norwegian Medical Journal 1995 Oct 30:115(6):3281-3.

Klingenberg C, Olomi R, Oneko M, Sam N, Langeland N, Neonatal morbidity and mortality in a Tanzanian tertiary care referral hospital. Ann Trop Paediatr 2003 Dec;23(4):293-9.

Reyburn H, Olomi R, et al. (2003) Association of transmission intensity and age with clinical manifestations and case fatality of severe Plasmodium malaria. JAMA Vol. 293, 1461-70.

Reyburn H, Olomi R, et al. (2005) Over-diagnosis of malaria in patients with severe febrile illness in Tanzania prospective study. BMJ online first bms.com, 1-6.

HIV intervention in high transmission area in Arusha: experiences from a mining community, co-author of a chapter in a book. “Young people at risk”. Fighting AIDS in Northern Tanzania.

**Scientific papers presented:**

Child abuse among the tribes on the Eastern coast of Lake Victoria Tanzania, paper presented at 20^th^ Annual Scientific Conference of the Medical Association of Tanzania, Dar es Salaam, 1994 – Proceedings of the 20^th^ Annual Scientific Conference, Special Issue September, 1985, Tanzania Medical Journal

Self medication in the management of minor illnesses in children: paper presented at the 20^th^ Annual Scientific Conference of the Medical Association of Tanzania, Dar es Salaam, 1994 – Proceedings of the 20^th^ Annual Scientific Conference, Special Issue September, 1985, Tanzania Medical Journal

Alcohol and its related social and psychiatric problems in Mara region of Tanzania: paper presented at the 23^th^ Annual Scientific Conference of the Medical Association of Tanzania, Dar es Salaam, 1987 – Proceedings of the 20^th^ Annual Scientific Conference, Special Issue September, 1985, Tanzania Medical Journal

Community based HIV/high transmission area intervention in Northern Tanzania: paper presented at the 12^th^ World AIDS Conference Geneva, 28^th^ July, 1998. Abstract number 0474.

1. This is tentative; our choice for chewable tables or nutrient-dense spread will be made in consultation with the UN Children’s Fund. [↑](#footnote-ref-2)
2. To be decided. [↑](#footnote-ref-3)
3. To be provided in consultation with local government and health personnel. [↑](#footnote-ref-4)
4. To be provided in consultation with local government and health personnel. [↑](#footnote-ref-5)
5. IOM. Dietary reference intakes for vitamin C, vitamin E, selenium, and carotenoids. Washington DC: Institute of Medicine, 2000.

   IOM. Dietary reference intakes for thiamin, riboflavin, niacin, vitamin B6, folate, vitamin B12, pantothenic acid, biotin, and choline. Washington DC: Institute of Medicine, 2000.

   IOM. Dietary reference intakes for vitamin A, vitamin K, arsenic, boron, chromium, copper, iodine, iron, manganese, molybdenum, nickel, silicon, vanadium, and zinc. Washington DC: Institute of Medicine, 2002. [↑](#footnote-ref-6)
6. 1 RDA represents the average daily dietary intake level that the IOM considers sufficient to meet the nutrient requirement of 97.5% of healthy children in Canada or the USA [↑](#footnote-ref-7)
7. Bhandari N, et al. BMJ 2002;324:1358. Penny ME, et al. Am J Clin Nutr 2004;79:457-65. Brooks WA, et al. Lancet 2004;363:1683-88. Castillo-Duran C, et al. J Pediatr 2001;138:229-35. Kikafunda JK, et al. Am J Clin Nutr 1998; 68:1261-66. Muller O, et al. Int J Epidemiol 2003;32:1098-102. Sazawal S, et al. N Engl J Med 1995;333:839-44. Roy SK. Glimpse 1991;13:2. Bhutta ZA et al. Pediatrics 1999;103:e42. Umeta M, et al. Lancet 2000;355:2021-26. [↑](#footnote-ref-8)
8. Fischer Walker C, Kordas K, Stoltzfus RJ, Black RE. Interactive effects of iron and zinc on biochemical and functional outcomes in supplementation trials. Am J Clin Nutr 2005;82:5-12. [↑](#footnote-ref-9)
9. Hurrell RF. Fortification: overcoming technical and practical barriers. J Nutr 2002;132:806S-12S; Hurrell RF. How to ensure adequate iron absorption from iron-fortified food. Nutr Rev 2002:60:S7-S15. [↑](#footnote-ref-10)
10. Ranade VV, Somberg JC. Bioavailability and pharmacokinetics of magnesium after administration of magnesium salts to humans. Am J Therap 2001;8:345-57. [↑](#footnote-ref-11)
11. Hanzlik RP, Fowler SC, Fisher DH. Relative bioavailability of calcium from calcium formate, calcium citrate, and calcium carbonate. J Pharmacol Experim Ther 2005;313:1217-22. [↑](#footnote-ref-12)
12. Lonnerdal B. Dietary Factors Influencing Zinc Absorption. J Nutr 2000;130:1378S-83S. [↑](#footnote-ref-13)
13. Wapnir RA. Copper absorption and bioavailability. Am J Clin Nutr 1998(suppl);67:1054S–60S. [↑](#footnote-ref-14)
14. One RDA represents the average daily dietary intake level that the US Institute of Medicine considers sufficient to meet the nutrient requirement of 97.5% of healthy children in Canada or the USA. [↑](#footnote-ref-15)
